# Electrochemical Synthesis of Isolated Fluoride Reagents from PFAS

**DOI:** 10.1021/jacs.6c01373

**Published:** 2026-03-09

**Authors:** Florian Dorchies, Luke P. Ward, Isaac P. Richards, Mohamed Elsherbini, Alastair J. J. Lennox

**Affiliations:** School of Chemistry, 1980 University of Bristol, Cantock’s Close, Bristol, Avon BS8 1TS, U.K.

## Abstract

Per- and polyfluoroalkyl substances (PFAS) are of increasing concern due to their environmental persistence and adverse effects on health. The recovery of fluoride from the decomposition of concentrated sources of PFAS (e.g., refrigerants, protective coatings, and foams) enables a circular economy. Recent efforts have largely focused on one-pot, transfer fluorination strategies, with limited examples of forming isolable fluoride reagents, which represent the broadest opportunities for valorization. Here, we establish a direct electrochemical strategy to recycle nonpolymeric PFAS into a range of synthetically important fluoride reagents. The choice of electrolyte and solvent is critical in controlling whether fluoride or bifluoride ions are generated, enabling selective access to distinct reagents. These findings establish electrochemistry as a powerful and versatile platform for transforming environmentally persistent PFAS waste into valuable chemical resources.

## Graphical abstract

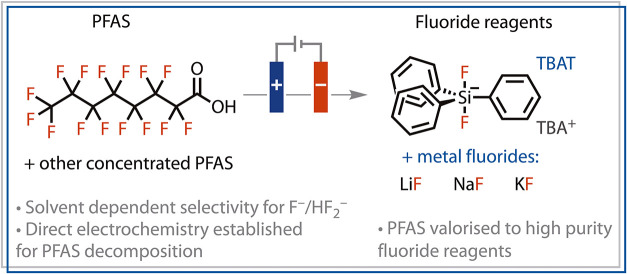

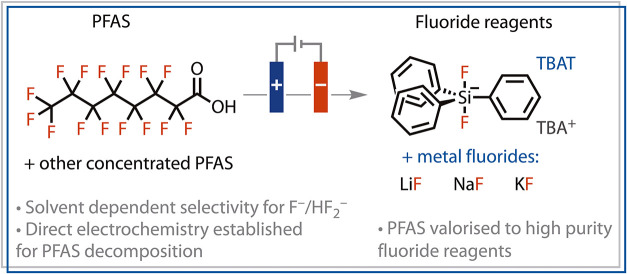

## Introduction

Per- and polyfluoroalkyl substances (PFAS) are an anthropogenic class of compounds that are, uniquely, both hydrophobic and lipophobic, which renders them resistant to both water and oil. The very strong C–F bonds provide extremely high chemical inertness and thermal stability. This set of properties has led to a rapid expansion in nonstick and heat and chemical resistant applications.^1^ However, these properties also lead to persistence in the environment and subsequent bioaccumulation, as shown in Figure 1A. The long-term effects of these so-called ‘forever chemicals’ on human health are a real and growing concern.^2,3^

**Figure 1:**
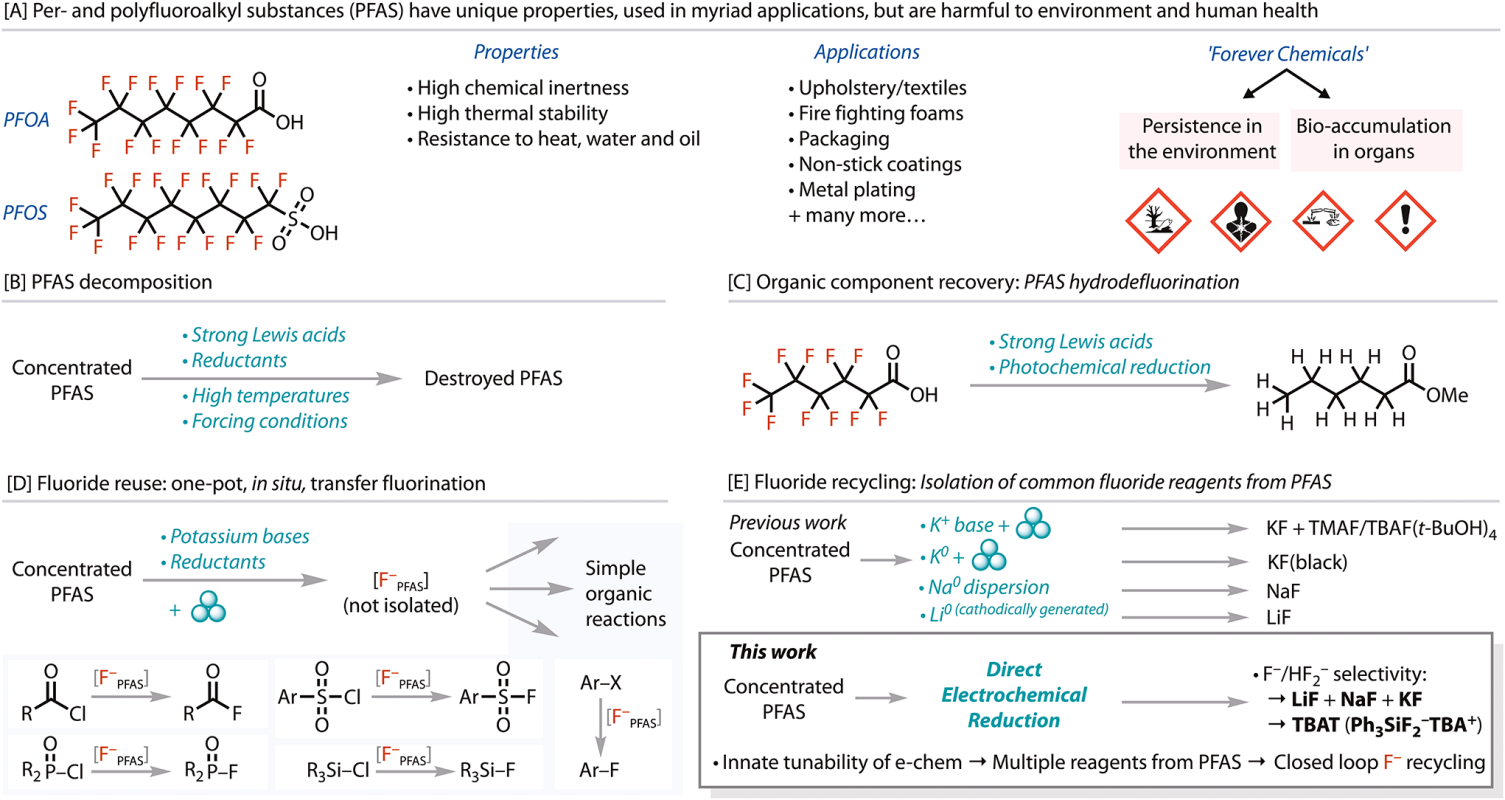
Strategies to manage concentrated sources of PFAS. (A) PFAS properties, applications, and their associated hazards. Strategies to manage concentrated sources of PFAS are (B) direct decomposition of the PFAS; (C) hydrodefluorination of PFAS to recover the organic component; (D) one-pot, *in situ*, reuse of the fluoride from PFAS; or (E) for PFAS recycling, where commonly used fluoride reagents can be isolated. We show in this work that using the innate tunability of electrochemistry, we can expand the range of compounds PFAS can be recycled into with a single technique.

Technologies for the remediation of contaminated water supplies, where PFAS are in ppm and ppb concentrations, have received substantial attention in recent years. Strategies have included the use of ultrasound,^4,5^ electrochemistry,^6–10^ photolysis,^11–14^ plasma,^15^ oxidation,^16^ and thermal treatments,^17,18^ which can be applied to municipal water purification systems and industrial scale roll out. However, different technologies are required to decompose the concentrated sources of PFAS that exist,^19^ such as refrigerants, clothing, protective coatings, or foams. Such is the challenge to break the very strong C–F bonds (BDE = 106–123 kcal/mol)^14^ in PFAS, that the developed strategies are highly energetic and/or complex, including the use of high temperatures and forcing conditions,^20^ strong Lewis acids,^21^ or reductants,^22–24^ Figure 1B. While degradation technologies are important, recovering the organic component of PFAS offers an attractive opportunity to reclaim value from this waste product. Strong Lewis acids^25,26^ and photochemical reduction^27^ strategies have proven effective at hydrodefluorination of PFAS, Figure 1C.

Recycling the fluorine component from banks of concentrated sources of PFAS provides a means to alleviate pressure on the mining and processing of Fluorspar (CaF_2_), which has been assigned a “Critical Raw Material”,^28,29^ due to its importance to society and relative scarcity. Indeed, Fluorspar is the source of all organic and inorganic fluorinating reagents that are essential for the synthesis of myriad important molecules, including pharmaceuticals, agrochemicals, and battery additives.^19,30^ Fluorine has a unique ability to tune function and improve the value of these molecules. Hence, strategies for the recycling of PFAS to harvest fluorine are an essential and urgent endeavor.

Several technologies have recently been reported that extract the fluoride from PFAS and directly reuse it *in situ* in a variety of simple, one-pot, organic reactions, Figure 1D. This transfer-fluorination strategy^19^ has been demonstrated with the use of potassium bases^31–33^ or reductants,^34,35^ with mechanochemistry playing an important role in several of these methods.

Recycling fluoride into the broader and more widely used applications and molecules that it is found in, however, requires a transition from the one-pot, *in situ*, use of PFAS-fluoride to the isolation of commonly used fluoride reagents. This approach represents the most effective strategy to impact a real circular fluorine economy. Efforts toward this goal include the use of potassium-based reagents with mechanochemistry^32,36,37^ or a sodium metal dispersion,^38^ to isolate KF or NaF, respectively, Figure 1E. Very recently, a study toward the isolation of LiF was reported using electrochemically generated Li^0^ metal, which is electroplated onto the cathode surface to reduce PFAS,^39^ with control reactions showing the use of Li^0^ metal worked equally as well. Hence, the extraction of PFAS-fluoride into several widely used reagents using a single technique remains an important challenge.

We were motivated to develop a general method from which a range of conventional fluorination reagents could be generated and isolated from nonpolymeric PFAS degradation, Figure 1E. Considering the ease with which highly reducing potentials can be applied, the use of direct electrochemistry in this context is highly underdeveloped, especially for concentrated sources of PFAS. Hence, building on our previous work on electrochemical hydrodefluorination of −CF_3_ groups,^40,41^ we sought to exploit, for the first time, the innate and facile tunability of electrochemical reduction to recycle PFAS into useful and common fluorination reagents. The use of electrochemistry also has important advantages because it is safer and more easily scaled than approaches that rely on the use of stoichiometric metal reductants. Herein, we demonstrate the successful realization of this goal by showcasing how a range of commonly used fluorination reagents can be isolated in high purity by using direct electrochemical reduction. This includes the widely used reagent tetrabutylammonium difluorotriphenylsilicate (TBAT), which has not been accessed from PFAS before. Hence, this work expands the range of compounds into which PFAS can be recycled with a single technique, thus providing a critical solution to current PFAS recycling limitations.

## Results and Discussion

PFAS decomposition and fluoride recovery was initiated by undertaking preliminary studies using our recently reported counter-electrode process for nonaqueous electrochemical reduction reactions in an undivided cell.^42^ This metal-free and scalable counter-electrode process relies on the oxidation of bromide ions to tribromide ions that are trapped by triethylsilane (TESH) to form reductively stable TESBr and efficiently replaces the need for sacrificial anodes or divided cells. We hypothesized that by coupling this process with PFAS reduction in an undivided cell, electrogenerated fluoride ions could be trapped by TESBr to form TESF, which is a commercially available compound used in a range of industrial applications,^43^ Figure 2A.

**Figure 2:**
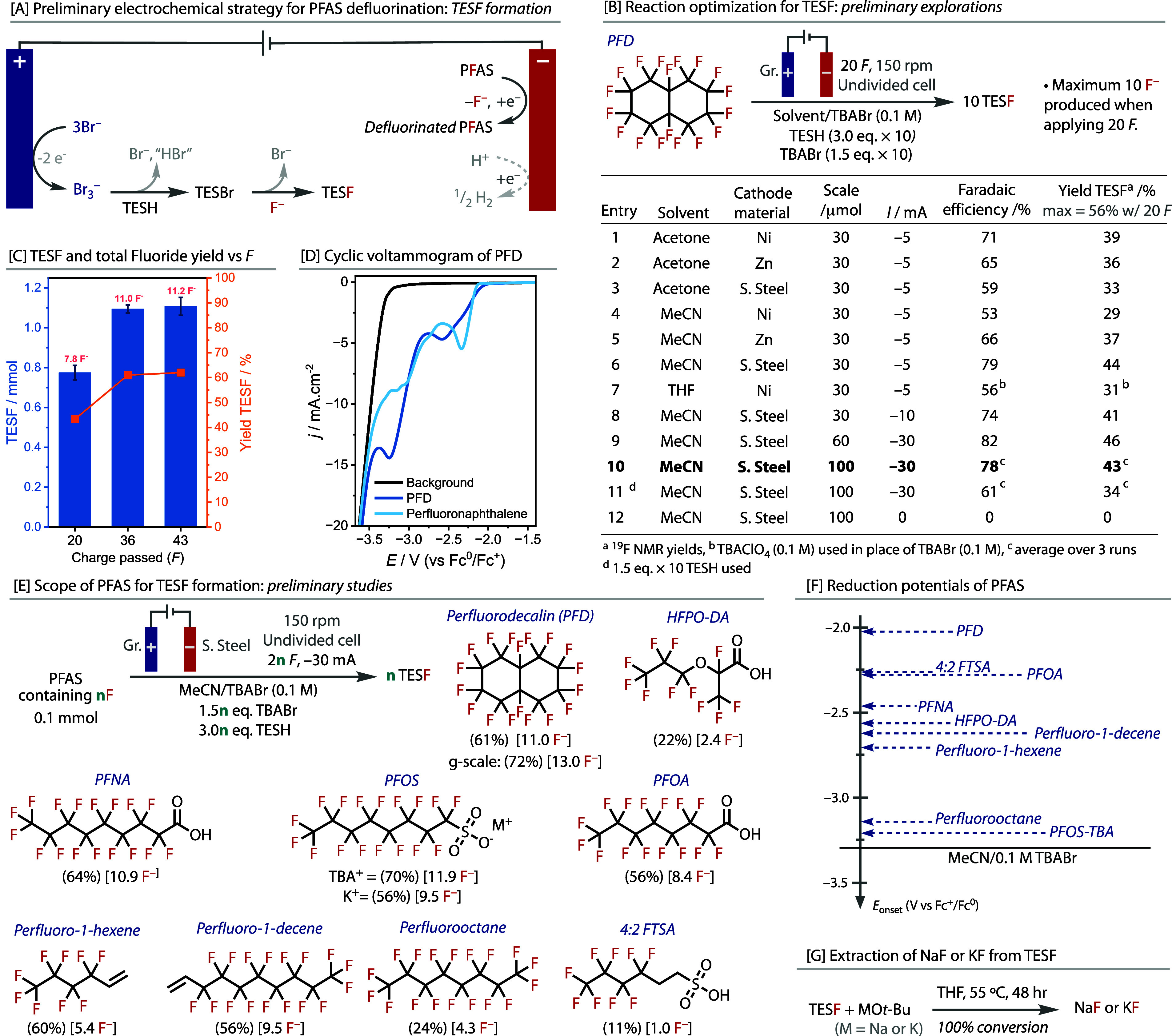
Preliminary studies in undivided cells: synthesis of TESF. (A) Our strategy for PFAS defluorination using an undivided cell based on a bromide-mediated silane-oxidation counter-electrode process. (B) Optimization of undivided cell conditions for TESF synthesis. (C) The yield and number of fluorines liberated as a function of equivalents of charge passed (*F*). The error bars represent the standard deviation over 3 runs, and yields are averaged over 3 runs. (D) CV of PFD and perfluoronaphthalene, both 5 mM in MeCN/TBABr (0.1 M). (E) PFAS substrate scope for TESF synthesis in undivided cells. ^19^F NMR yields are given. −30 mA applied current is ca. 18.8 mA.cm^–2^ (see SI). Electrolyses typically took ∼1 h 30 min to ∼3 h 15 min depending on the number of fluorines present in the PFAS. For the gram-scale reaction, 1.0 mmol of PFD and −50 mA were used, which led to a reaction time of 19 h 18 min. The cell potential (*E*_cell_) was ca. 4–5 V, irrespective of the PFAS. HFPO–DA = hexafluoropropylene oxide dimer acid; 4:2 FTSA = 4:2 fluorotelomersulfonic acid; PFOA = perfluorooctanoic acid; PFOS = perfluorooctanesulfonic acid; PFNA = perfluorononanoic acid. (F) Reduction onset potentials for each PFAS studied. (G) Generation of NaF and KF from TESF.

Perfluorodecalin (PFD), which is widely used in cosmetics and medicine for its high oxygen-carrying capacity, was chosen as a model PFAS for the generation of TESF,^44^ Figure 2B. For the purposes of optimization only, we limited the equivalents of charge to 20 *F*, which would allow for the maximum removal of 10 fluorine atoms from PFD, alongside the reduction of HBr that is generated in an equivalent quantity. A variety of solvents and cathode materials were tested (entries 1–8), where it was found that MeCN with stainless steel worked the best. An increase in the applied current improved the yield (entry 9) to deliver TESF with 82% Faradaic efficiency and 46% yield from a maximum of 56% (10 fluorine from a total of 18 after passing 20 *F*). These results were maintained within error when the PFAS concentration increased (entry 10). A decrease in the TESH equivalents negatively impacted the yield (entry 11), and the reaction did not proceed without electricity (entry 12).

Increasing the equivalents of charge passed (*F*) demonstrated that on average up to 11 fluorine atoms in PFD can be harnessed and converted to TESF, equating to a 61% yield, Figure 2C. ^19^F NMR showed the crude reaction mixture to be very clean, as no fluorinated species were present other than TESF, Figure S8.1, including the aromatized perfluoronaphthalene intermediate^45^ that we discovered has a similar reduction potential to PFD itself, Figure 2D. Given that no deposit was visible on the electrodes and there was no formation of insoluble particles, we propose that small gaseous (fluoro)carbon compounds are generated from the breakdown of the carbon backbone of PFAS.

Using the optimized conditions, passing the equivalents of charge (*F*) according to the number of fluorine atoms contained in each PFAS, these preliminary studies were extended to test a scope of concentrated PFAS, as shown in Figure 2E. Perfluorinated compounds with various chain lengths were selected, including some of the most widespread compounds, such as PFNA, PFOA, and salts of PFOS. Overall, TESF was obtained in good to very good yields throughout, Figure 2E and Figures S8.1–11. Lower yields were expected for shorter chain length PFAS, such as 4:2 FTSA and perfluoro-1-hexene, due to their increased C–F bond strength.^14,39^ However, the reaction performed surprisingly well with the small-chain alkenyl PFAS, which may be due to intermediate alkene bromination by electrogenerated Br_3_^–^/Br_2_ that may aid further electrochemical reduction. An even smaller PFAS, trifluoroacetic acid (TFA), was attempted, but complete retention of starting material was observed, Figure S8.12. For some PFAS, smaller fluorinated chains from their parent structure were observed, Figures S8.1–11. Pleasingly, the reaction of PFD scaled seamlessly to the gram scale without loss in yield of TESF.

Cyclic voltammetric analysis of each PFAS tested demonstrated that the optimized electrolyte of MeCN/TBABr provided an electrochemical potential window that could accommodate the deeply reductive potentials required for the dissociative electron transfer process,^6^ Figure 2F and Supporting Information 2.

The activation of TESF with bases was tested as a potential strategy to extract the fluoride to form more common and widely used fluoride reagents. We were pleased to discover that TESF could be fully converted to either NaF or KF upon treatment with NaO*t*-Bu or KO*t*-Bu, respectively, in THF at 55 °C, Figure 2G and Supporting Information 3, thereby demonstrating a route from PFAS, albeit indirect, to these important reagents.

With these preliminary studies in hand, we turned to develop a *direct* method of metal fluoride generation to build on our indirect method from TESF. Hence, we investigated the direct reduction of PFAS in a divided cell and in the absence of silane. Tetrabutylammonium bromide (TBABr) remained as the supporting electrolyte salt to avoid the potential of liberating other fluoride ions from fluorinated anions and to avoid the use of perchlorate salts that are difficult to dry. Variation of the solvent revealed highly interesting reaction outcomes. In acetone and methyl ethyl ketone (MEK), very good yields of fluoride were observed, Figure 3A, which were independent of the cathode material used; hence stainless steel was chosen because it is safe and inexpensive, Supporting Information 4. However, when other common electrolysis solvents were tested, such as DMSO, DMF, DMA, and MeCN, the bifluoride anion (HF_2_^–^) was formed in addition to fluoride (F^–^). In THF, no fluoride was formed, as 100% selectivity for HF_2_^–^ was observed, albeit in a lower yield.

**Figure 3:**
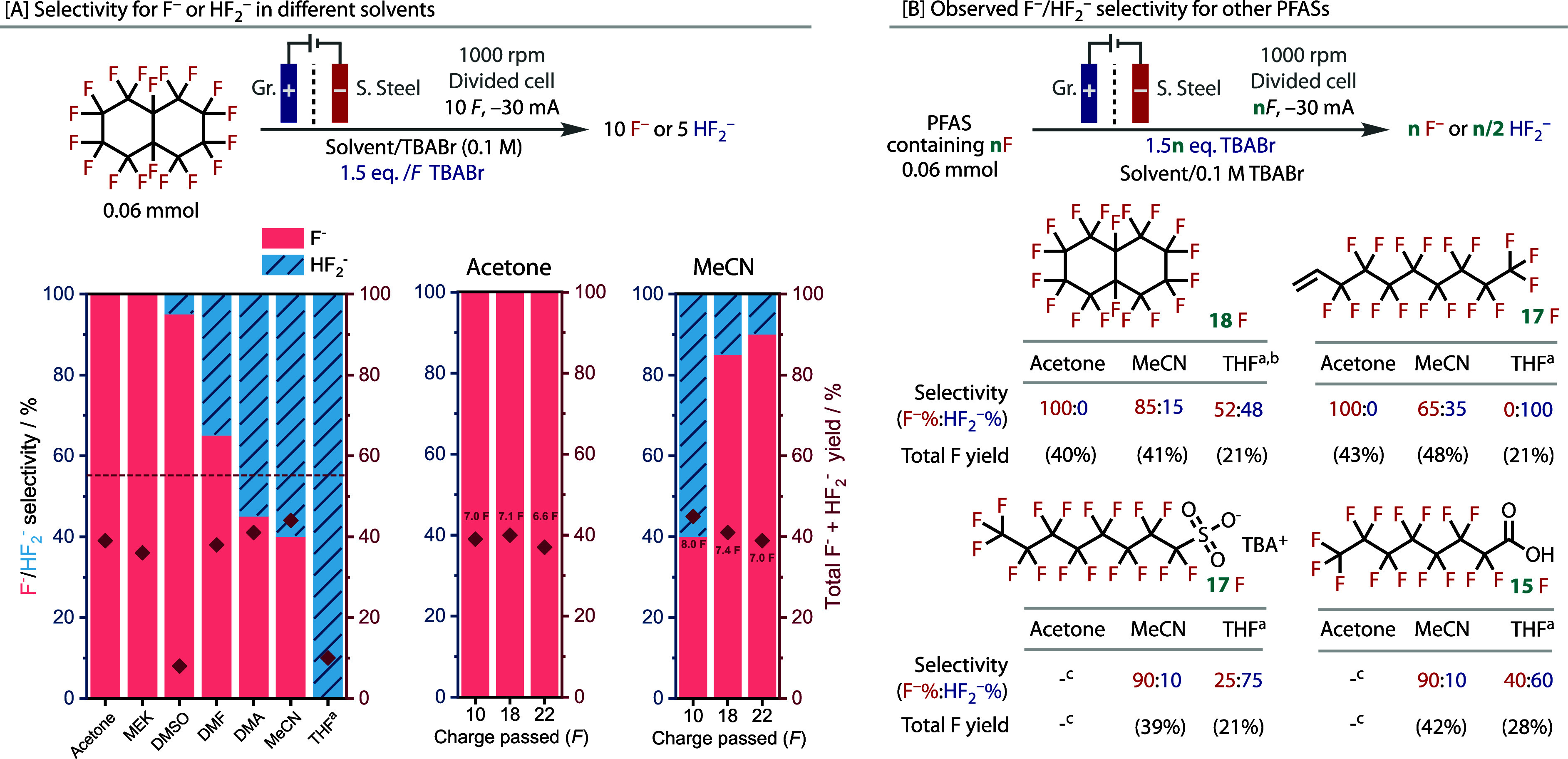
Selectivity for F^**–**^/HF_2_^**–**^ in different electrolytes. (A) Selectivity for fluoride vs bifluoride in various solvents when PFD is electrolyzed in a divided cell. The F^–^:HF_2_^–^ ratio and total defluorination yields were obtained by ^19^F NMR. −30 mA applied current is ca. 18.8 mA.cm^–2^. See Supporting Information 6 for values of *E*_cell_ obtained in each solvent. (B) F^–^:HF_2_^–^ ratio and total defluorination yields (^19^F NMR) for different PFAS in acetone, MeCN, and THF with *n F* (*n* = the number of fluorines per PFAS). ^a^–10 mA and 0.2 M TBABr were used as supporting electrolyte salt, rather than 0.1 M TBABr (the additional 1.5 equiv/*F* was still added in the anolyte), to decrease *E*_cell_. ^b^Average of 2 runs, see Figures S8.26, 27. ^c^Electrolyses of these PFAS were not successful in acetone.

The selectivity was assessed in acetone, MeCN, and THF when the amount of charge applied was varied. These solvents showed the best selectivity for fluoride, total yield of both fluoride and bifluoride, and selectivity for bifluoride, respectively. In acetone, the complete selectivity for F^–^ did not change with the amount of charge passed; however, in MeCN the preference for HF_2_^–^ decreased with more charge, Figure 3A. In THF, the low conductivity of the medium led to inconsistent yields and inconclusive results when more charge was passed; see Figures S8.26–28 for details.

The F^–^/HF_2_^–^ selectivity was assessed for a range of PFAS in acetone, MeCN, and THF, Figure 3B, when passing *n F* (*n* = the number of fluorine atoms in each PFAS). PFD and perfluoro-1-decene maintained 100% selectivity for F^–^ in acetone in good yield, although PFOA and PFOS electrolyses were surprisingly ineffective in acetone, as complete retention of starting material was observed, Figures S8.32 and S8.35. In MeCN, a mixed ratio in favor of fluoride for each PFAS was found but with the highest total fluorine yield of each solvent, while in THF, the selectivity for bifluoride generally remained but with a lower total fluoride yield.

Our hypothesis to rationalize the fluoride/bifluoride selectivity in different solvents is based on the different reactivities of fluoride that are produced in the different media. When the fluoride produced from the dissociative electron transfer is strongly basic, β-deprotonation of TBA^+^ salts via Hofmann elimination may occur to reveal the stable bifluoride anion HF_2_^–^.^46,47^ In contrast, when the reactivity of fluoride is dampened through solvation by water or solvent,^48,49^ it should remain as fluoride in solution. To test this hypothesis, control reactions were undertaken in THF, where the quantity of water was systematically varied, and Karl Fischer titrations were undertaken of each electrolyte used, including different supporting electrolyte salts, Supporting Information 5. These studies showed that bifluoride is formed in drier electrolytes, supporting the formation of a more basic fluoride anion, whereas no bifluoride was formed in wetter electrolytes, where solvation of fluoride dampens its reactivity. These studies also showed that the solvent also plays a role, as the sensitivity to water was found to be different in different solvents.

Building on these optimization studies, the isolation of metal fluoride reagents generated from the electrochemical decomposition of the PFD was subsequently targeted. Excellent selectivity for fluoride (F^–^) was observed using acetone as solvent in our divided cell conditions. Hence, we sought to exploit these conditions to extract high yields of fluoride. Isolation of the inorganic fluoride salts of Li, Na, and K was achieved by adding the respective, commercially available MPF_6_ salts. Aqueous extraction and freeze-drying delivered these alkali metal fluoride salts in good yields and very good to excellent purity, Figure 4. The identity of the salts was confirmed by powder X-ray diffraction (PXRD).

**Figure 4:**
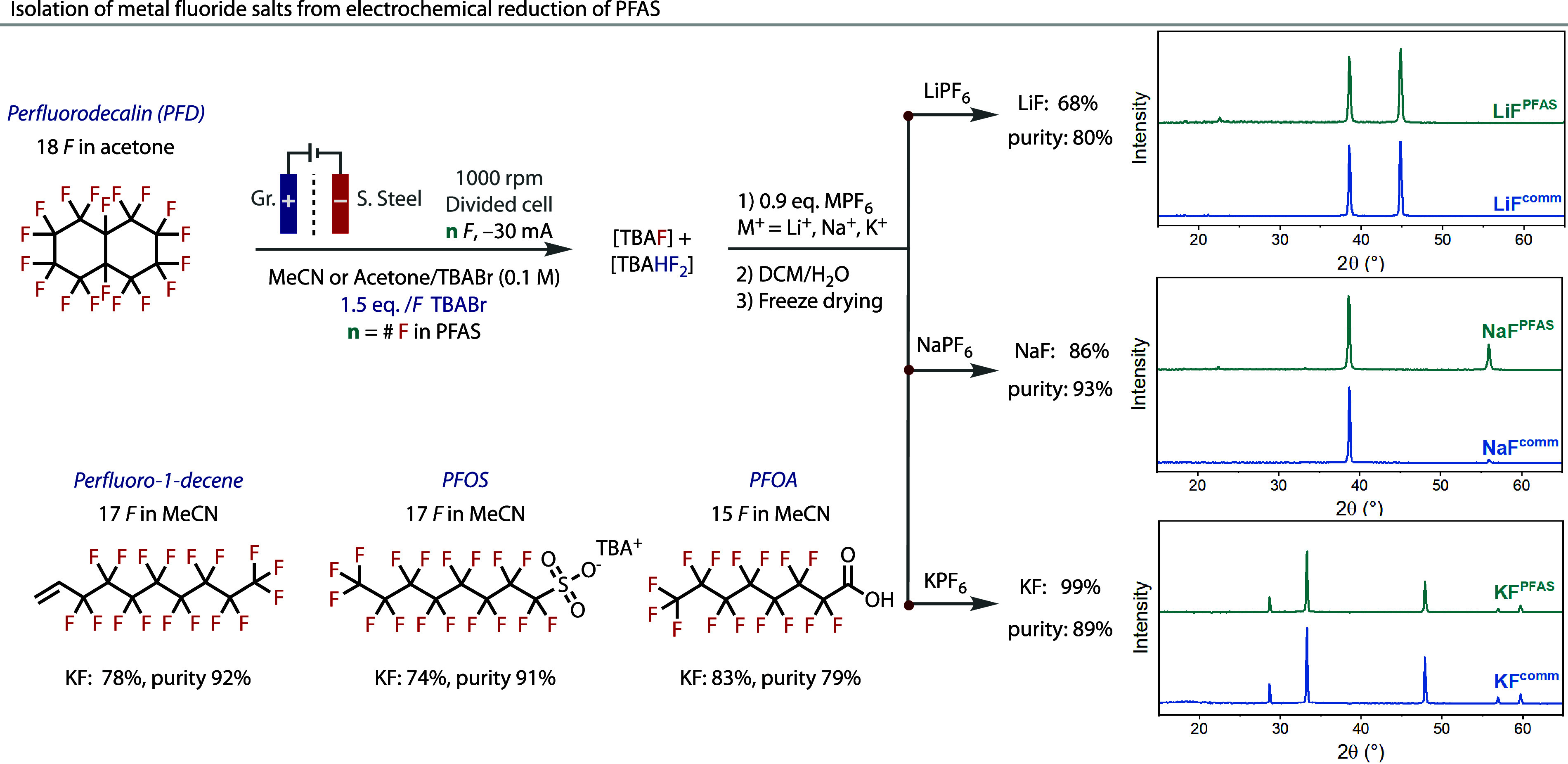
Synthesis and isolation of alkali metal fluoride salts. Electrochemical generation of fluoride, formally as a solution of TBAF and/or TBAHF_2_, from the divided cell electrolysis of PFAS (0.1 mmol scale) at ca. 18.8 mA·cm^–2^. The amount of alkali metal salt precursor added is relative to the quantity of electrogenerated F^–^, Figure 3B. Isolated yields are given relative to the limiting alkali metal salt precursor added. The purity of the as-synthesized salts was determined by quantitative ^19^F NMR; see Figures S8.52–57.

The use of other PFAS, including perfluoro-1-decene, PFOS, and PFOA, was tested as sources of fluoride from which KF could be isolated. As acetone was less successful in transforming other PFAS into fluoride, Figure 3B, we adopted the use of MeCN. Although MeCN generated quantities of bifluoride anion, Figure 3B, the aqueous workup was sufficient in converting it all to fluoride, and as such, high yields of KF were cleanly generated, Figure 4. These results demonstrate that our electrochemical methodology allows for the recycling of PFAS, for the first time, into *multiple* isolated inorganic fluoride salts, thereby broadening the applicability of this concept.

To diversify the fluorinating reagents synthesized from PFAS recycling, we targeted the formation of tetrabutylammonium difluorotriphenylsilicate (TBA^+^Ph_3_SiF_2_^–^, TBAT). TBAT is a crystalline solid that is widely used as a dry, non-hygroscopic, nucleophilic fluoride source that is often used as a substitute for TBAF, but is a less nucleophilic and less basic source of fluoride,^50–52^ for example in the formation of C–F bonds,^53,54^ benzyne intermediates,^55,56^ anions from Si–X cleavage,^57,58^ and also deprotections.^59,60^ TBAT is also used as a phenylation reagent in palladium-catalyzed cross-coupling reactions.^61^ Hence, we considered it to be an important and valuable target reagent to make from PFAS, which has not been previously achieved.

Our strategy evolved from our observations of the selective generation of bifluoride (HF_2_^–^) in solvents such as MeCN or THF. While TBAT can be made in a two-step reaction of Ph_3_SiOH with HF_(aq)_ in methanol to form Ph_3_SiF, followed by reaction with TBAF,^50,61^ it can also be formed from triphenylsilane (Ph_3_SiH) with TBAHF_2_.^62^ Hence, we proposed that we could optimize a procedure that combined our formation of HF_2_^–^ ions from PFAS, which uses the bromide-mediated silane oxidation conditions, but instead of using TESH to form TESF, we could use Ph_3_SiH to form TBAT.

Reaction optimization was initiated in an undivided cell, but the sensitivity of TBAT to acids was likely responsible for a lack of success with this strategy, Supporting Information 7.^51,61^ Hence, efforts turned to the use of a divided cell. Following reaction optimization, TBAT could be obtained with 90% Faradaic efficiency with MeCN/TBABr as electrolyte, which is a 50% yield of TBAT from a maximum of 56% when applying only 10 *F* during the optimization, Figure 5A, entry 1. When triphenylsilane became equimolar, the Faradaic efficiency reduced to 80%; hence, a small excess serves the reaction better (entry 2). When the wetter perchlorate supporting electrolyte was used, the yield declined further (entry 3). Importantly, when acetone was used as solvent, only F^–^ was formed, rather than HF_2_^–^, and consequently, only trace TBAT was observed (entry 4). This result highlights the importance of the choice of solvent for the efficient formation of bifluoride anions to react with Ph_3_SiH to form TBAT. Increasing the equivalents of charge (*F*) passed shows that the maximum number of fluorine atoms in PFD that can be harnessed and converted to TBAT is ca. 9, Supporting Information 7. The reaction did not proceed without electricity (entry 5).

**Figure 5:**
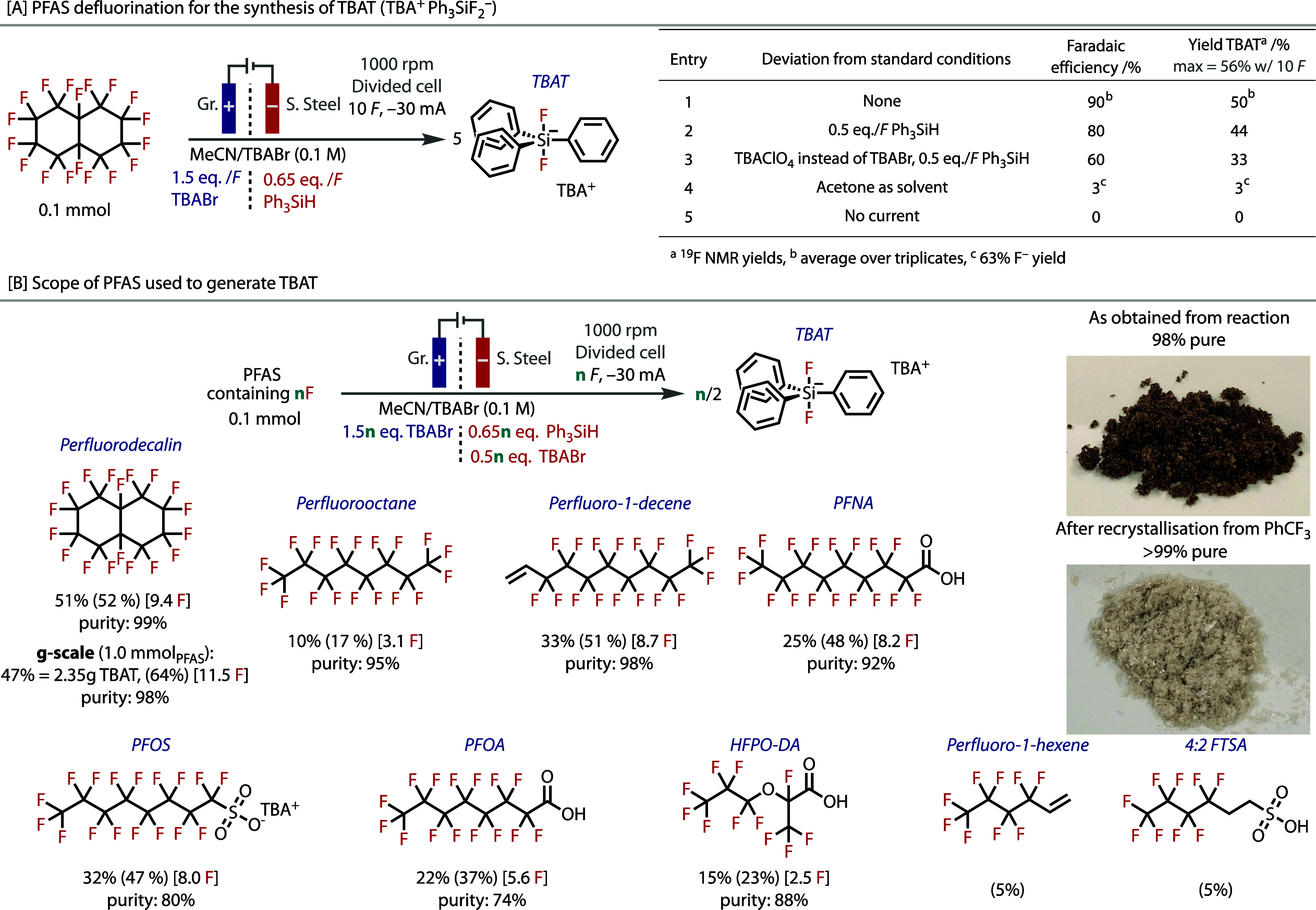
Synthesis and isolation of TBAT. (A) Optimization of TBAT formation from PFD. (B) PFAS substrate scope for TBAT synthesis in divided cells at −30 mA (ca. 18.8 mA·cm^−2^). Isolated yields are given and ^19^F NMR yields are given in parentheses. 0.5*n* equiv TBABr is used in the catholyte to ensure there are sufficient TBA^+^ cations for the electrogenerated Ph_3_SiF_2_^–^ anions. Electrolyses typically took ca. 50 min to ca. 1 h 40 min depending on the number of fluorines present in the PFAS. For the gram-scale synthesis from PFD, 1.0 mmol of PFD was used, which led to a reaction time of 16 h 05 min at −30 mA. *E*_cell_ was ca. 9–10 V, irrespective of the PFAS. Pictures of TBAT crystals obtained from the gram-scale synthesis from PFD and after subsequent recrystallization.

The reaction conditions were tested on an extended scope of PFAS, in which *n F* were applied to remove the *n* fluorine atoms present per PFAS. The reaction was found to work well on the range of PFAS that were tested, Figure 5B, although the shorter chain perfluoro-1-hexene and 4:2 FTSA were less well accommodated under these conditions. In each case, TBAT was isolated as orange to dark brown crystals in very good to excellent purity (74–99%, as determined by quantitative ^19^F NMR, Supporting Information 7 and Figures S8.68–74), depending on the PFAS. However, we found that the isolated TBAT could be readily recrystallized from PhCF_3_ to obtain analytically pure (>99%, ^19^F, ^1^H, ^13^C, and ^29^Si NMR) white crystals, Figure 5B and Figure S8.75–79. Importantly, the reaction was found to scale up easily, as 2.4 g of pure TBAT was obtained from 1.0 mmol of PFD, as shown in Figure 5B.

## Conclusion

In summary, we have developed a direct electrochemical strategy to synthesize a range of synthetically important fluoride reagents from the recycling of per- and polyfluoroalkyl substances (PFAS). The solvent plays a decisive role in dictating whether fluoride or bifluoride ions are generated when PFAS are electrolyzed. Acetone affords exclusive formation of fluoride, while MeCN produces a mixture of fluoride and bifluoride. This solvent control enabled the optimization of conditions for the isolation and purification of widely used metal fluoride reagents, including KF, NaF, and LiF. The generation of bifluoride in MeCN was exploited to access the non-hygroscopic fluoride source TBAT, a transformation that was not possible in acetone, where fluoride alone was formed. Collectively, this work establishes an electrochemical strategy for the decomposition of concentrated PFAS streams while simultaneously valorizing the liberated fluoride into useful fluorinating reagents, thereby providing a closed-loop approach to PFAS remediation and recovery of fluorine.

## Supplementary Material



## Data Availability

The experimental procedures and analytical data supporting the findings of the study are available in the paper and Supporting Information.

## Notes

Experimental section, additional characterization data, cyclic voltammetry, NMR spectra (PDF)

## References

[1] Glüge J., Scheringer M., Cousins I. T., Dewitt J. C., Goldenman G., Herzke D., Lohmann R., Ng C. A., Trier X., Wang Z. (2020). An Overview of the Uses of Per- And Polyfluoroalkyl Substances (PFAS). Environ. Sci. Process. Impacts.

[2] Sunderland E. M., Hu X. C., Dassuncao C., Tokranov A. K., Wagner C. C., Allen J. G. (2019). A Review of the
Pathways of Human Exposure to Poly-
and Perfluoroalkyl Substances (PFASs) and Present Understanding of
Health Effects. J. Expo. Sci. Environ. Epidemiol..

[3] Brunn H., Arnold G., Körner W., Rippen G., Steinhäuser K. G., Valentin I. (2023). PFAS: Forever Chemicals—Persistent, Bioaccumulative
and Mobile. Reviewing the Status and the Need for Their Phase out
and Remediation of Contaminated Sites. Environ.
Sci. Eur..

[4] Vecitis C. D., Park H., Cheng J., Mader B. T., Hoffmann M. R. (2008). Kinetics
and Mechanism of the Sonolytic Conversion of the Aqueous Perfluorinated
Surfactants, Perfiuorooctanoate (PFOA), and Perfluorooctane Sulfonate
(PFOS) into Inorganic Products. Journal of Physical
Chemistry A.

[5] Cheng J., Vecitis C. D., Park H., Mader B. T., Hoffmann M. R. (2010). Sonochemical
Degradation of Perfluorooctane Sulfonate (PFOS) and Perfluorooctanoate
(PFOA) in Groundwater: Kinetic Effects of Matrix Inorganics. Environ. Sci. Technol..

[6] Yin S., Calvillo Solís J. J., Sandoval-Pauker C., Puerto-Diaz D., Villagrán D. (2025). Advances in
PFAS Electrochemical
Reduction: Mechanisms, Materials, and Future Perspectives. J. Hazard. Mater..

[7] King J. F., Chaplin B. P. (2024). Electrochemical Reduction of Per-
and Polyfluorinated
Alkyl Substances (PFAS): Is It Possible? Applying Experimental and
Quantum Mechanical Insights from the Reductive Defluorination Literature. Curr. Opin. Chem. Eng..

[8] Trautmann A. M., Schell H., Schmidt K. R., Mangold K. M., Tiehm A. (2015). Electrochemical
Degradation of Perfluoroalkyl and Polyfluoroalkyl Substances (PFASs)
in Groundwater. Water Sci. Technol..

[9] Schaefer C. E., Andaya C., Urtiaga A., McKenzie E. R., Higgins C. P. (2015). Electrochemical
Treatment of Perfluorooctanoic Acid (PFOA) and Perfluorooctane Sulfonic
Acid (PFOS) in Groundwater Impacted by Aqueous Film Forming Foams
(AFFFs). J. Hazard. Mater..

[10] Amerio-Cox M., Tully J. J., Tang F., Dettlaff A., Macpherson J. V., Mollart T., Sidnell T., Mathias S., Sears P., Bussemaker M. J. (2025). Investigation
of Short Chain PFAS Degradation Efficiency
Using Free-Standing Boron Doped Diamond Electrodes at High Current
Density in a Flow Cell. ACS Electrochemistry.

[11] Hori H., Hayakawa E., Einaga H., Kutsuna S., Koike K., Ibusuki T., Kiatagawa H., Arakawa R. (2004). Decomposition of Environmentally
Persistent Perfluorooctanoic Acid in Water by Photochemical Approaches. Environ. Sci. Technol..

[12] Hori H., Yamamoto A., Hayakawa E., Taniyasu S., Yamashita N., Kutsuna S., Kiatagawa H., Arakawa R. (2005). Efficient Decomposition
of Environmentally Persistent Perfluorocarboxylic Acids by Use of
Persulfate as a Photochemical Oxidant. Environ.
Sci. Technol..

[13] Liang X., Cheng J., Yang C., Yang S. (2016). Factors Influencing
Aqueous Perfluorooctanoic Acid (PFOA) Photodecomposition by VUV Irradiation
in the Presence of Ferric Ions. Chemical Engineering
Journal.

[14] Bentel M. J., Yu Y., Xu L., Li Z., Wong B. M., Men Y., Liu J. (2019). Defluorination of Per- and Polyfluoroalkyl Substances (PFASs) with
Hydrated Electrons: Structural Dependence and Implications to PFAS
Remediation and Management. Environ. Sci. Technol..

[15] Singh R. K., Fernando S., Baygi S. F., Multari N., Thagard S. M., Holsen T. M. (2019). Breakdown Products from Perfluorinated Alkyl Substances
(PFAS) Degradation in a Plasma-Based Water Treatment Process. Environ. Sci. Technol..

[16] Liu Z., Bentel M. J., Yu Y., Ren C., Gao J., Pulikkal V. F., Sun M., Men Y., Liu J. (2021). Near-Quantitative
Defluorination of Perfluorinated and Fluorotelomer Carboxylates and
Sulfonates with Integrated Oxidation and Reduction. Environ. Sci. Technol..

[17] Watanabe N., Takemine S., Yamamoto K., Haga Y., Takata M. (2016). Residual Organic
Fluorinated Compounds from Thermal Treatment of PFOA, PFHxA and PFOS
Adsorbed onto Granular Activated Carbon (GAC). J. Mater. Cycles Waste Manag..

[18] Xiao F., Sasi P. C., Yao B., Kubátová A., Golovko S. A., Golovko M. Y., Soli D. (2020). Thermal Stability and
Decomposition of Perfluoroalkyl Substances on Spent Granular Activated
Carbon. Environ. Sci. Technol. Lett..

[19] Farley S., Crimmin M. (2025). Synthetic Methodologies for the Chemical Recycling
of Fluorocarbons. ChemRxiv..

[20] Trang B., Li Y., Xue X.-S., Ateia M., Houk K. N., Dichtel W. R. (2022). Low-Temperature
Mineralization of Perfluorocarboxylic Acids. Science..

[21] Bui M., Heinekamp C., Fuhry E., Weidner S., Radnik J., Ahrens M., Scheurell K., Balasubramanian K., Emmerling F., Braun T. (2025). Lewis-Acid Induced Mechanochemical
Degradation of Polyvinylidene Fluoride: Transformation into Valuable
Products. Chem. Sci..

[22] Sheldon D. J., Parr J. M., Crimmin M. R. (2023). Room Temperature
Defluorination of
Poly­(Tetrafluoroethylene) by a Magnesium Reagent. J. Am. Chem. Soc..

[23] Zhang H., Chen J.-X., Qu J.-P., Kang Y.-B. (2024). Photocatalytic Low-Temperature
Defluorination of PFASs. Nature.

[24] Fu J., Liu Y., Chen Y., Zhang H., Qu J., Kang Y. (2025). Electrophotocatalysis
for Reductive Defluorination of PTFE and PFASs. Angew. Chem., Int. Ed..

[25] Scott V. J., Çelenligil-Çetin R., Ozerov O. V. (2005). Room-Temperature
Catalytic Hydrodefluorination of C­(Sp3)-F Bonds. J. Am. Chem. Soc..

[26] Douvris C., Ozerov O. V. (2008). Hydrodefluorination
of Perfluoroalkyl Groups Using
Silylium-Carborane Catalysts. Science..

[27] Liu X., Sau A., Green A. R., Popescu M. V., Pompetti N. F., Li Y., Zhao Y., Paton R. S., Damrauer N. H., Miyake G. M. (2025). Photocatalytic
C–F Bond Activation in Small Molecules and Polyfluoroalkyl
Substances. Nature.

[28] Harsanyi A., Sandford G. (2015). Organofluorine Chemistry:
Applications, Sources and
Sustainability. Green Chem..

[29] European Commission. Critical Raw Materials Resilience: Charting a Path towards Greater Security and Sustainability. https://ec.europa.eu/docsroom/documents/42849 (accessed 2025–04–08).

[30] Gillis E. P., Eastman K. J., Hill M. D., Donnelly D. J., Meanwell N. A. (2015). Applications
of Fluorine in Medicinal Chemistry. J. Med.
Chem..

[31] Long H., Kirby G., Ackermann L. (2025). Single-Pot
Mechanochemically-Enabled
Fluorine Atom Closed- Loop Economy Using PFASs as Fluorinating Agents. ChemRxiv..

[32] Hattori M., Kiyono T., Zhao Z., Higashi M., Fujishiro M., Kishikawa Y., Escorihuela J., Shibata N. (2026). Upcycling of PTFE and
PVDF to Fluorochemicals through Mechanochemical Process. Nat. Commun..

[33] Jenek, N. ; Brock nee Patrick, S. ; Mao, J. ; Fogh, A. ; Crimmin, M. Chemical Recycling of Hydrofluorocarbons by Transfer Fluorination. October 28 ChemRxiv 2025, 10.26434/chemrxiv-2025-frf8j-v2 (accessed 2025–10–30). PMC13149033 41826676

[34] Bennett B. K., Harrison R. G., Richmond T. G. (1994). Cobaltocenium Fluoride: A Novel Source
of “Naked” Fluoride Formed by Carbon-Fluorine Bond Activation
in a Saturated Perfluorocarbon. J. Am. Chem.
Soc..

[35] Lowe M. E., Gallant B. M., Davison N., Hopkinson M. N., Kubicki D. J., Lu E., Armstrong R. J. (2025). A Reductive
Mechanochemical Approach Enabling Direct Upcycling of Fluoride from
Polytetrafluoroethylene (PTFE) into Fine Chemicals. J. Am. Chem. Soc..

[36] Hattori M., Saha D., Bacho M. Z., Shibata N. (2025). Mechanochemical Pathway
for Converting Fluoropolymers to Fluorochemicals. Nat. Chem..

[37] Yang L., Chen Z., Goult C. A., Schlatzer T., Paton R. S., Gouverneur V. (2025). Phosphate-Enabled Mechanochemical
PFAS Destruction for Fluoride Reuse. Nature.

[38] Araki T., Ota H., Murata Y., Sumii Y., Hamaura J., Adachi H., Kagawa T., Hori H., Escorihuela J., Shibata N. (2025). Room-Temperature Defluorination of PTFE and PFAS via
Sodium Dispersion. Nat. Commun..

[39] Sarkar B., Kumawat R. L., Ma P., Wang K.-H., Mohebi M., Schatz G. C., Amanchukwu C. V. (2026). Lithium
Metal-Mediated Electrochemical
Reduction of per- and Poly-Fluoroalkyl Substances. Nat. Chem..

[40] Box J. R., Atkins A. P., Lennox A. J. J. (2021). Direct Electrochemical Hydrodefluorination
of Trifluoromethylketones Enabled by Non-Protic Conditions. Chem. Sci..

[41] Box J. R., Avanthay M. E., Poole D. L., Lennox A. J. J. (2023). Electronically
Ambivalent Hydrodefluorination of Aryl-CF _3_ Groups Enabled
by Electrochemical Deep-Reduction on a Ni Cathode. Angew. Chem., Int. Ed..

[42] Avanthay M. E., Goodrich O. H., Tiemessen D., Alder C. M., George M. W., Lennox A. J. J. (2024). Bromide-Mediated Silane Oxidation: A Practical Counter-Electrode
Process for Nonaqueous Deep Reductive Electrosynthesis. JACS Au.

[43] Figueira R. B. (2020). Hybrid
Sol–Gel Coatings for Corrosion Mitigation: A Critical Review. Polymers (Basel)..

[44] Shine K. P., Gohar L. K., Hurley M. D., Marston G., Martin D., Simmonds P. G., Wallington T. J., Watkins M. (2005). Perfluorodecalin: Global
Warming Potential and First Detection in the Atmosphere. Atmos. Environ..

[45] Combellas C., Kanoufi F., Thiébault A. (2003). Reduction
of Polyfluorinated Compounds. J. Phys. Chem.
B.

[46] Sun H., DiMagno S. G. (2005). Anhydrous Tetrabutylammonium Fluoride. J. Am. Chem. Soc..

[47] Sharma R. K., Fry J. L. (1983). Instability of Anhydrous
Tetra-n -Alkylammonium Fluorides. J. Org. Chem..

[48] Landini D., Maia A., Rampoldi A. (1989). Dramatic Effect of the Specific Solvation
on the Reactivity of Quaternary Ammonium Fluorides and Poly­(Hydrogen
Fluorides), (HF)­n.Cntdot.F-, in Media of Low Polarity. J. Org. Chem..

[49] Sweeting S. G., Lennox A. J. J. (2025). Non-Aqueous Binary
and Ternary NHF·Base Fluoride
Reagents: Characterization of Structure, Properties, and Reactivity. J. Am. Chem. Soc..

[50] Pilcher A. S., Ammon H. L., DeShong P. (1995). Utilization
of Tetrabutylammonium
Triphenylsilyldifluoride as a Fluoride Source for Nucleophilic Fluorination. J. Am. Chem. Soc..

[51] Simpkins, N. S. ; Nytko, F. E. ; DeShong, P. ; Vayer, M. ; Maulide, N. Tetrabutylammonium Difluorotriphenylsilicate (TBAT). In Encyclopedia of Reagents for Organic Synthesis; Wiley, 2020; pp 1–14 10.1002/047084289X.rn00469.pub3.

[52] Kucharski M. M., Watson A. J. B., Lloyd-Jones G. C. (2024). Speciation
and Kinetics of Fluoride
Transfer from Tetra-n-Butylammonium Difluorotriphenylsilicate (‘TBAT’). Chem. Sci..

[53] Gamache R. F., Waldmann C., Murphy J. M. (2016). Copper-Mediated
Oxidative Fluorination
of Aryl Stannanes with Fluoride. Org. Lett..

[54] Adler P., Teskey C. J., Kaiser D., Holy M., Sitte H. H., Maulide N. (2019). α-Fluorination of Carbonyls with Nucleophilic
Fluorine. Nat. Chem..

[55] Donnelly K., Baumann M. (2022). Continuous Flow Technology
as an Enabler for Innovative
Transformations Exploiting Carbenes, Nitrenes, and Benzynes. J. Org. Chem..

[56] Wang Q., An Y., Du G., Cai Z. H., Dai B., He L. (2020). Direct Assembly
of Polysubstituted Naphthalenes via a Tandem Reaction of Benzynes
and α-Cyano-β-Methylenones. J. Org.
Chem..

[57] Keereewan S., Kuhakarn C., Leowanawat P., Saithong S., Reutrakul V., Soorukram D. (2022). Diastereoselective Addition of PhSCF2SiMe3to Chiral
N- Tert-Butanesulfinyl Ketimines Derived from Isatins: Synthesis of
Enantioenriched Gem-Difluoromethylenated Spiro-Pyrrolidinyl and Spiro-Piperidinyl
Oxindoles. J. Org. Chem..

[58] Cai Y., Zhu W., Zhao S., Dong C., Xu Z., Zhao Y. (2021). Difluorocarbene-Mediated
Cascade Cyclization: The Multifunctional Role of Ruppert-Prakash Reagent. Org. Lett..

[59] Killen J. C., Axford L. C., Newberry S. E., Simpson T. J., Willis C. L. (2012). Convergent
Syntheses of 3,6-Dihydroxydec-4-Enolides. Org.
Lett..

[60] Campbell M. J., Pohlhaus P. D., Min G., Ohmatsu K., Johnson J. S. (2008). An “Anti-Baldwin”
3-Exo-Dig Cyclization: Preparation of Vinylidene Cyclopropanes from
Electron-Poor Alkenes. J. Am. Chem. Soc..

[61] Handy C. J., Lam Y., DeShong P. (2000). On the Synthesis
and NMR Analysis of Tetrabutylammonium
Triphenyldifluorosilicate. J. Org. Chem..

[62] Albanese D., Landini D., Penso M. (1995). Synthesis
of Pentacoordinated Tetraalkylammonium
and Tetraalkylphosphonium Difluorosilicates. Tetrahedron Lett..

