# Supplementary material for: Electrochemical Synthesis of Isolated Fluoride Reagents from PFAS
Source: J Am Chem Soc. 2026 Mar 9;148(11):12255–63. doi: 10.1021/jacs.6c01373 (PMC13022864; doi:10.1021/jacs.6c01373)
Supplement: Supplementary file 1 [file ja6c01373_si_001.pdf]

Supporting Information

Electrochemical Synthesis of Isolated Fluoride Reagents from PFAS

Florian Dorchies, Luke P. Ward, Isaac P. Richards, Mohamed Elsherbini and Alastair J. J. Lennox*

School of Chemistry, University of Bristol, Cantock's Close, Bristol, Avon BS8 1TS, U.K.

*Correspondence to a.lennox@bristol.ac.uk

Table of Contents

General experimental **3**

1. Synthesis of PFOS-TBA **7**

2. Cyclic voltammetry experiments **9**

a. Cyclic voltammetry of the PFAS investigated 9

b. Cyclic voltammetry of other compounds used in the study 14

3. Synthesis of NaF and KF from TESF **16**

4. Divided cell electrolysis in acetone – effect of the cathode material **18**

5. F^-/HF_2^- selectivity – Effect of the presence of water in the electrolysis medium **19**

6. Divided cell electrolysis of PFD – E_{cell} values in different solvents **21**

7. TBAT synthesis in (un)divided cells **22**

a. Attempts to synthesize TBAT in undivided cells 22

b. TBAT yield as a function of the charge (*F*) passed in divided cells 23

c. Solid that precipitates after divided cell electrolysis for TBAT synthesis 24

8. NMR spectra **29**

a. Undivided cell electrolysis – TESF synthesis 29

b. Divided cell electrolysis of PFAS – F^-/HF_2^- selectivity 41

c. Divided cell electrolysis of PFAS – Isolation of fluoride salts 80

d. Divided cell electrolysis – TBAT synthesis 86

9. Electrolysis setups **108**

Supplementary References **112**

General experimental

Solvents. Deuterated water, deuterated chloroform and deuterated acetonitrile were purchased from Sigma-Aldrich. Deuterated acetonitrile was dried over molecular sieves. Acetonitrile, diethyl ether and tetrahydrofuran were dried using an anhydrous engineering alumina column drying system situated in the University of Bristol's chemistry department and collected in Strauss flasks equipped with a gastight J Young valve. Dimethyl sulfoxide (≥99.9%, Fisher Scientific), N,N-dimethylacetamide (anhydrous, 99.8%, Sigma-Aldrich), N,N-dimethylformamide (99+%, extra pure, Fisher Scientific) and α,α,α-trifluorotoluene (anhydrous, ≥99%, Sigma-Aldrich) were dried over molecular sieves and stored in Strauss flasks. Acetone (puriss. p.a., ACS reagent, reag. ISO, reag. Ph. Eur., ≥99.5%, Sigma-Aldrich) and methyl ethyl ketone (≥99%, Fisher Scientific) were dried over molecular sieves in a Schlenk flask an hour before each electrolysis.

Reagents. Fluorobenzene (99%), 4-fluorobenzenesulfonyl chloride (98%), 1H,1H,2H-perfluoro-1-decene (99%), 1H,1H,2H-perfluoro-1-hexene (99%), perfluoronaphthalene (96%), perfluorononanoic acid (97%), perfluorooctane (98%), perfluorooctanoic acid (95%), potassium tert-butoxide (≥98%), tetrabutylammonium perchlorate (≥95%), tetramethylammonium fluoride (97%), triethylsilane (99%), triphenylsilane (97%) were purchased from Sigma-Aldrich. Tetrabutylammonium bromide (≥99.0%) was purchased from Sigma-Aldrich and recrystallized in ethyl acetate and dried in a vacuum oven before being introduced in a N_2-filled glovebox. Perfluorodecalin (95%) was purchased from Sigma-Aldrich and distilled before being introduced in a N_2-filled glovebox. Cesium fluoride was purchased from Thermo Scientific. Sodium triflate and $LiPF_6$ were purchased from Fluorochem. KPF_6 (99%, extra pure), $NaPF_6$ (98.5+%) and sodium tert-butoxide (99%) were purchased from Acros Organics. 1H,1H,2H,2H-perfluorohexanesulfonic acid (4:2 FTSA) was purchased from Apollo Scientific. Hexafluoropropylene oxide dimer acid (95%) was purchased from Enamine. Perfluorooctanesulfonic acid, potassium salt (95%) was purchased from abcr GmbH. Tetrabutylammonium difluorotriphenylsilicate (98%) was purchased from Combi-Blocks. PFOS-TBA was synthesized from PFOS-K, **Supporting Information 1**.

Cyclic Voltammetry. Data was acquired on an Autolab/M101 potentiostat. All measurements were recorded using a three-electrode cell setup with a homemade $Ag/AgNO_3$ (0.01 M $AgNO_3$ in MeCN/0.1 M $TBAPF_6$) reference electrode. A graphite rod was used as the counter electrode. Prior to any measurement, the glassy carbon working electrode (3 mm diameter,

0.0707 cm^2 geometric surface area) was polished with a polishing slurry (0.3 μm aluminum oxide) on a microcloth polishing disc. Residual traces of slurry were removed by sonicating the as-polished electrode three times in water (1 min each) and one time in acetone (1 min). All measurements were conducted in a Schlenk flask under dry and inert N_2 atmosphere. Prior to any measurements, a Schlenk flask was introduced in a N_2-filled glovebox, the substrate and supporting salt added and the flask capped with a Suba-Seal septum. The flask was then taken out of the glovebox, connected to a Schlenk line, and dry solvent was added under positive N_2 pressure. The three electrodes were then added under high N_2 pressure. The ohmic drop was not compensated. Data was externally referenced against ferrocenium/ferrocene.

Electrolysis. Constant current was applied with an AimTTi PLH250 or PLH120 DC power supply. Ni, Zn, stainless steel and graphite plate electrodes (5.2*0.8*0.2 cm^3) were purchased from IKA. Prior to electrolysis, the working electrode was polished with sandpaper. The graphite anode was cleaned by soaking it in acetone and subsequently dried. The surface area of the anode and cathode immersed in the electrolytes was ~ 1.6 cm^2 for both undivided and divided cell conditions. Applied currents of -10, -30 and -50 mA thus correspond to current densities of ~ 6.3, ~ 18.8 and ~ 31.5 $mA.cm^{-2}$, respectively. After each electrolysis, the catholyte (for divided cell conditions) or the electrolyte (for undivided cell conditions) was collected in a plastic falcon tube with a plastic pipette.

<u>Undivided cell electrolyses:</u> All undivided cell electrolyses were conducted in a glass Schlenk flask (see **Figures S9.1-3**) under dry and inert N_2 atmosphere. The flask was introduced in a N_2-filled glovebox, the PFAS and supporting salt added and the flask capped with a Suba-Seal septum. The flask was then taken out of the glovebox, connected to a Schlenk line, and dry solvent was added under positive N_2 pressure. TESH was then added under positive N_2 pressure and the electrodes added under high N_2 pressure. 6 mL of solvent was used for electrolyses at 0.06 and 0.1 mmol PFAS scale and 30 mL for the gram-scale synthesis. Undivided cell electrolyses conducted at -30 mA at a 0.1 mmol PFAS scale for TESF synthesis with 2*n F* (*n* being the number of fluorine in the PFAS) took ~ 1h40 min to ~ 3h15 depending on the number of fluorine present in the PFAS. For the TESF scope, E_{cell} was ~ 4-5V, irrespective of the PFAS. The gram-scale TESF synthesis conducted with 1.0 mmol PFD at -50 mA took 19h18min.

<u>Divided cell electrolyses:</u> All divided cell electrolyses were conducted in a glass divided cell (see **Figures S9.4-6**) under dry and inert N_2 atmosphere. The electrochemical cell was introduced in a N_2-filled glovebox, the substrates and supporting salt added, the graphite anode

introduced in the anolyte compartment and the cell capped with Suba-Seal septums. The cell was then taken out of the glovebox, connected to a Schlenk line, and dry solvent was added under positive N_2 pressure. The cathode was then added under high N_2 pressure. 5 mL of solvent was used in each compartment for electrolyses at 0.06 and 0.1 mmol PFAS scale and 20 mL in each compartment for the gram-scale synthesis. Divided cell electrolyses conducted at -30 mA at a 0.06 mmol PFAS scale with *n F* (*n* being the number of fluorine in the PFAS) took ~ 30 min to ~ 1h depending on the number of fluorine present in the PFAS. Divided cell electrolyses conducted at -10 mA in THF at a 0.06 mmol PFAS scale with *n F* (*n* being the number of fluorine in the PFAS) took ~ 1h30 min to ~ 3h depending on the number of fluorine present in the PFAS. Divided cell electrolyses conducted at -30 mA at a 0.1 mmol PFAS scale for TBAT synthesis with *n F* (*n* being the number of fluorine in the PFAS) took ~ 50 min to ~ 1h40 depending on the number of fluorine present in the PFAS. For the TBAT scope, E_{cell} was ~ 9-10 V, irrespective of the PFAS. The gram-scale TBAT synthesis conducted with 1.0 mmol PFD at -30 mA took 16h05min.

Isolation of fluoride salts from PFAS. The catholyte from the divided cell electrolyses of PFAS in either acetone or MeCN was collected in a plastic falcon tube with a plastic pipette. Fluorobenzene was added to the tube to quantify the amount of F^-/HF_2^- generated. 0.9 equivalents of MPF_6 salt (M^+=Li^+, Na^+, K^+) were then added to the falcon tube followed by ~ 5mL of dichloromethane. Fluoride salts were then extracted in water (6 times 1 mL). The aqueous phase was then washed with dichloromethane and freeze dried overnight. The resulting brown/orange solid was washed two times with dry acetone and one time with diethyl ether and subsequently dried under high vacuum. White to cream white solids were obtained and their purity quantified by ^{19}F NMR.

Isolation of TBAT. The catholyte from the divided cell electrolyses of PFAS for TBAT synthesis was collected in a plastic falcon tube with a plastic pipette. The tube was centrifuged and the supernatant collected (process repeated as many times as needed) to remove 1,1,1,3,3,3-hexaphenyldisiloxane ($(Ph_3Si)_2O$) that precipitates upon exposure of the crude to air (see **section 7** of the SI for thorough information). Fluorobenzene was added to obtain a TBAT ^{19}F NMR yield. The crude was then concentrated to half and a seed of commercial TBAT was added. The tube was then left in the freezer for two days. Brown/orange crystals of TBAT were obtained in 74-99% purity (as determined by ^{19}F quantitative NMR – **Figures S8.68-74**) and filtered. TBAT crystals can be recrystallized in trifluorotoluene to obtain analytically pure crystals (**Figures S8.75-79**).

Karl Fischer titration. Karl Fischer titration was performed on a Hanna Instruments Karl Fischer coulometric titrator HI934 and each measurement was performed in triplicates. HYDRANAL - Coulomat AG (Honeywell) was used as reagent for measurements with acetonitrile and THF and HYDRANAL - Coulomat AK (Honeywell) was used as reagent for measurements with acetone.

Nuclear Magnetic Resonance Spectroscopy. NMR spectra were recorded on Varian 500, JEOL ECS 400, ECZ 400 or ECS 300 spectrometers. All chemical shifts were quoted in parts per million (ppm); ^{1}H and ^{13}C NMR spectra were referenced to TMS and ^{19}F to $CFCl_3$. The following abbreviations were used to label the multiplicities: s (singlet), d (doublet), t (triplet), and m (multiplet). For ^{19}F NMR yields and quantitative ^{19}F NMR, spectra were recorded using a 30 second recycle delay. Fluorobenzene was used as an internal standard for ^{19}F NMR yields for its stability in the presence of electrogenerated F^{-}/HF_2^{-} species. To determine the purity of $TBAT^{PFAS}$, quantitative ^{19}F NMR spectra were recorded in deuterated acetonitrile (dried over molecular sieves) with fluorobenzene as internal standard. To determine the purity of LiF^{PFAS}, NaF^{PFAS} and KF^{PFAS}, quantitative ^{19}F NMR spectra were recorded in deuterated water with sodium triflate as internal standard.

Powder X-ray Diffraction. Powder X-ray diffraction (PXRD) data was collected using a Bruker D8 Advance X-ray diffractometer in the Bragg−Brentano configuration with a non-monochromatic Cu Kα X-ray source (wavelength ~1.541 Å). Samples were mounted in an airtight holder with a domed plastic lid and a rubber O-ring. Patterns were acquired with a 2θ range of 15 to 65 degrees, 0.1 degree step and 1s per step.

1. Synthesis of PFOS-TBA

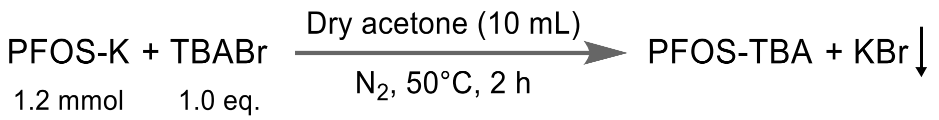

664 mg of PFOS-K (1.2 mmol) and 398 mg of TBABr (1.2 mmol, 1.0 eq.) were added to a plastic Falcon tube in a N_2-filled glovebox. The falcon tube was taken out of the glovebox and 10 mL of dry acetone were added. The mixture was heated at 50°C for 2h. A yellow solution with a white insoluble solid (KBr) was obtained. The white solid was removed by centrifugation and acetone removed under vacuum. The resulting orange viscous oil was dried under high vacuum. PFOS-TBA is an ionic liquid.[1]

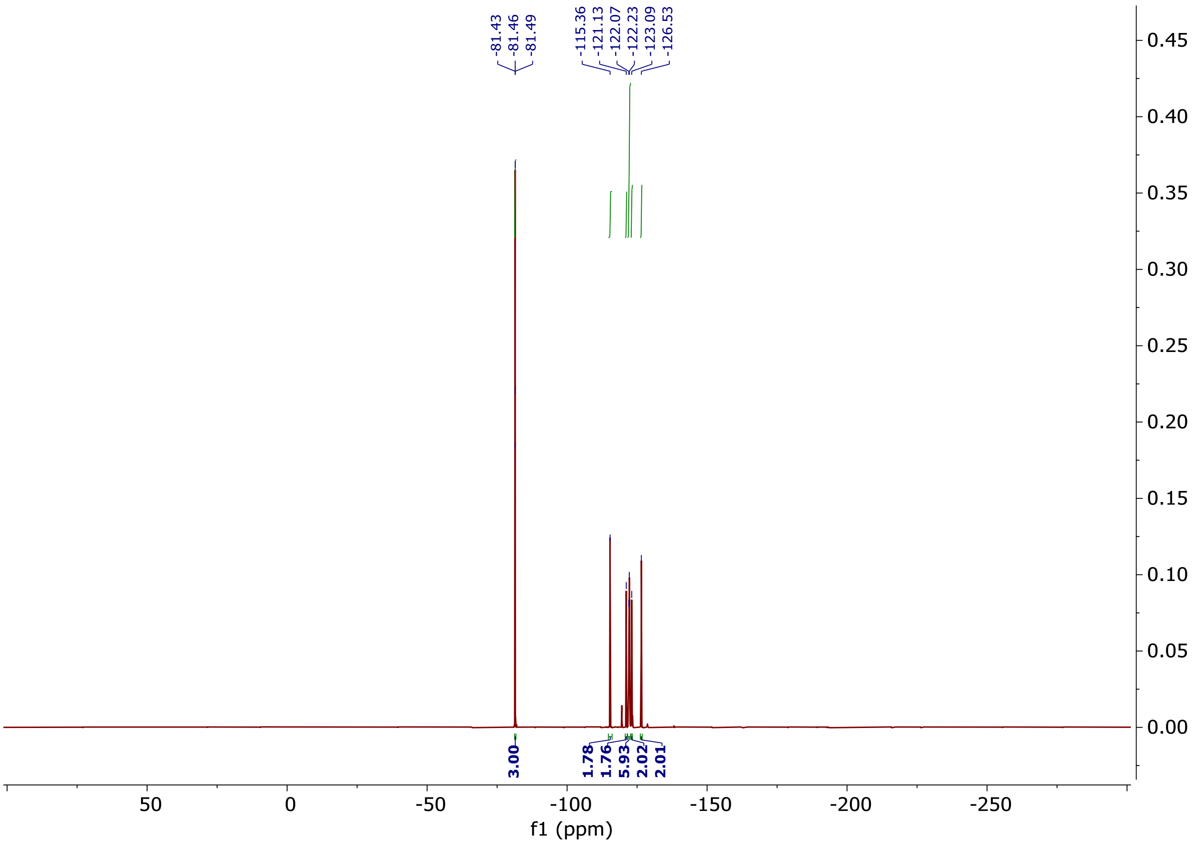

Figure S1.1. ^{19}F NMR (376 MHz, CD_3CN) spectrum of PFOS-TBA.

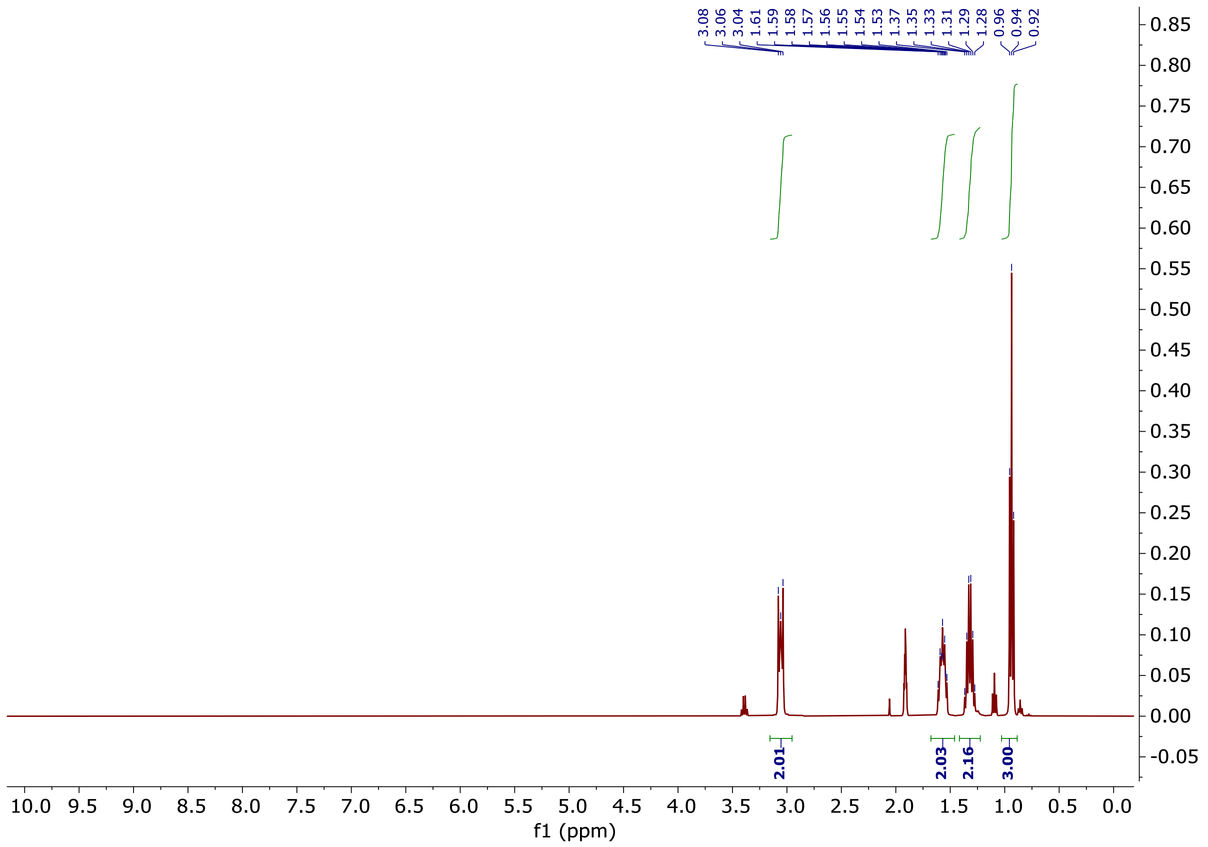

Figure S1.2. ^{1}H NMR (400 MHz, CD_3CN) spectrum of PFOS-TBA. Traces of diethyl ether (used to dry the compound) and water are visible alongside the residual NMR solvent peak.

2. Cyclic voltammetry experiments

a. Cyclic voltammetry of the PFAS investigated

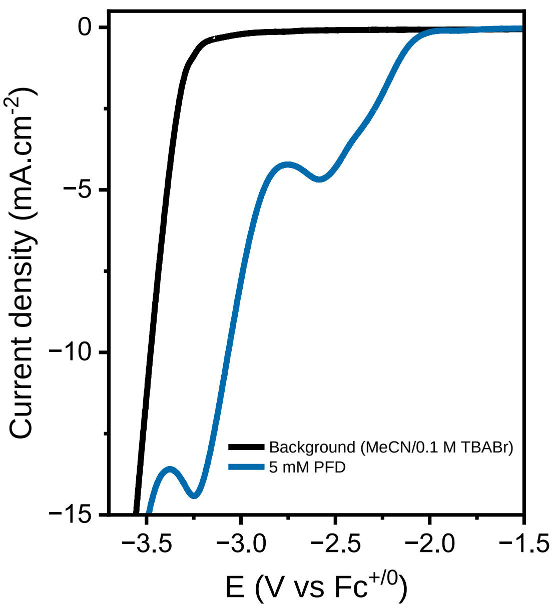

Figure S2.1. Cyclic voltammetry of PFD in MeCN in reduction. Working electrode: glassy carbon, counter electrode: graphite, Ref: Ag/$AgNO_3$ (0.01 M $AgNO_3$ in MeCN/0.1 M $TBAPF_6$), dry and N_2-saturated MeCN/0.1 M TBABr, 5.0 mM PFD, room temperature (~ 20°C), 100 mV.s^{-1}.

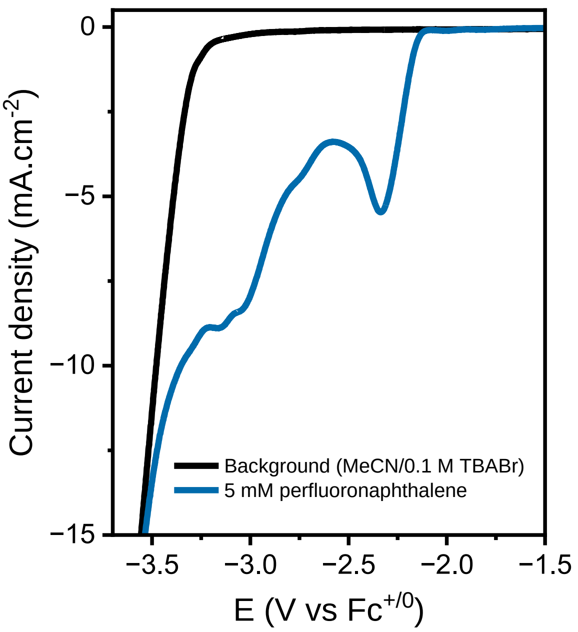

Figure S2.2. Cyclic voltammetry of perfluoronaphthalene in MeCN in reduction. Working electrode: glassy carbon, counter electrode: graphite, Ref: Ag/$AgNO_3$ (0.01 M $AgNO_3$ in MeCN/0.1 M $TBAPF_6$), dry and N_2-saturated MeCN /0.1 M TBABr, 5.0 mM perfluoronaphthalene, room temperature (~ 20°C), 100 mV.s^{-1}.

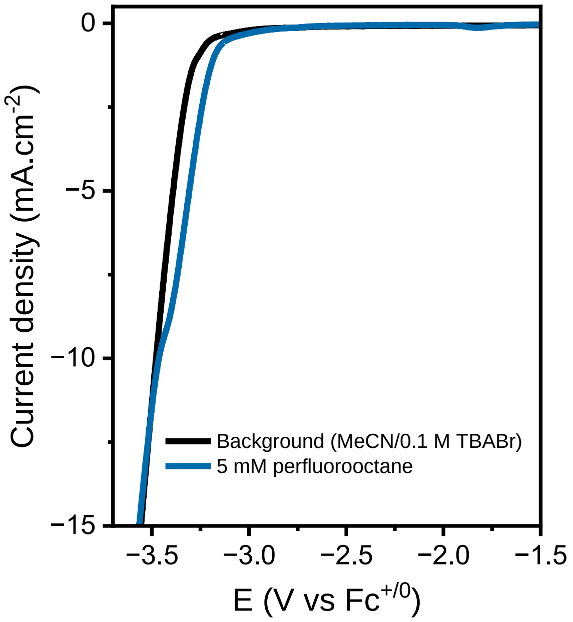

Figure S2.3. Cyclic voltammetry of perfluorooctane in MeCN in reduction. Working electrode: glassy carbon, counter electrode: graphite, Ref: Ag/$AgNO_3$ (0.01 M $AgNO_3$ in MeCN/0.1 M $TBAPF_6$), dry and N_2-saturated MeCN/0.1 M TBABr, 5.0 mM perfluorooctane, room temperature (~ 20°C), 100 mV.s^{-1}.

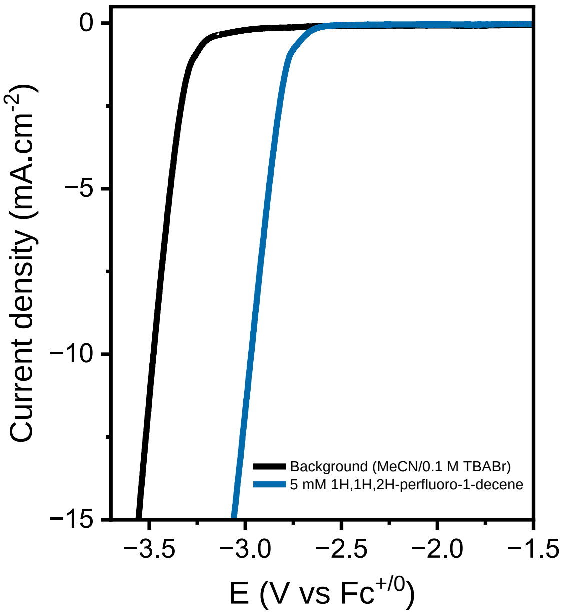

Figure S2.4. Cyclic voltammetry of 1H,1H,2H-perfluoro-1-decene in MeCN in reduction. WE: glassy carbon, counter electrode: graphite, Ref: Ag/$AgNO_3$ (0.01 M $AgNO_3$ in MeCN/0.1 M $TBAPF_6$), dry and N_2-saturated MeCN/0.1 M TBABr, 5.0 mM 1H,1H,2H-perfluoro-1-decene, room temperature (~ 20°C), 100 mV.s^{-1}.

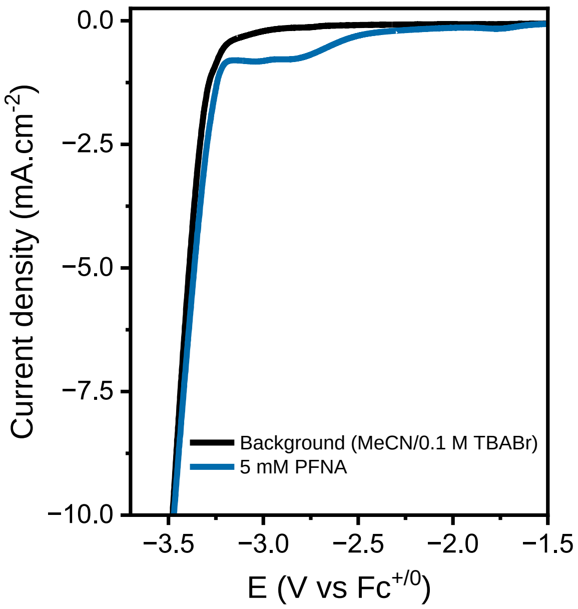

Figure S2.5. Cyclic voltammetry of PFNA in MeCN in reduction. WE: glassy carbon, counter electrode: graphite, Ref: Ag/$AgNO_3$ (0.01 M $AgNO_3$ in MeCN/0.1 M $TBAPF_6$), dry and N_2-saturated MeCN/0.1 M TBABr, 5.0 mM PFNA, room temperature (~ 20°C), 100 $mV.s^{-1}$.

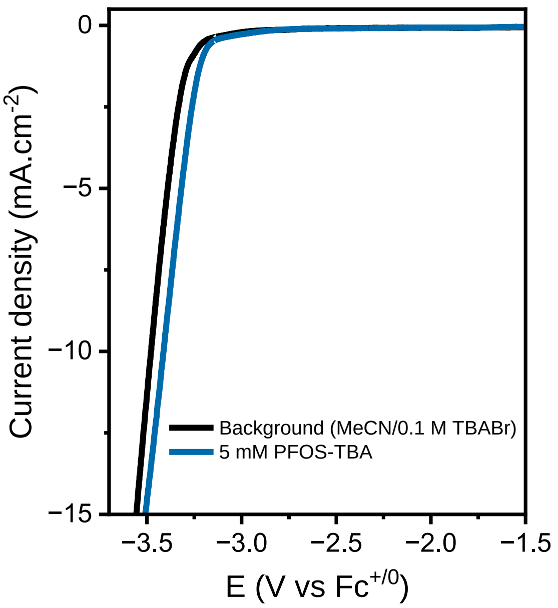

Figure S2.6. Cyclic voltammetry of PFOS-TBA in MeCN in reduction. WE: glassy carbon, counter electrode: graphite, Ref: Ag/$AgNO_3$ (0.01 M $AgNO_3$ in MeCN/0.1 M $TBAPF_6$), dry and N_2-saturated MeCN/0.1 M TBABr, 5.0 mM PFOS-TBA, room temperature (~ 20°C), 100 $mV.s^{-1}$.

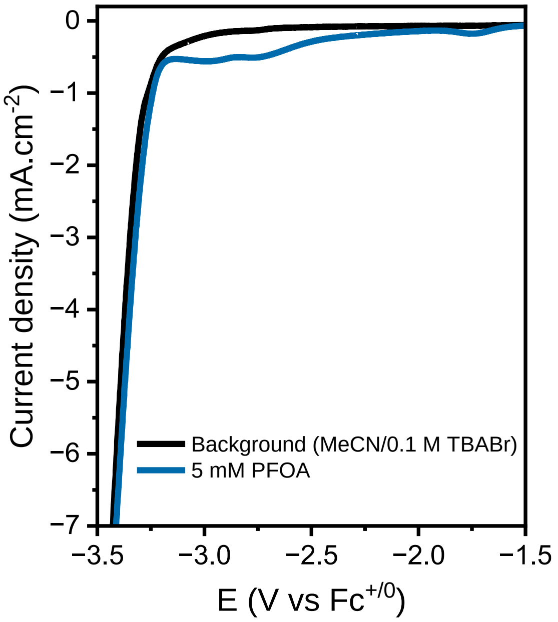

Figure S2.7. Cyclic voltammetry of PFOA in MeCN in reduction. WE: glassy carbon, counter electrode: graphite, Ref: Ag/$AgNO_3$ (0.01 M $AgNO_3$ in MeCN/0.1 M $TBAPF_6$), dry and N_2-saturated MeCN/0.1 M TBABr, 5.0 mM PFOA, room temperature (~ 20°C), 100 mV.s^{-1}.

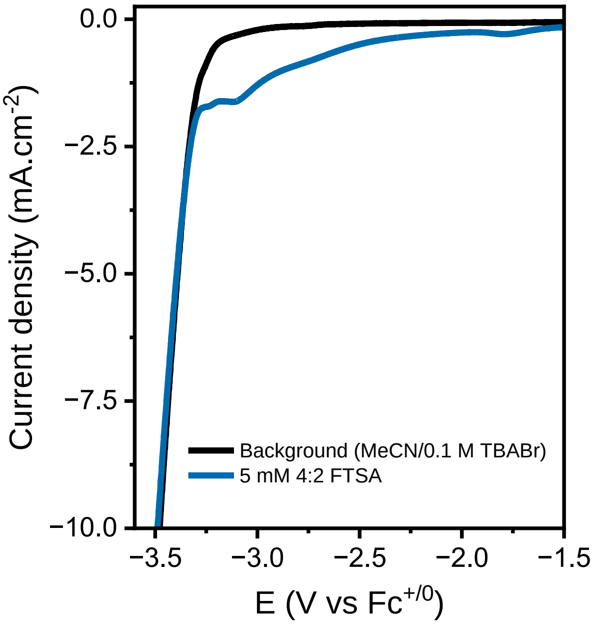

Figure S2.8. Cyclic voltammetry of 4:2 FTSA in MeCN in reduction. WE: glassy carbon, counter electrode: graphite, Ref: Ag/$AgNO_3$ (0.01 M $AgNO_3$ in MeCN/0.1 M $TBAPF_6$), dry and N_2-saturated MeCN/0.1 M TBABr, 5.0 mM 4:2 FTSA, room temperature (~ 20°C), 100 mV.s^{-1}.

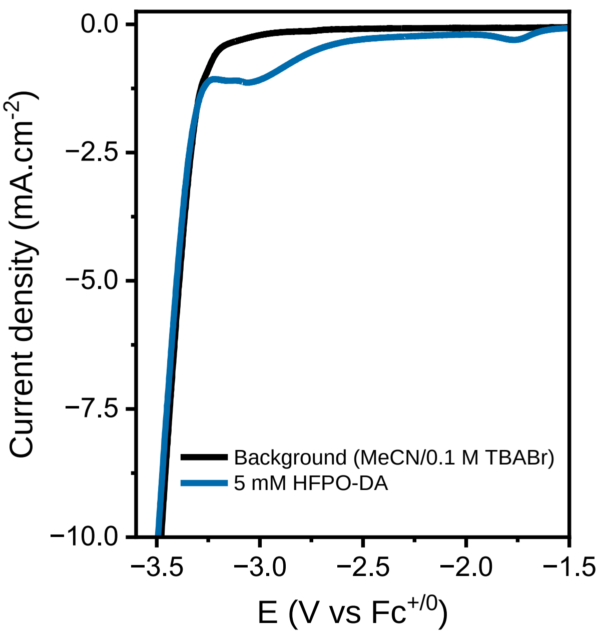

Figure S2.9. Cyclic voltammetry of HFPO-DA in MeCN in reduction. WE: glassy carbon, counter electrode: graphite, Ref: Ag/$AgNO_3$ (0.01 M $AgNO_3$ in MeCN/0.1 M $TBAPF_6$), dry and N_2-saturated MeCN/0.1 M TBABr, 5.0 mM HFPO-DA, room temperature (~ 20°C), 100 mV.s^{-1}.

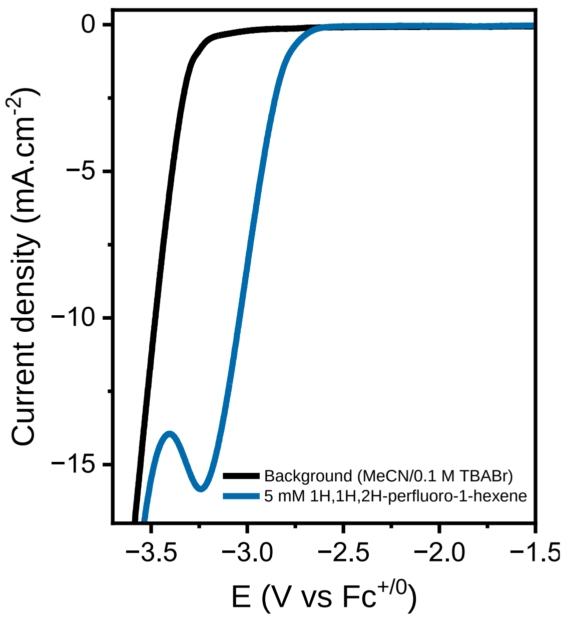

Figure S2.10. Cyclic voltammetry of 1H,1H,2H-perfluoro-1-hexene in MeCN in reduction. WE: glassy carbon, counter electrode: graphite, Ref: Ag/$AgNO_3$ (0.01 M $AgNO_3$ in MeCN/0.1 M $TBAPF_6$), dry and N_2-saturated MeCN/0.1 M TBABr, 5.0 mM 1H,1H,2H-perfluoro-1-hexene, room temperature (~ 20°C), 100 mV.s^{-1}.

b. Cyclic voltammetry of other compounds used in the study

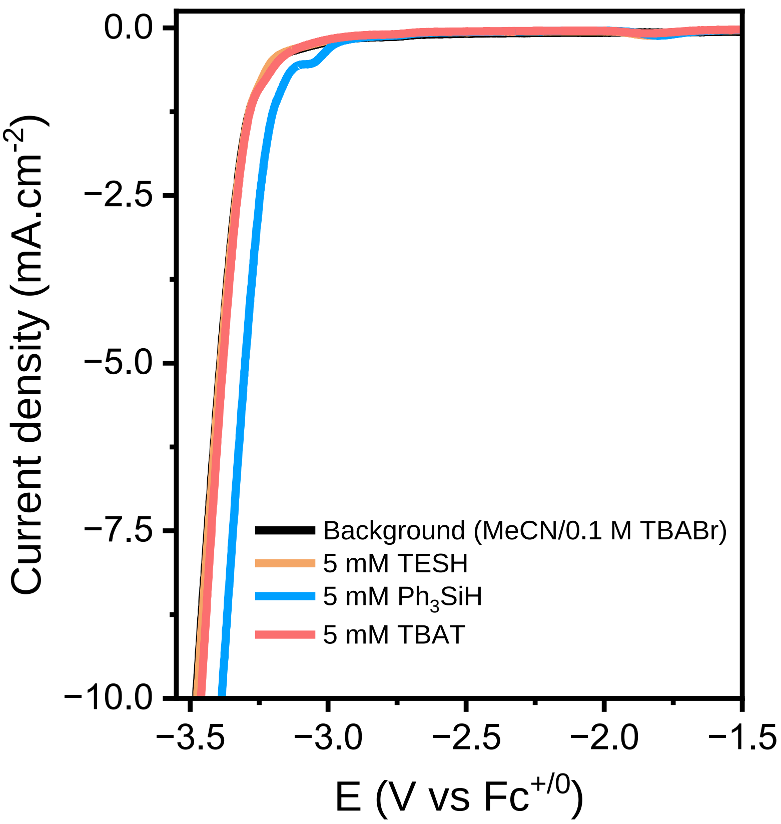

Figure S2.11. Cyclic voltammetry of TESH, Ph_3SiH and TBAT in MeCN in reduction. Working electrode: glassy carbon, counter electrode: graphite, Ref: Ag/$AgNO_3$ (0.01 M $AgNO_3$ in MeCN/0.1 M $TBAPF_6$), dry and N_2-saturated MeCN/0.1 M TBABr, 5.0 mM compounds, room temperature (~ 20°C), 100 mV.s^{-1}.

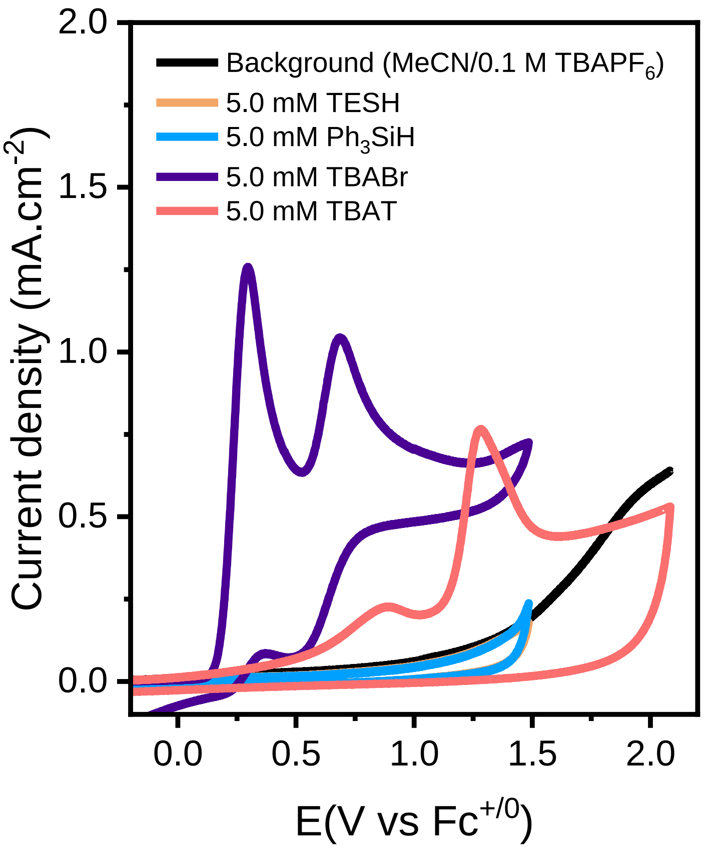

Figure S2.12. Cyclic voltammetry of TESH, Ph_3SiH, TBAT and TBABr in MeCN in oxidation. Working electrode: glassy carbon, counter electrode: graphite, Ref: $Ag/AgNO_3$ (0.01 M $AgNO_3$ in MeCN/0.1 M $TBAPF_6$), dry and N_2-saturated MeCN/0.1 M $TBAPF_6$, 5.0 mM compounds, room temperature (~ 20°C), 100 mV.s^{-1}.

3. Synthesis of NaF and KF from TESF

Although being one of the strongest bonds in organic chemistry, the Si-F bond can be activated to release fluoride. Early reports include the use of sodium alkoxides ($NaOCH_3$ and $NaOC_2H_5$) to form R_3SiOCH_3 or $R_3SiOC_2H_5$ from the fluorosilane R_3SiF, with R an alkyl group.[2,3] Here, following similar reactivity, we show that it is possible to precipitate NaF and KF from TESF in THF by reaction with sodium and potassium tert-butoxide. Sodium and potassium tert-butoxide were chosen for their solubility in THF.

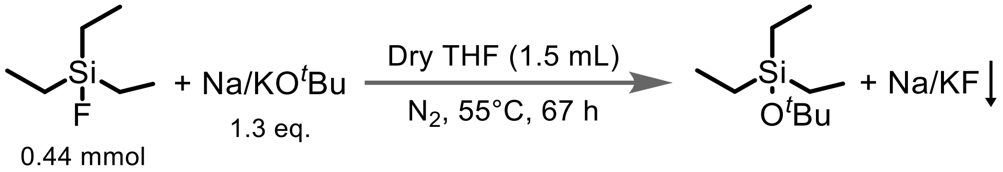

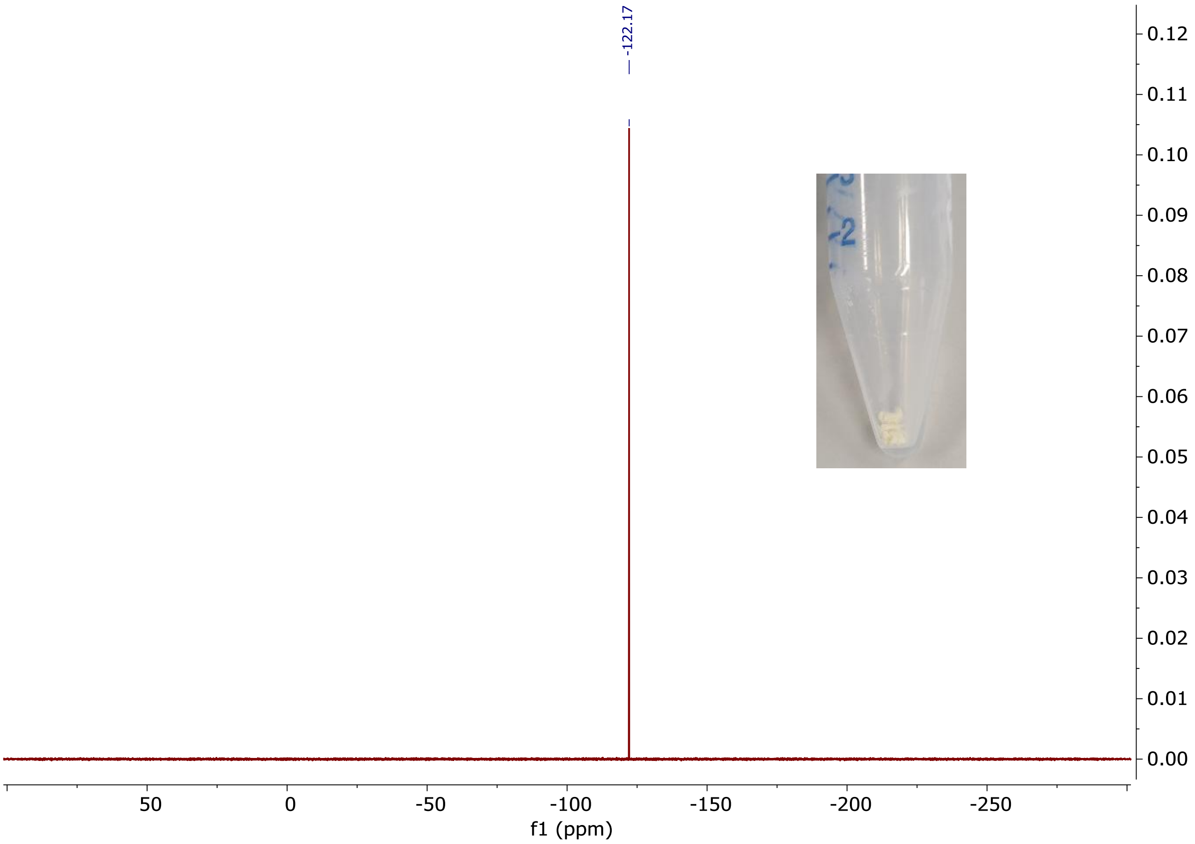

Figure S3.1. ^{19}F NMR (376 MHz, D_2O) spectrum of the cream white solid (see insert) obtained after reaction between TESF and sodium tert-butoxide.

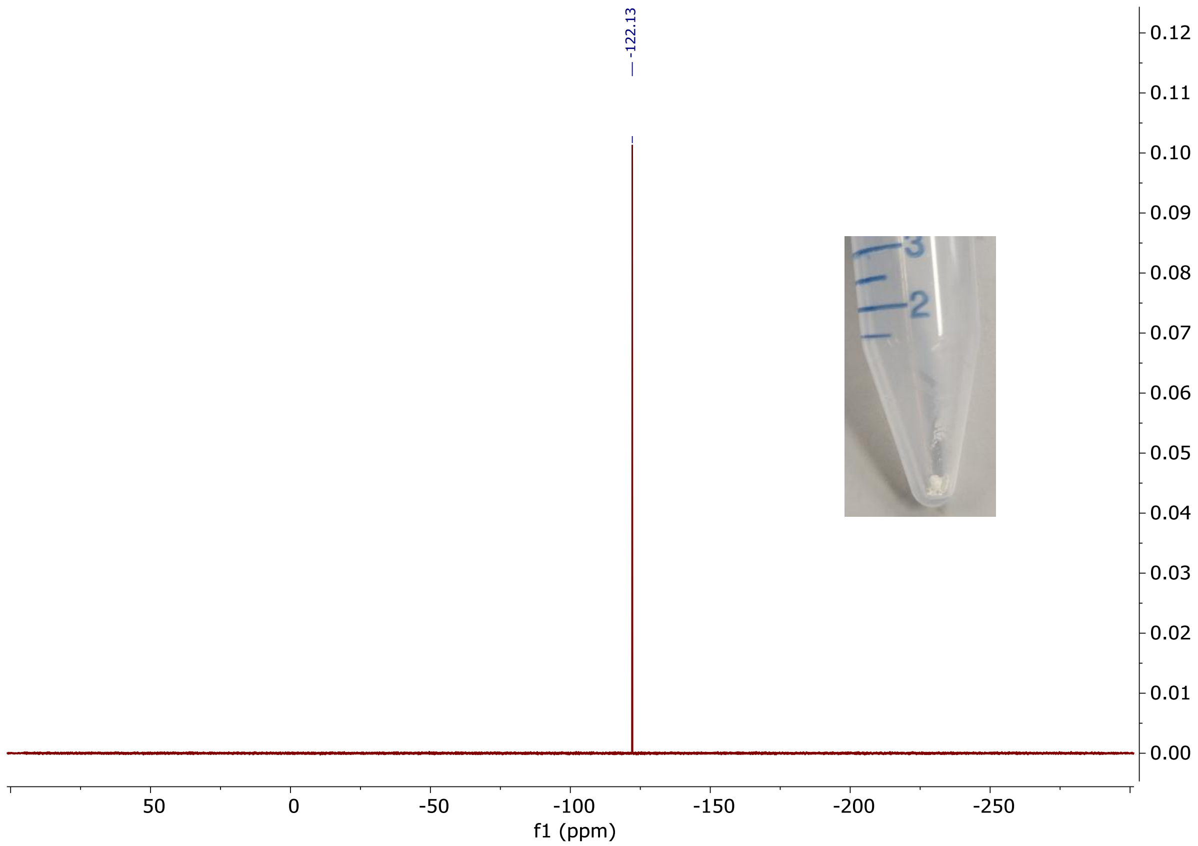

Figure S3.2. ^{19}F NMR (376 MHz, D_2O) spectrum of the white solid (see insert) obtained after reaction between TESF and potassium tert-butoxide.

4. Divided cell electrolysis in acetone – effect of the cathode material

The selective formation of F^- in acetone and the associated yield are independent of the cathode material. The corresponding NMR spectra are in **Figures S8.13-15**.

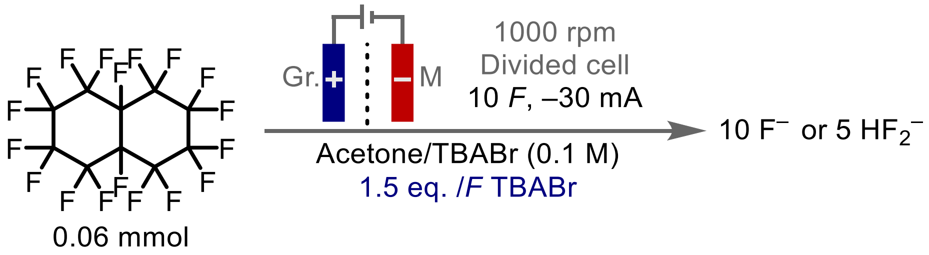

Cathode material	S. Steel	Zn	Ni
Selectivity (F^-:HF_2^-)	100%:0%	100%:0%	100%:0%
Total F yield[a]	39%	36%	39%

[a] Yields are given relative to 18 fluorine per PFD. Given that for the purposes of optimization only 10 *F* were passed, the maximum achievable yield was 56% here.

5. F^-/HF_2^- selectivity – Effect of the presence of water in the electrolysis medium

a. Karl Fischer titration of the different solvent/supporting salt systems used in the study

Table S5.1. Water content in different solvent/supporting salt systems as determined by Karl Fischer titration.

Solvent system	Water content (ppm)
Acetone	108.9 ± 11.0
THF	4.0 ± 1.3
MeCN	13.6 ± 1.8
MeCN/0.1 M TBABr	42.1 ± 4.4
MeCN/0.1 M $TBAClO_4$	665.0 ± 30.9

b. Electrolyses of PFD in THF with varying amounts of water

Electrolyses were conducted in dry THF/0.2 M TBABr (~ 60 ppm of water – see above) and in dry THF/0.2 M TBABr with addition of 500, 1000, 2000 and 5000 ppm of water, **Figure S5.1**. The corresponding NMR spectra are in **Figures S8.38-41**. The reactivity of fluoride is dampened through solvation by water. Thus, the formation of HF_2^-, which likely arises from β-deprotonation of TBA^+ *via* Hofmann elimination by strongly basic fluoride, is observed to gradually decrease in electrolyses with increasing water content, **Figure S5.1**.

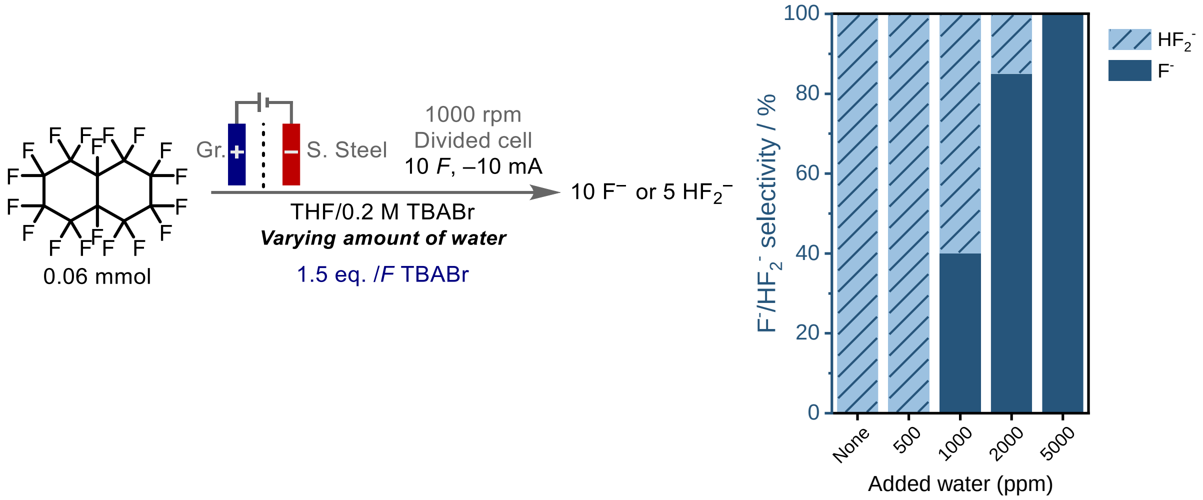

Figure S5.1. F^-/HF_2^- selectivity obtained in the divided cell electrolysis of PFD in THF/0.2 M TBABr with varying amount of water added to the electrolyte. Percentages of species are rounded to the nearest 5.

Interestingly, similar F^-/HF_2^- selectivity trend was obtained when *indirectly* adding water to THF through the use of the wet salt $TBAClO_4$, **Figure S5.2** and **Table S5.1**. Electrolyses were conducted in dry THF/{0.1 M TBABr + 0.1 M $TBAClO_4$} (~ 680 ppm of water) and dry THF/0.2 M $TBAClO_4$ (~ 1300 ppm of water). The corresponding NMR spectra are in **Figures S8.42,43.**

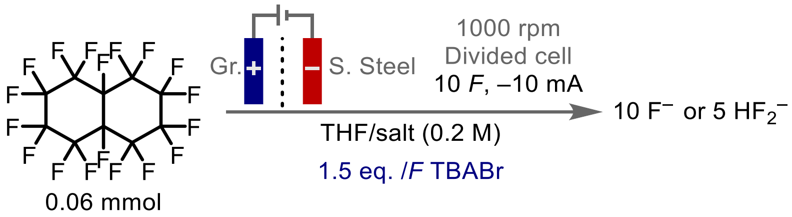

Conditions	dry THF<br>0.2 M TBABr	dry THF<br>0.1 M TBABr<br>+ 0.1 M $TBAClO_4$	dry THF<br>0.2 M $TBAClO_4$
Selectivity (F^-:HF_2^-)	0%:100%	0%:100%	50%:50%

Figure S5.2. F^-/HF_2^- selectivity obtained in the divided cell electrolysis of PFD in THF with the use of $TBAClO_4$ as supporting salt. Percentages of species are rounded to the nearest 5.

c. Mixing of dry sources of fluoride in acetone and THF

Dry sources of fluoride (CsF and TMAF) were mixed (0.06 mmol fluoride salt, 3h, room temperature) in dry acetone and THF in the presence or the absence of a TBA^+ source to assess whether the same selectivity was obtained as without electrochemistry. Similar selectivity was obtained as with electrochemistry, albeit in low yield due to very poor solubility of CsF and TMAF in both solvents. The results also tend to show that the presence of TBA^+ is required in these conditions for HF_2^- formation (Hofmann elimination). The corresponding NMR spectra are in **Figures S8.44-51**.

Table S5.2. Fluorine species observed by 19F NMR when mixing 0.06 mmol of CsF or TMAF for 3h in acetone and THF with and without the presence of 0.1 M TBABr.

	**Dry acetone**	**Dry acetone + 0.1 M TBABr**	**Dry THF**	**Dry THF + 0.1 M TBABr**
Addition of CsF	Nothing on ^{19}F NMR	F^- peak	Nothing on ^{19}F NMR	Very small F^- peak
Addition of TMAF	F^- peak	F^- peak	Nothing on ^{19}F NMR	Very small F^- peak and small HF_2^- doublet

6. Divided cell electrolysis of PFD – E_{cell} values in different solvents

Table S6.1. E_{cell} values obtained when performing the divided cell electrolysis of PFD (0.06 mmol, 10 *F*) in different solvents.

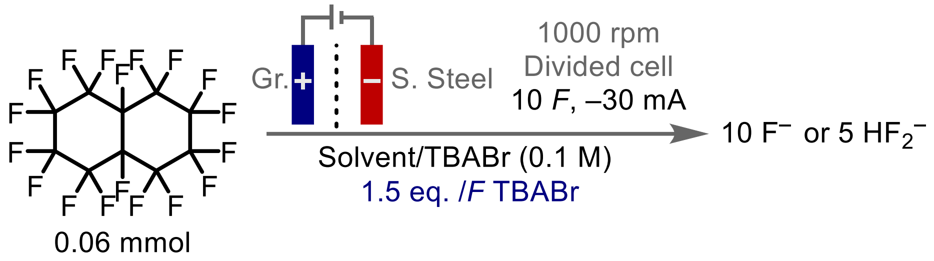

Cond. / E_{cell}[a] (V)	Acetone 0.1 M TBABr	MEK 0.1 M TBABr	DMSO 0.1 M TBABr	DMA 0.1 M TBABr	MeCN 0.1 M TBABr	THF[b] 0.2 M TBABr
Start	26	45	23	33	14	120[c]
End	30	50	22	39	13	90

[a] Ecell rounded to the nearest volt
[b] Reaction performed at -10 mA with 0.2 M of supporting salt to reduce the E_{cell}. Catholytes and anolytes heated with a heatgun prior to electrolysis to help solubilise TBABr
[c] E_{cell} quickly drops to ~ 85 V

7. TBAT synthesis in (un)divided cells

a. Attempts to synthesize TBAT in undivided cells

All attempts to synthesize TBAT in undivided cell conditions from PFAS and Ph_3SiH were unsuccessful. This is likely due to the very high sensitivity of TBAT to acids[4,5] (HBr is transiently generated with the bromide-mediated silane oxidation counter-electrode process) and to a lesser extent to TBAT's oxidative instability (**Figure S2.12**).

Table S7.1. Unsuccessful attempts to devise undivided cell conditions for TBAT synthesis

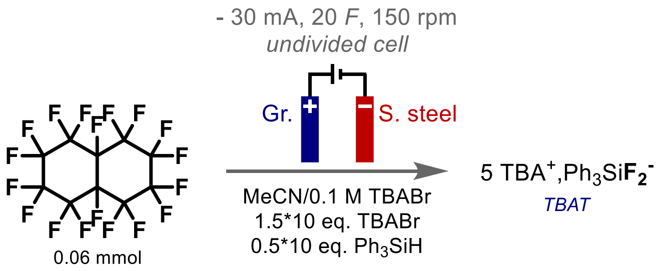

Entry	Deviation from standard conditions	Yield[a] (%)
1	None	0
2	Stainless steel sacrificial anode	0[b]
3	RVC anode	0
4	No current	0

[a] ^{19}F NMR yields, [b] 21% yield Ph_3SiF

b. TBAT yield as a function of the charge (*F*) passed in divided cells

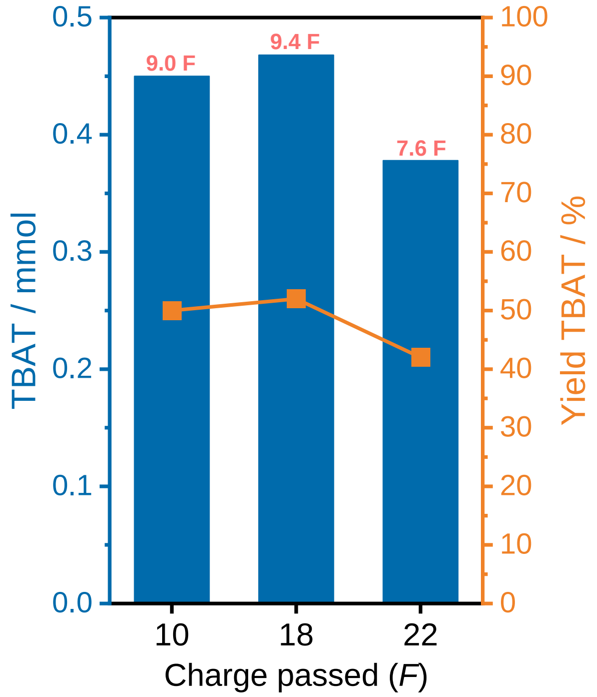

Figure S7.1. Influence of the charge passed (*F*) on the TBAT yield in divided cells.

c. Solid that precipitates after divided cell electrolysis for TBAT synthesis

Irrespective of the PFAS substrate, a cream white solid (**Figure S7.2**) precipitates after electrolysis when the crude is exposed to air. The spectroscopic data is in agreement with the formation of 1,1,1,3,3,3-hexaphenyldisiloxane, $(Ph_3Si)_2O$ (see below).[6] This likely arises from the formation of the triphenylsilyl radical anion upon competing electrochemical reduction of triphenylsilane (see cyclic voltammetry in **Figure S2.11**). Upon exposure of the crude to air, the triphenylsilyl radical anions react with oxygen to form $(Ph_3Si)_2O$. $(Ph_3Si)_2O$ was removed by centrifugation before getting ^{19}F NMR TBAT yields.

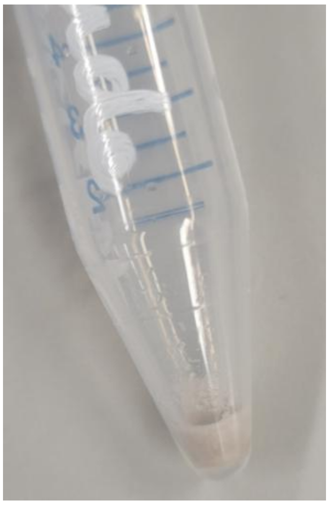

Figure S7.2. Solid that precipitates after electrolysis when the crude is exposed to air.

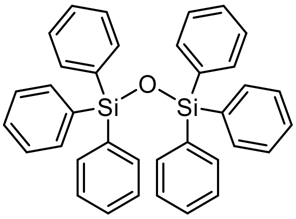

^{1}H NMR (500 MHz, $CDCl_3$): δ = 7.50-7.48 (m, 12H), 7.39 (t, 6H, *J*=7.3 Hz), 7.28 (t, 12H, *J*=7.5 Hz).
^{13}C NMR (126 MHz, $CDCl_3$): δ = 135.5, 135.2, 129.8, 127.7.
^{29}Si NMR (99 MHz, $CDCl_3$): δ = -18.63.

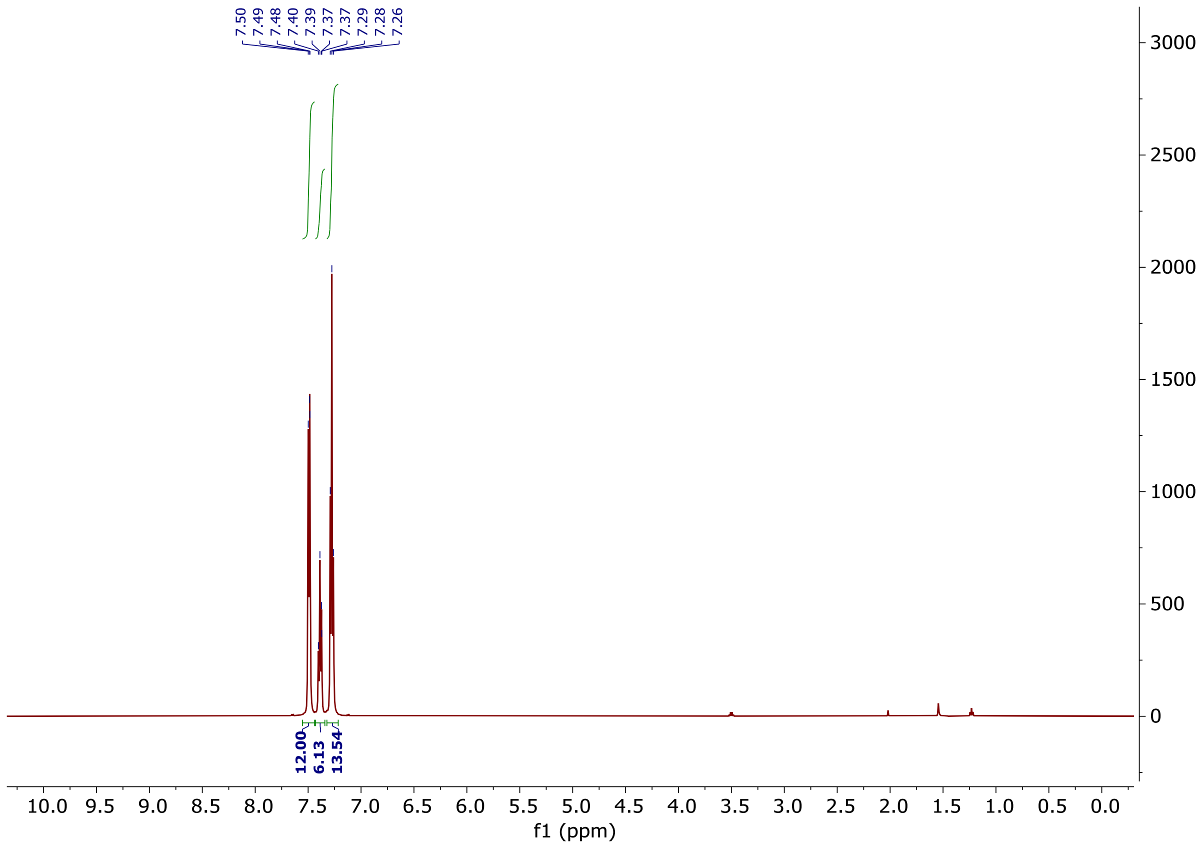

Figure S7.3. ^{1}H NMR (500 MHz, $CDCl_3$) spectrum of the solid that precipitates after electrolysis. Traces of diethyl ether (used to dry the solid), acetonitrile (electrolysis solvent) and water (from $CDCl_3$) are visible.

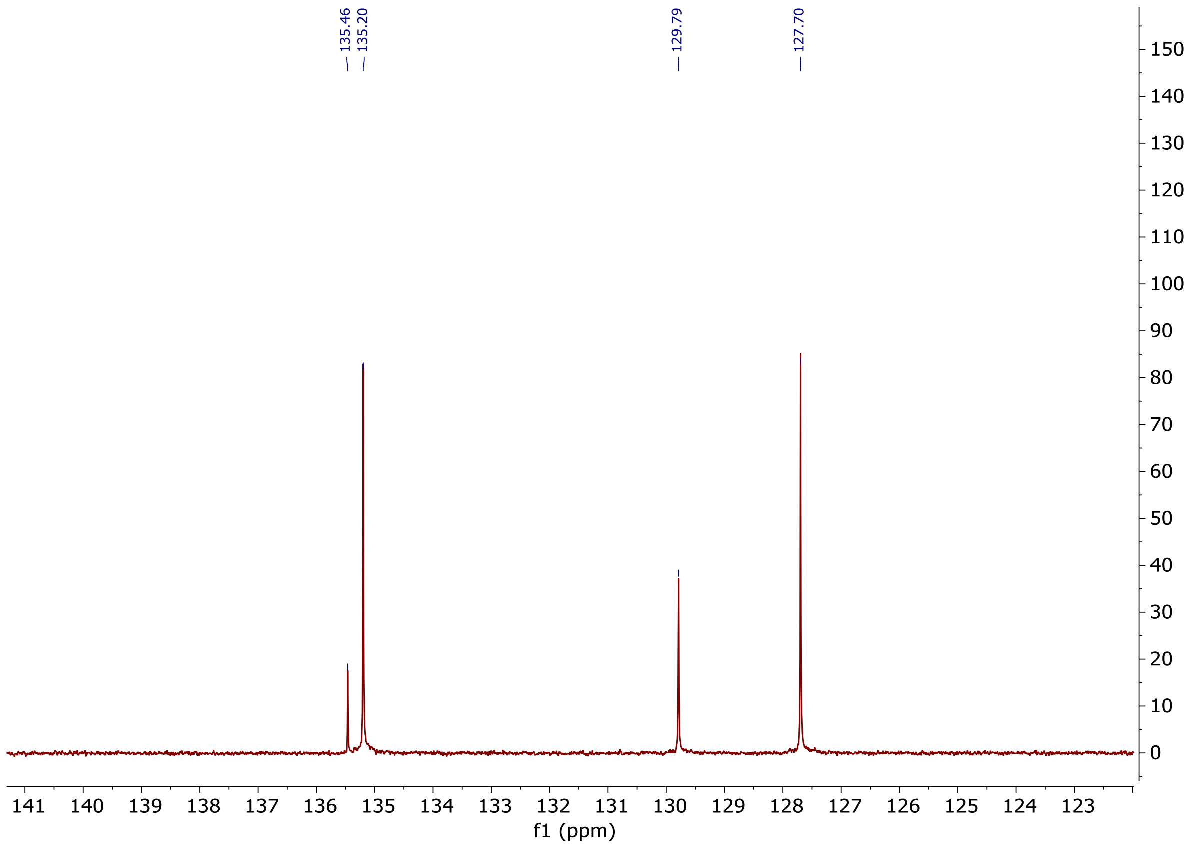

Figure S7.4. ^{13}C NMR (126 MHz, $CDCl_3$) spectrum of the solid that precipitates after electrolysis.

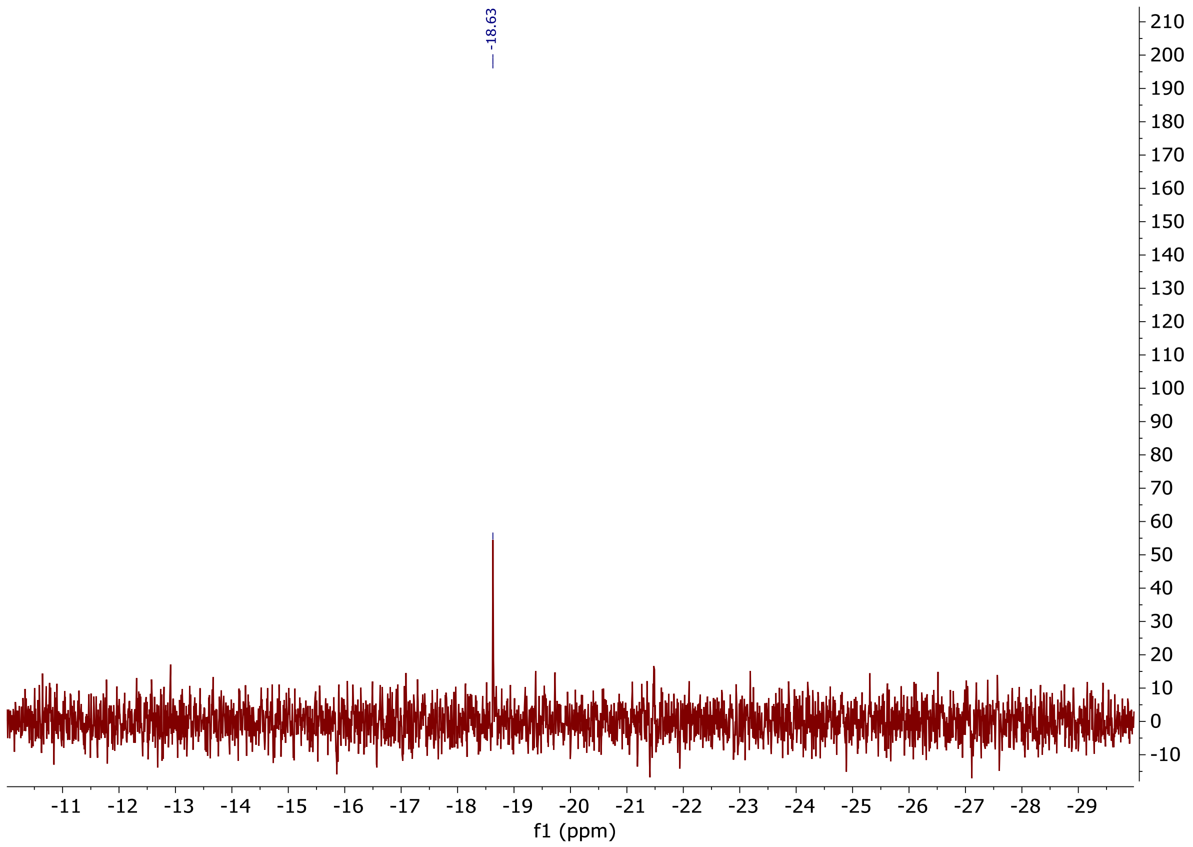

Figure S7.5. ^{29}Si (DEPT) NMR (99 MHz, $CDCl_3$) spectrum of the solid that precipitates after electrolysis.

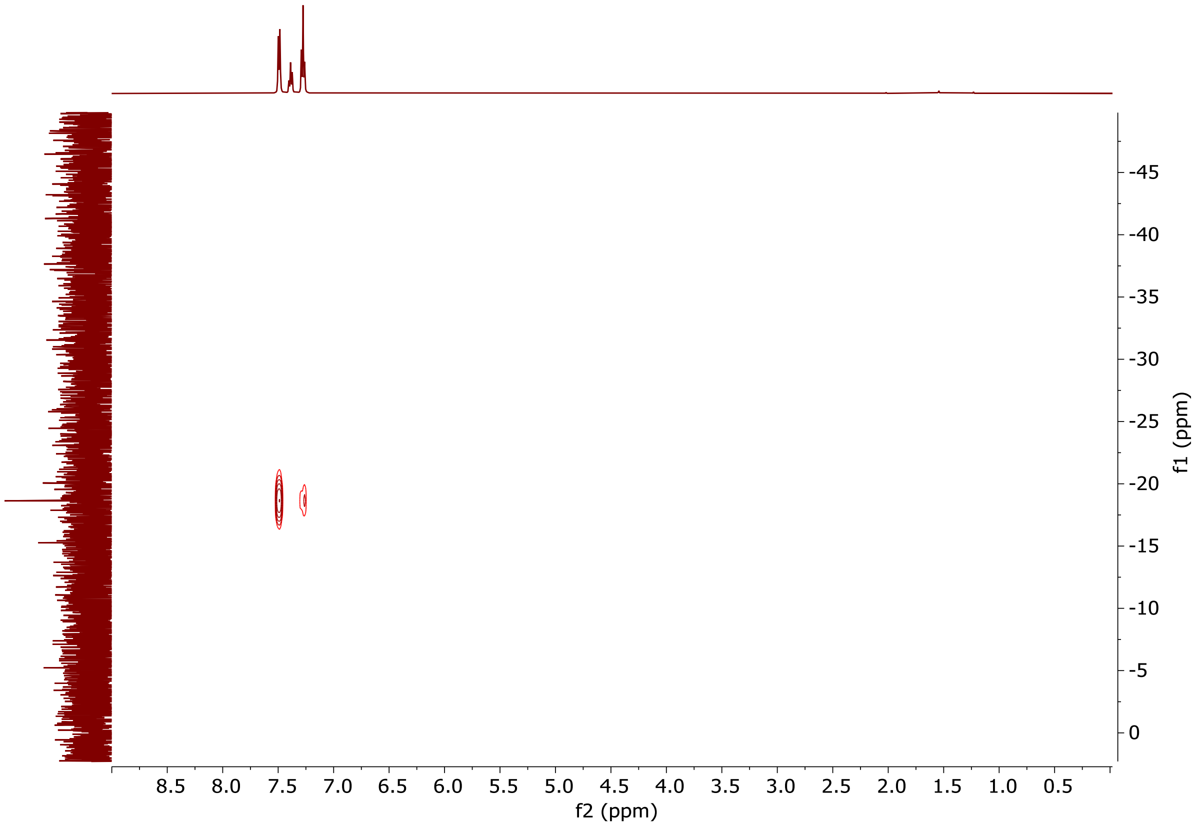

Figure S7.6. ^{1}H-^{29}Si HMBC of the solid that precipitates after electrolysis.

8. NMR spectra

a. Undivided cell electrolysis – TESF synthesis

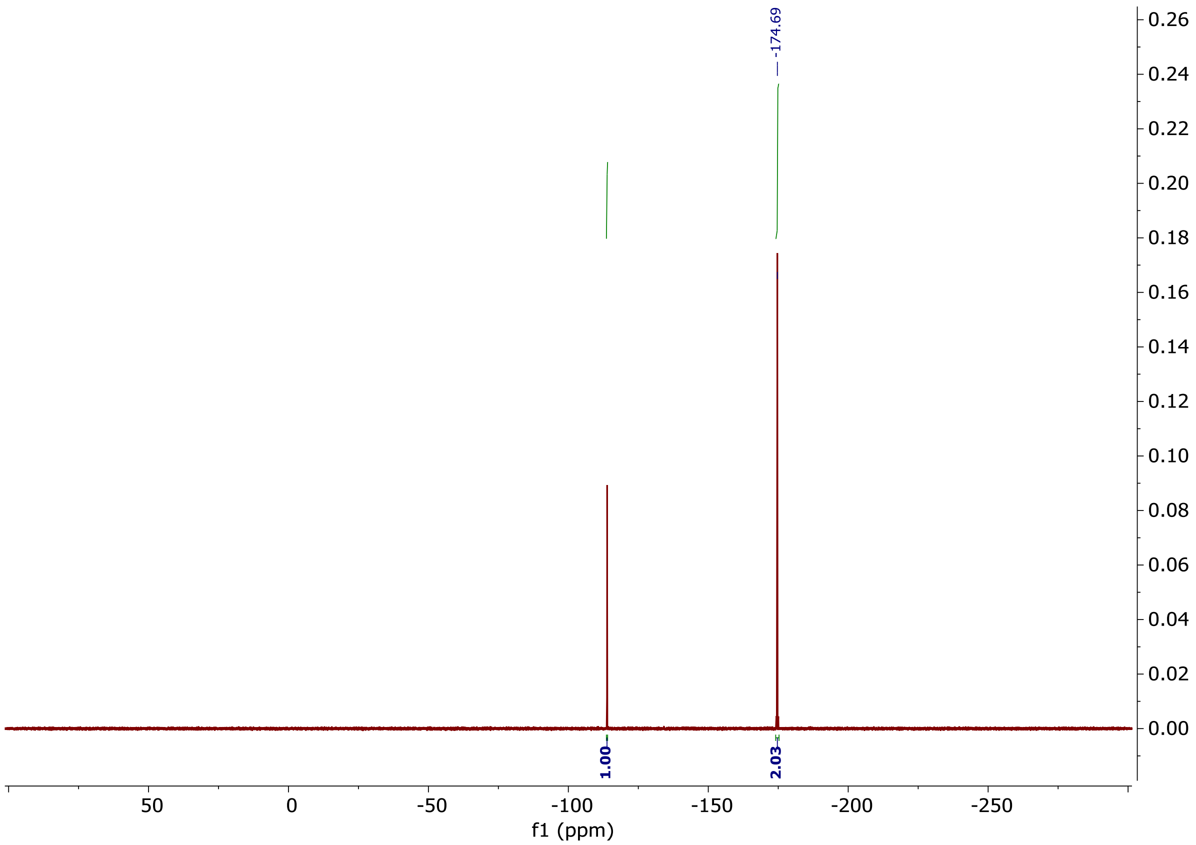

Figure S8.1. ^{19}F NMR (376 MHz) spectrum of the crude obtained after the 0.1 mmol scale undivided cell electrolysis for TESF synthesis from perfluorodecalin (24 µL) in dry acetonitrile with 50 µL of fluorobenzene as internal standard. The TESF peak is visible at -174.69 ppm.

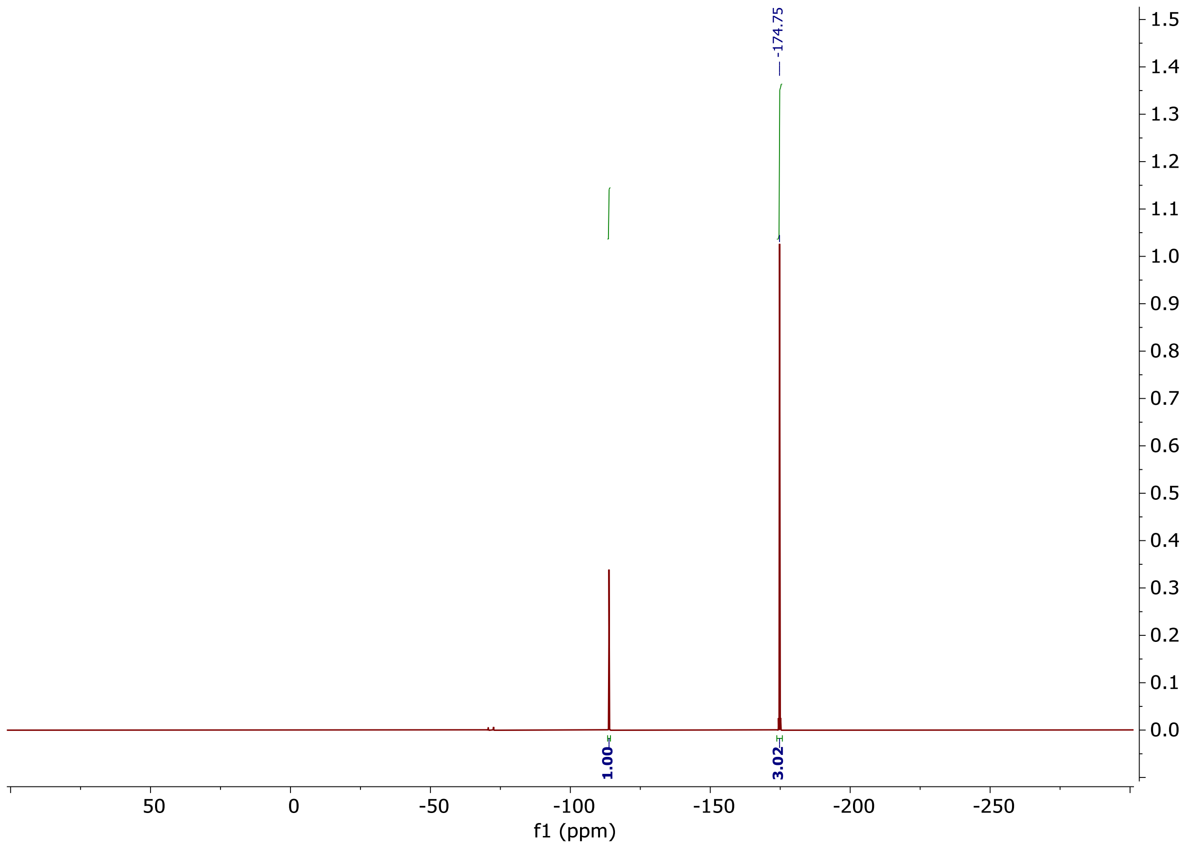

Figure S8.2. ^{19}F NMR (376 MHz) spectrum of the crude obtained after the 1.0 mmol scale undivided cell electrolysis for TESF synthesis from perfluorodecalin (242 µL) in dry acetonitrile with 400 µL of fluorobenzene as internal standard. The TESF peak is visible at -174.75 ppm.

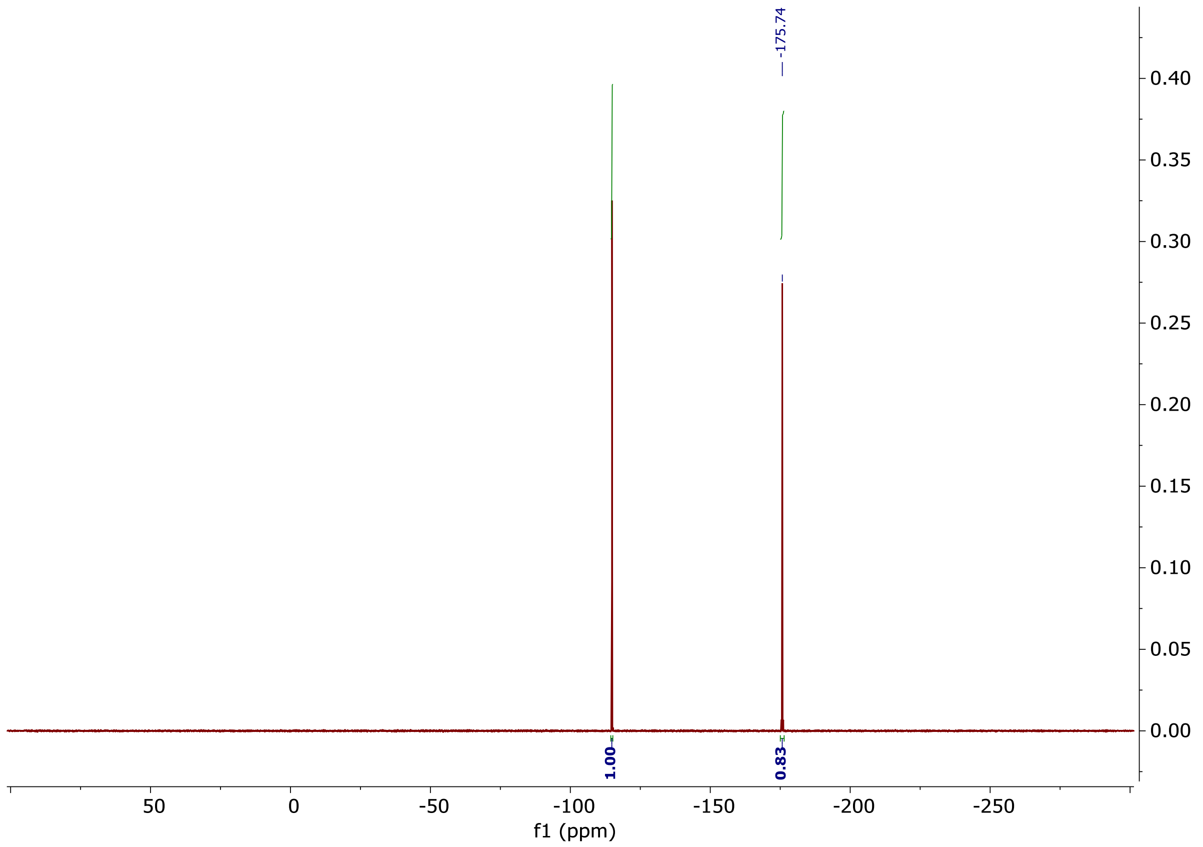

Figure S8.3. ^{19}F NMR (376 MHz) spectrum of the crude obtained after the 0.1 mmol scale undivided cell electrolysis for TESF synthesis from perfluorooctane (25 µL) in dry acetonitrile with 50 µL of fluorobenzene as internal standard. The TESF peak is visible at -175.74 ppm.

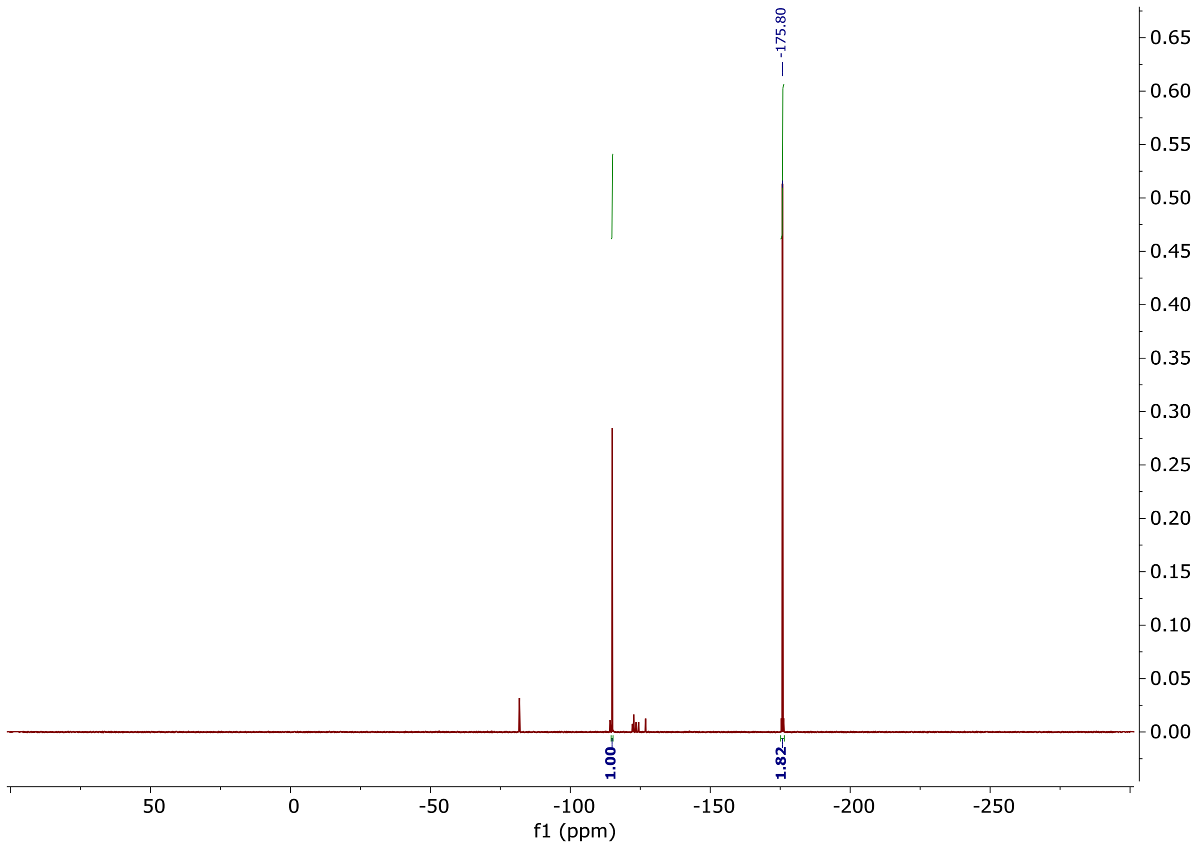

Figure S8.4. ^{19}F NMR (376 MHz) spectrum of the crude obtained after the 0.1 mmol scale undivided cell electrolysis for TESF synthesis from 1H,1H,2H-perfluoro-1-decene (27 µL) in dry acetonitrile with 50 µL of fluorobenzene as internal standard. The TESF peak is visible at -175.80 ppm.

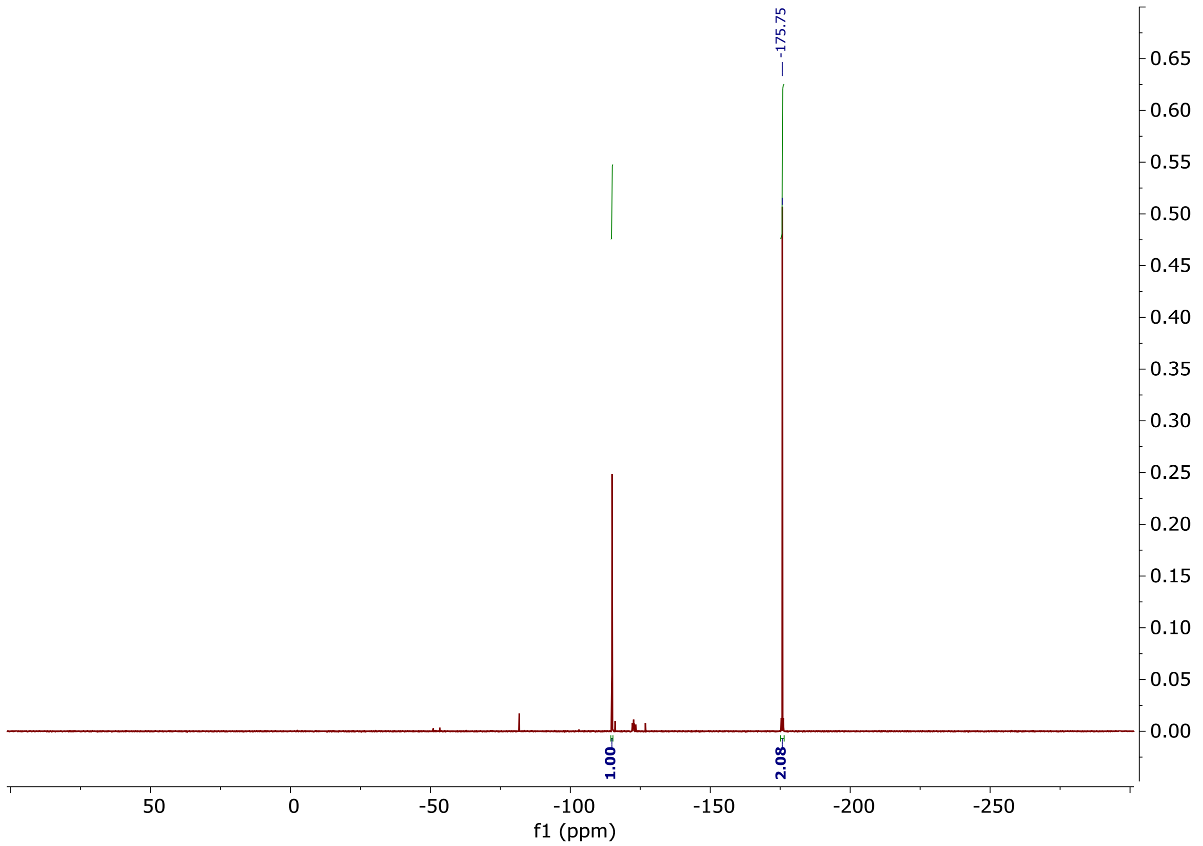

Figure S8.5. ^{19}F NMR (376 MHz) spectrum of the crude obtained after the 0.1 mmol scale undivided cell electrolysis for TESF synthesis from PFNA (47 mg) in dry acetonitrile with 50 µL of fluorobenzene as internal standard. The TESF peak is visible at -175.75 ppm.

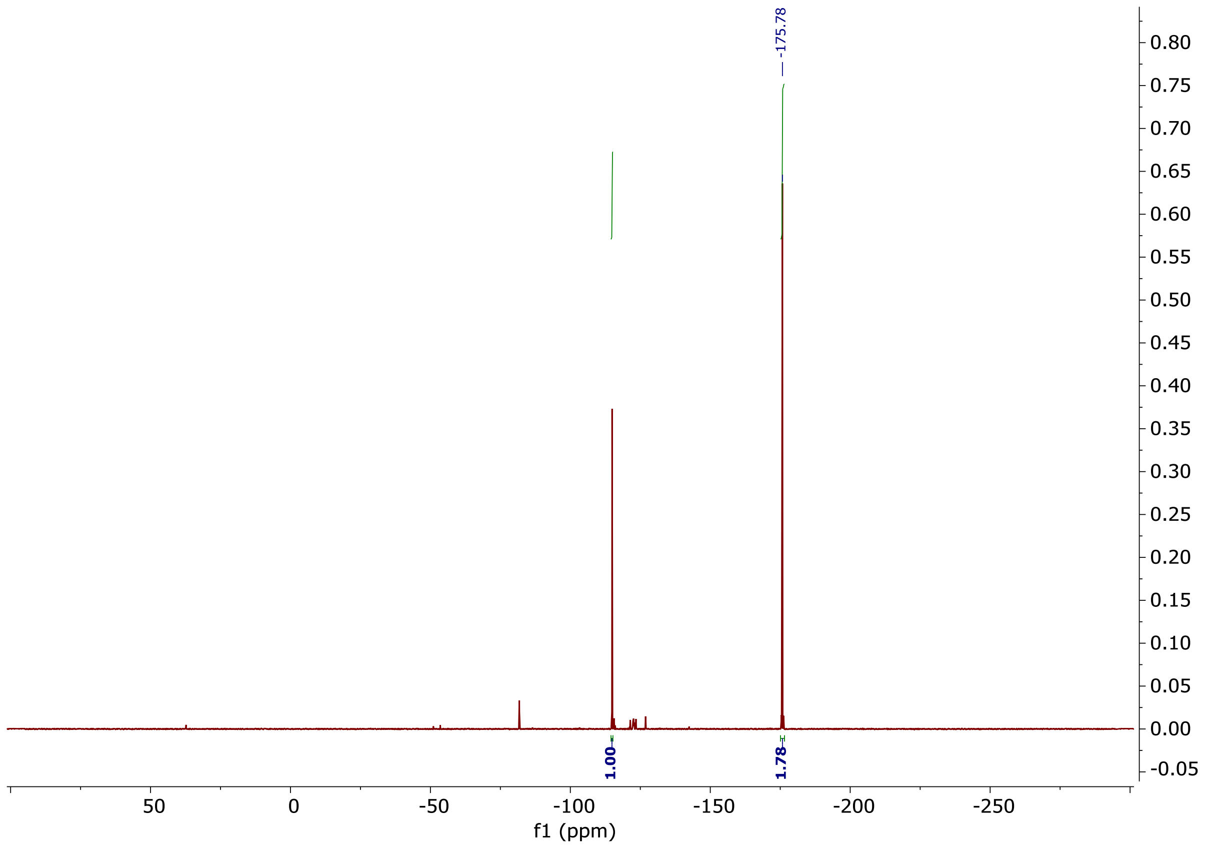

Figure S8.6. ^{19}F NMR (376 MHz) spectrum of the crude obtained after the 0.1 mmol scale undivided cell electrolysis for TESF synthesis from PFOS-K (54 mg) in dry acetonitrile with 50 µL of fluorobenzene as internal standard. The TESF peak is visible at -175.78 ppm.

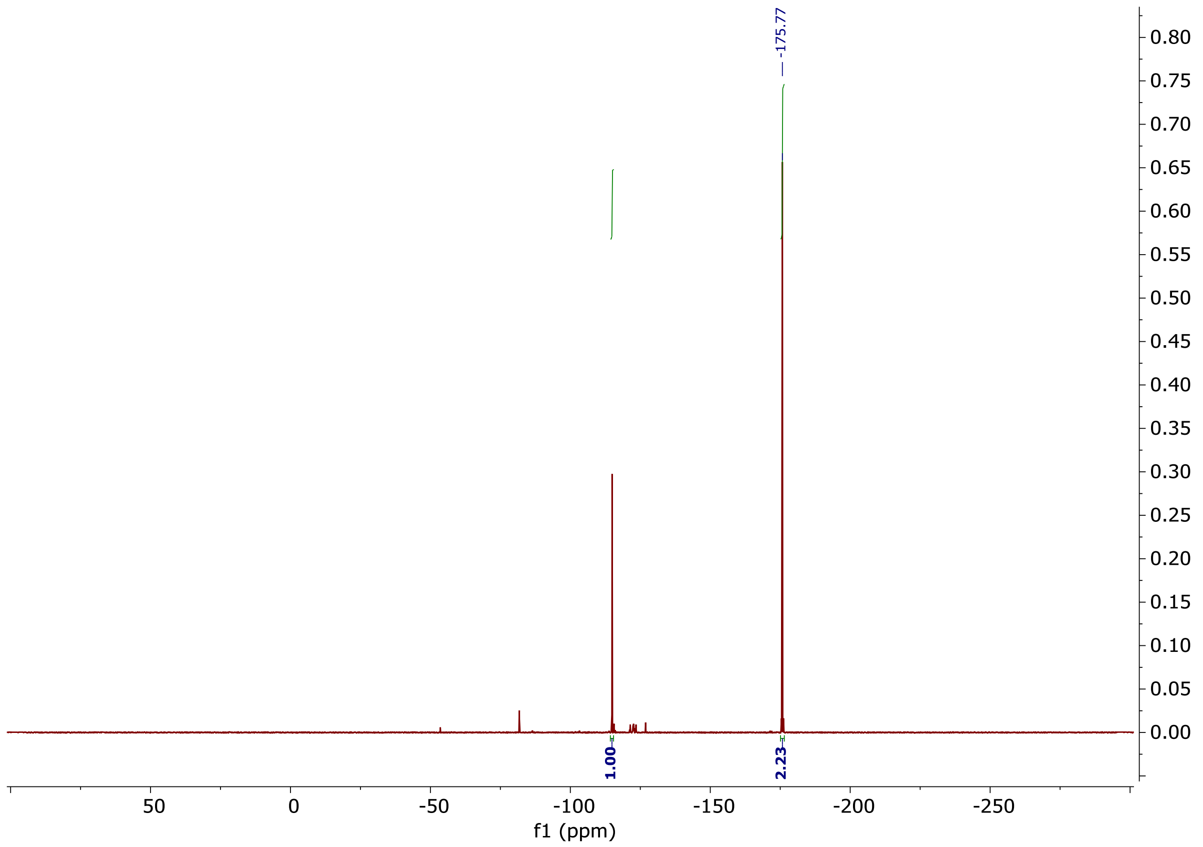

Figure S8.7. ^{19}F NMR (376 MHz) spectrum of the crude obtained after the 0.1 mmol scale undivided cell electrolysis for TESF synthesis from PFOS-TBA (74 mg) in dry acetonitrile with 50 µL of fluorobenzene as internal standard. The TESF peak is visible at -175.77 ppm.

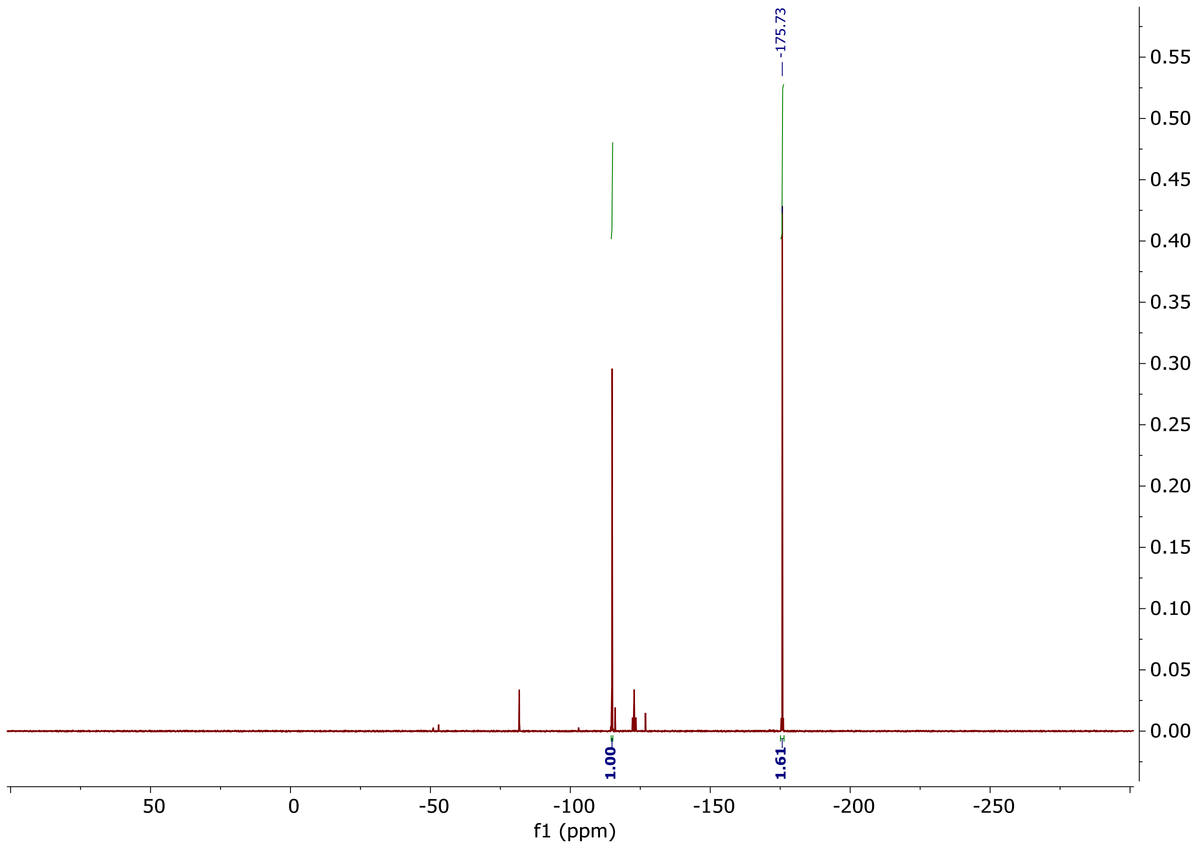

Figure S8.8. ^{19}F NMR (376 MHz) spectrum of the crude obtained after the 0.1 mmol scale undivided cell electrolysis for TESF synthesis from PFOA (42 mg) in dry acetonitrile with 50 µL of fluorobenzene as internal standard. The TESF peak is visible at -175.73 ppm.

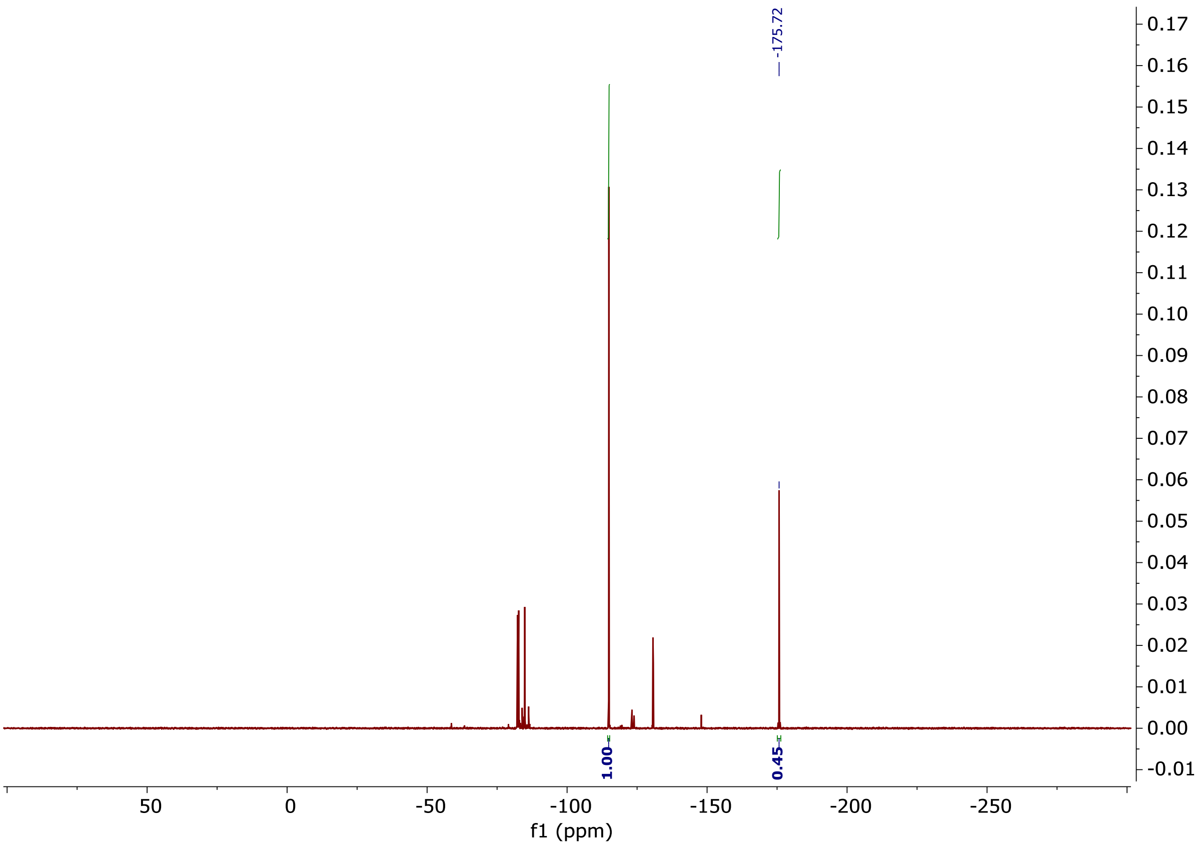

Figure S8.9. ^{19}F NMR (376 MHz) spectrum of the crude obtained after the 0.1 mmol scale undivided cell electrolysis for TESF synthesis from HFPO-DA (19 µL) in dry acetonitrile with 50 µL of fluorobenzene as internal standard. The TESF peak is visible at -175.72 ppm.

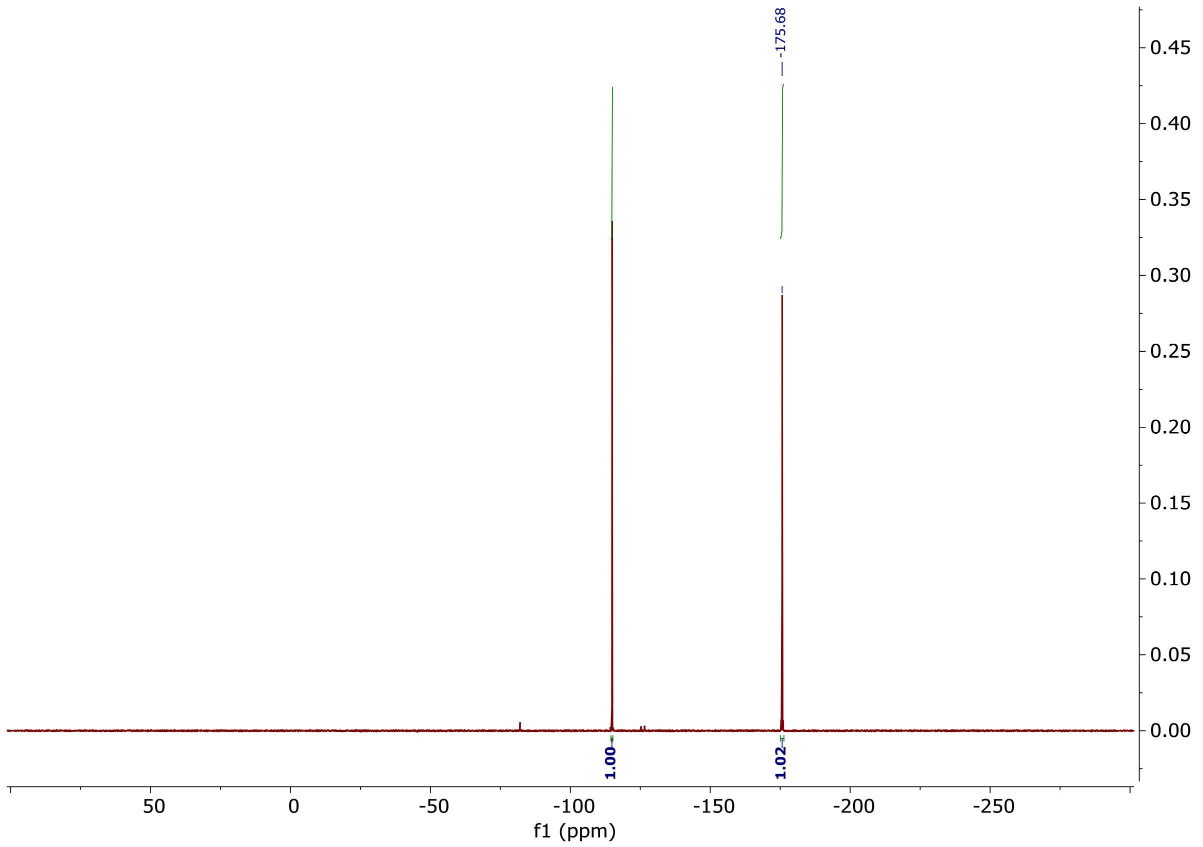

Figure S8.10. ^{19}F NMR (376 MHz) spectrum of the crude obtained after the 0.1 mmol scale undivided cell electrolysis for TESF synthesis from 1H,1H,2H-perfluoro-1-hexene (17 µL) in dry acetonitrile with 50 µL of fluorobenzene as internal standard. The TESF peak is visible at -175.68 ppm.

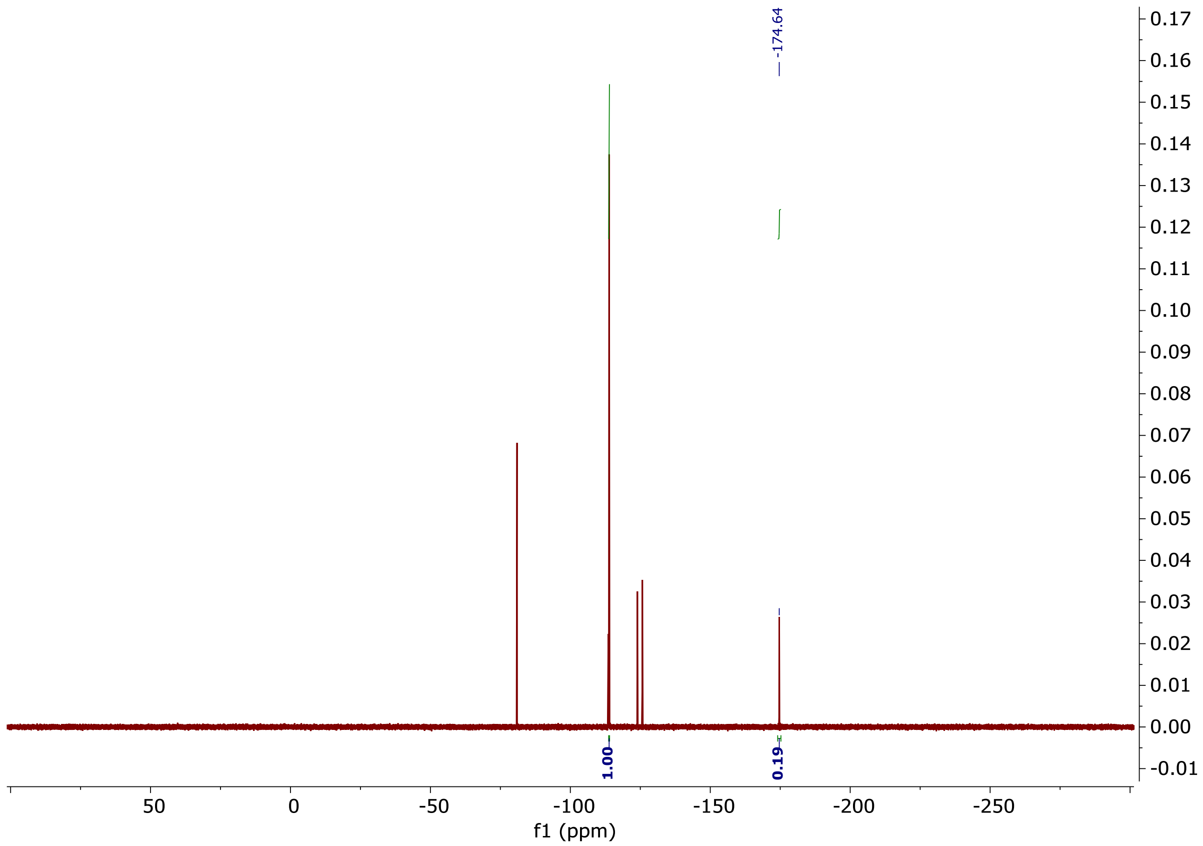

Figure S8.11. ^{19}F NMR (376 MHz) spectrum of the crude obtained after the 0.1 mmol scale undivided cell electrolysis for TESF synthesis from 4:2 FTSA (33 mg) in dry acetonitrile with 50 µL of fluorobenzene as internal standard. The TESF peak is visible at -174.64 ppm.

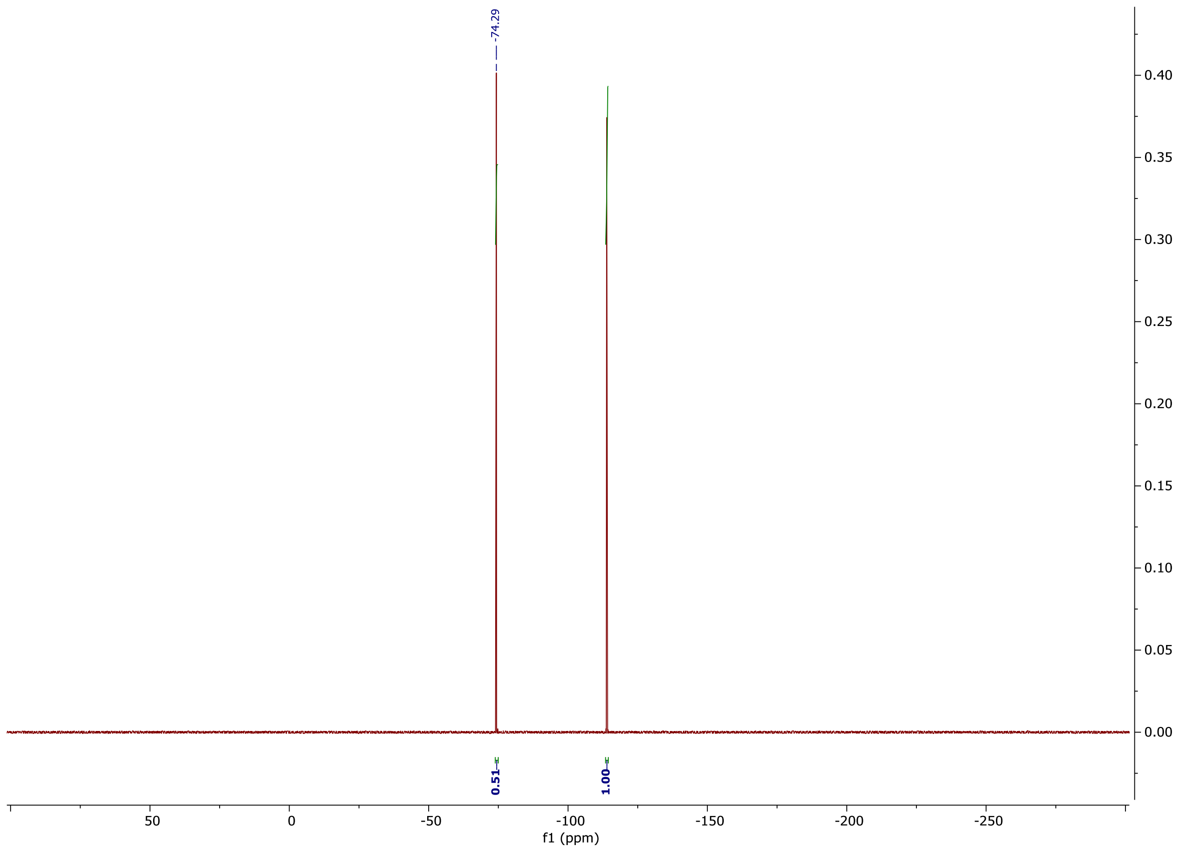

Figure S8.12. ^{19}F NMR (376 MHz) spectrum of the crude obtained after the 0.1 mmol scale undivided cell electrolysis for TESF synthesis from TFA in dry acetonitrile with 50 µL of fluorobenzene as internal standard. No TESF peak is visible. The TFA peak is visible at -74.29 ppm.

b. Divided cell electrolysis of PFAS – F^-/HF_2^- selectivity

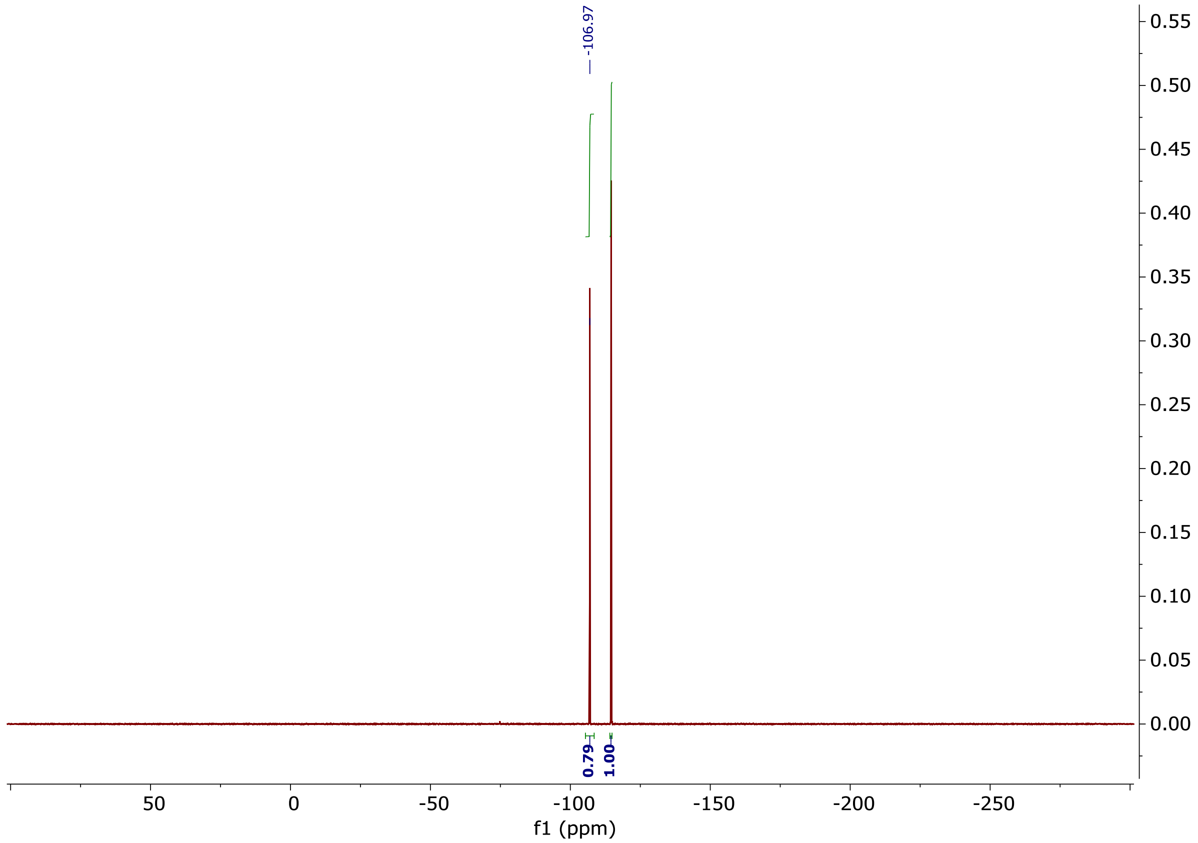

Figure S8.13. ^{19}F NMR (376 MHz) spectrum of the crude obtained after the 0.06 mmol scale divided cell electrolysis of perfluorodecalin in dry acetone with a stainless steel cathode (10 *F*) and with 50 µL of fluorobenzene as internal standard. The fluoride (F^-) peak is visible at -106.97 ppm.

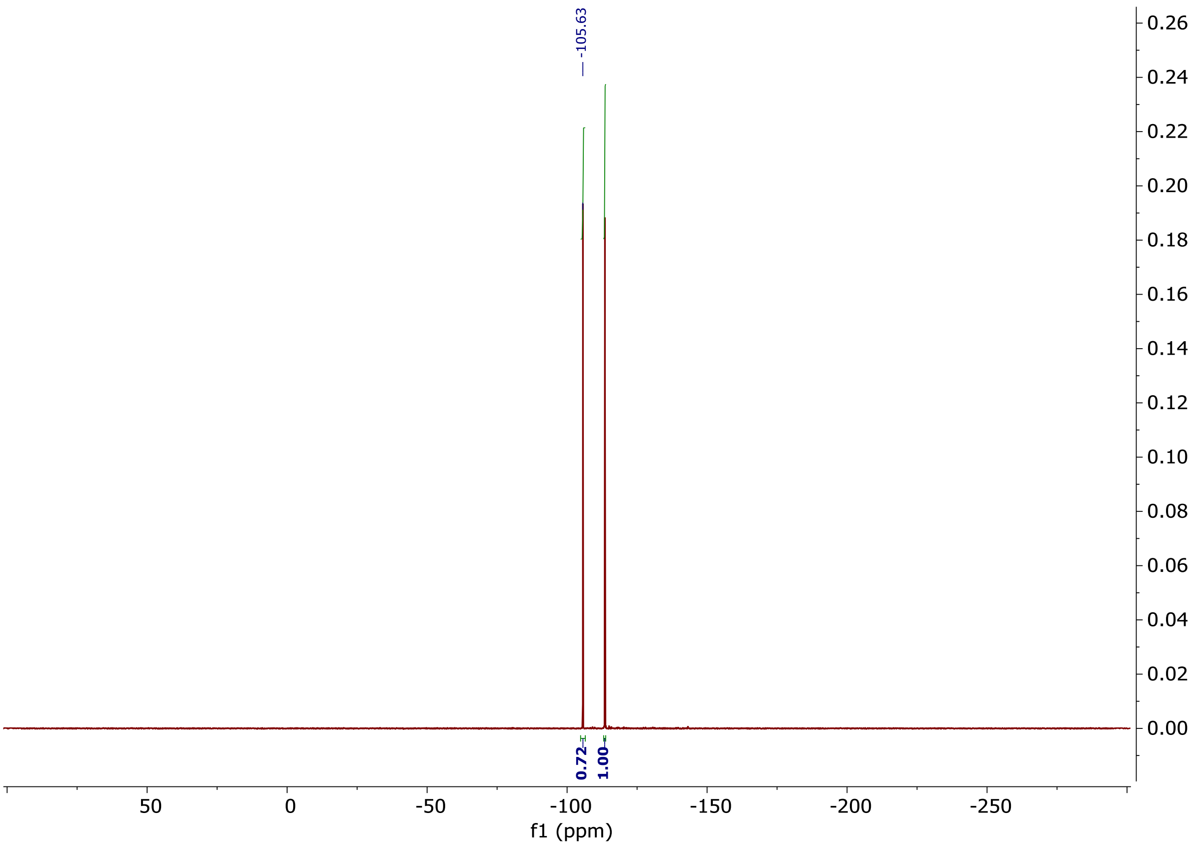

Figure S8.14. ^{19}F NMR (376 MHz) spectrum of the crude obtained after the 0.06 mmol scale divided cell electrolysis of perfluorodecalin in dry acetone with a Zn cathode (10 *F*) and with 50 µL of fluorobenzene as internal standard. The fluoride (F^-) peak is visible at -105.63 ppm.

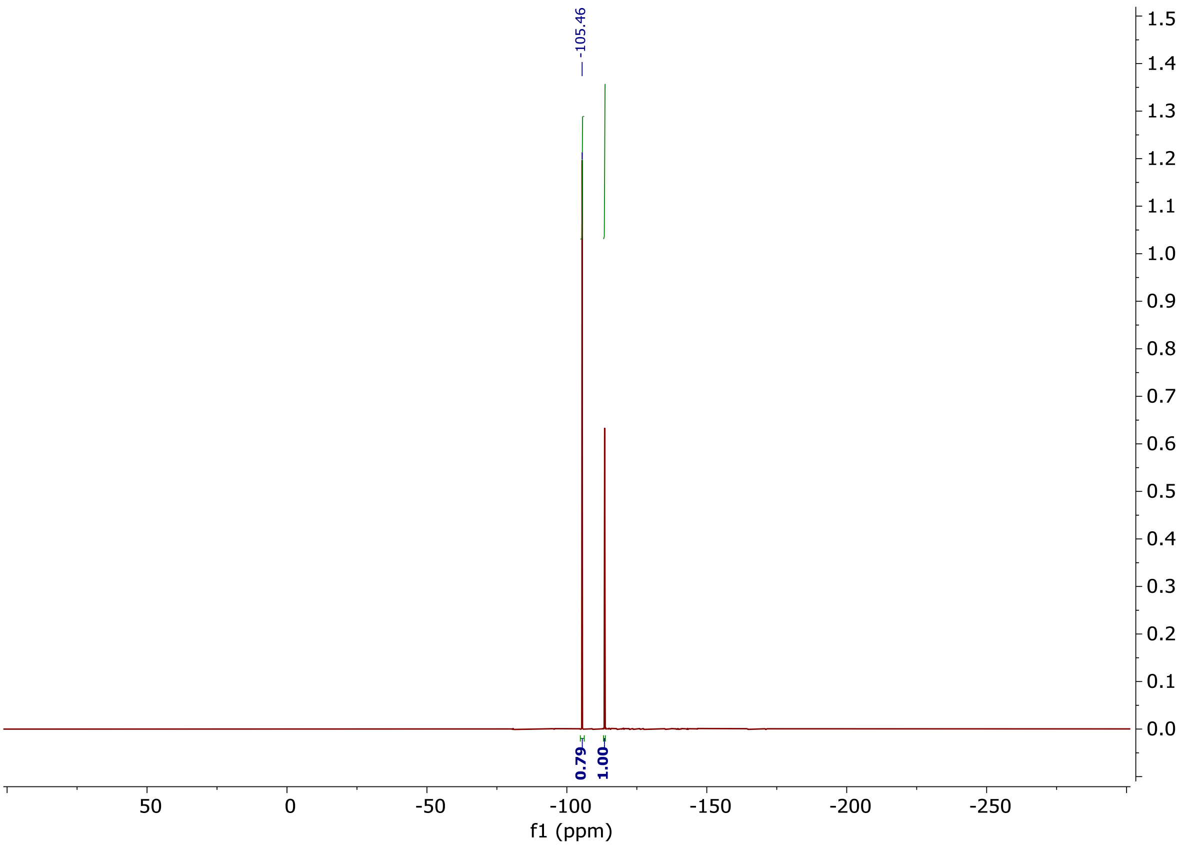

Figure S8.15. ^{19}F NMR (376 MHz) spectrum of the crude obtained after the 0.06 mmol scale divided cell electrolysis of perfluorodecalin in dry acetone with a Ni cathode (10 *F*) and with 50 µL of fluorobenzene as internal standard. The fluoride (F^-) peak is visible at -105.46 ppm.

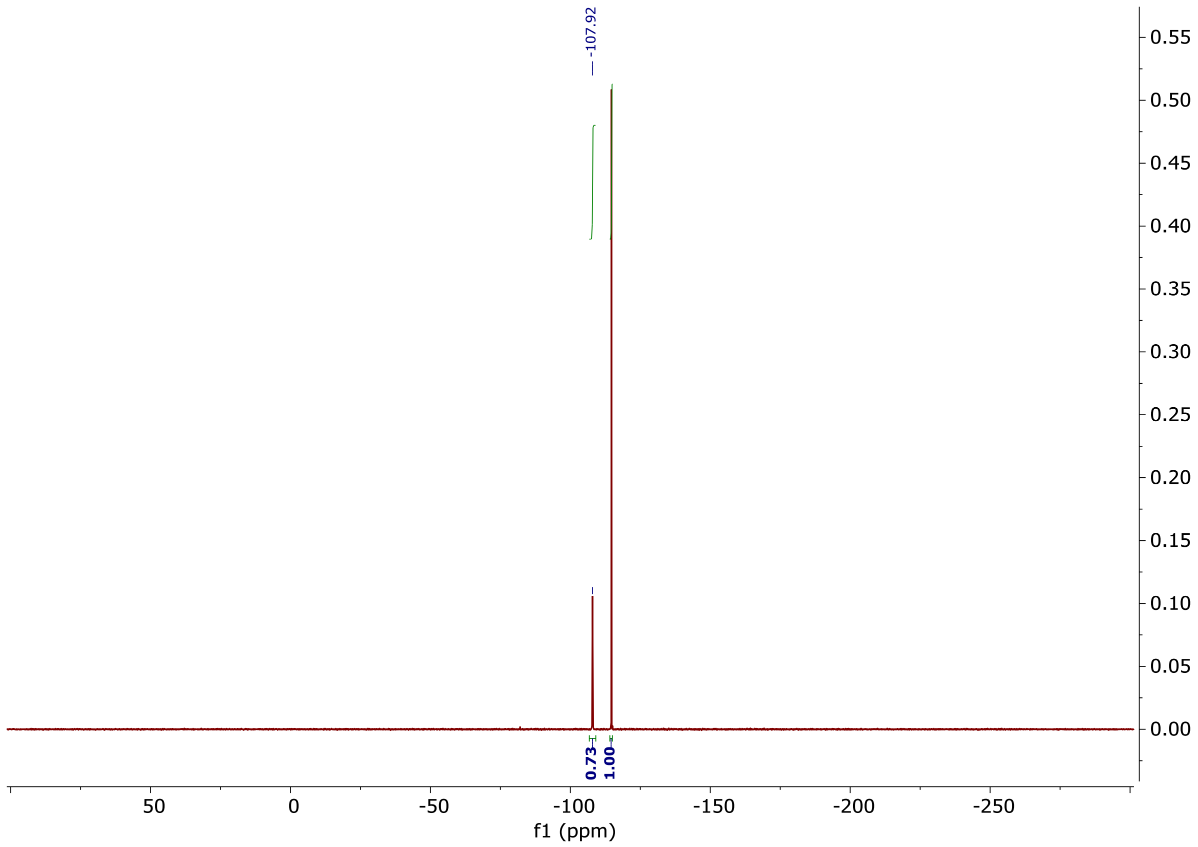

Figure S8.16. ^{19}F NMR (376 MHz) spectrum of the crude obtained after the 0.06 mmol scale divided cell electrolysis of perfluorodecalin in dry MEK (10 *F*) with 50 µL of fluorobenzene as internal standard. The fluoride (F^-) peak is visible at -107.92 ppm.

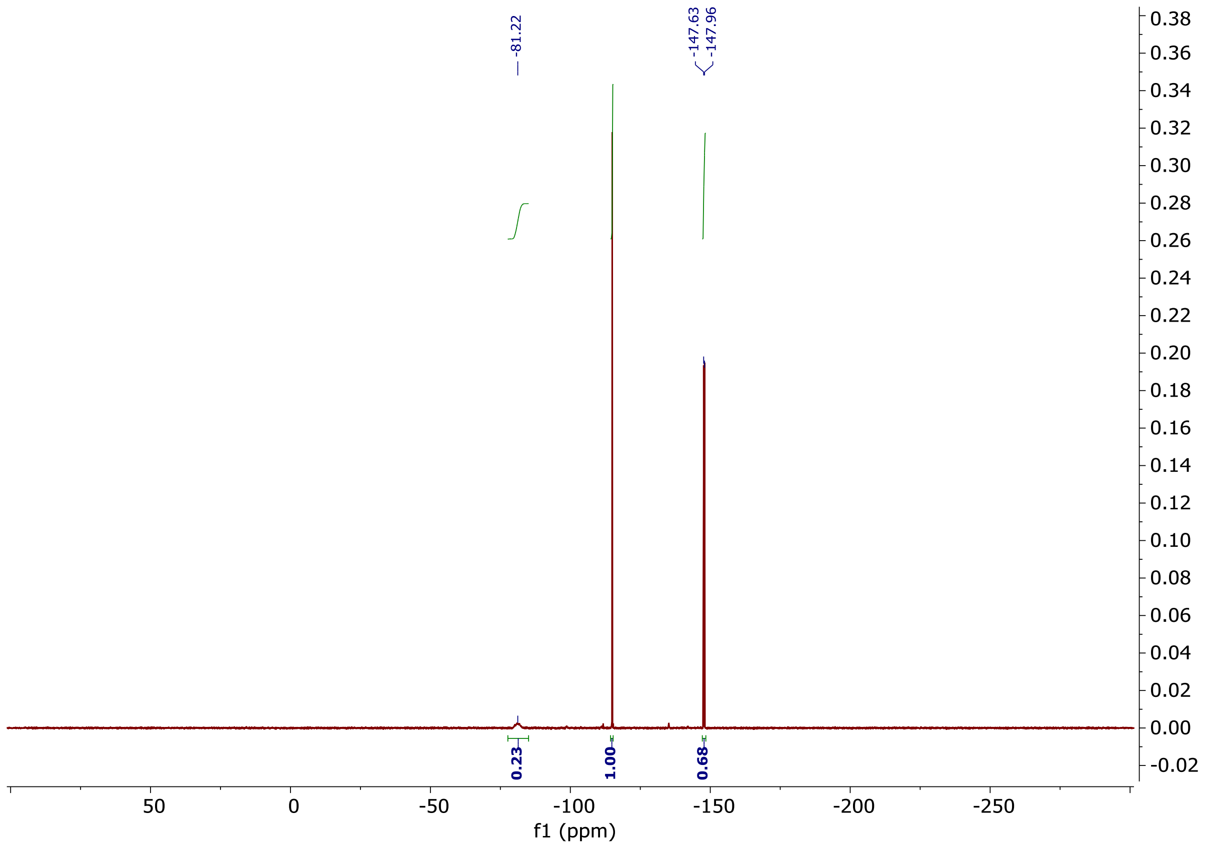

Figure S8.17. ^{19}F NMR (376 MHz) spectrum of the crude obtained after the 0.06 mmol scale divided cell electrolysis of perfluorodecalin in dry acetonitrile (10 *F*) with 50 µL of fluorobenzene as internal standard. The broad and low intensity fluoride (F^-) peak is visible at -81.22 ppm and the bifluoride (HF_2^-) doublet is visible at -147.79 ppm (J=121.4 Hz).

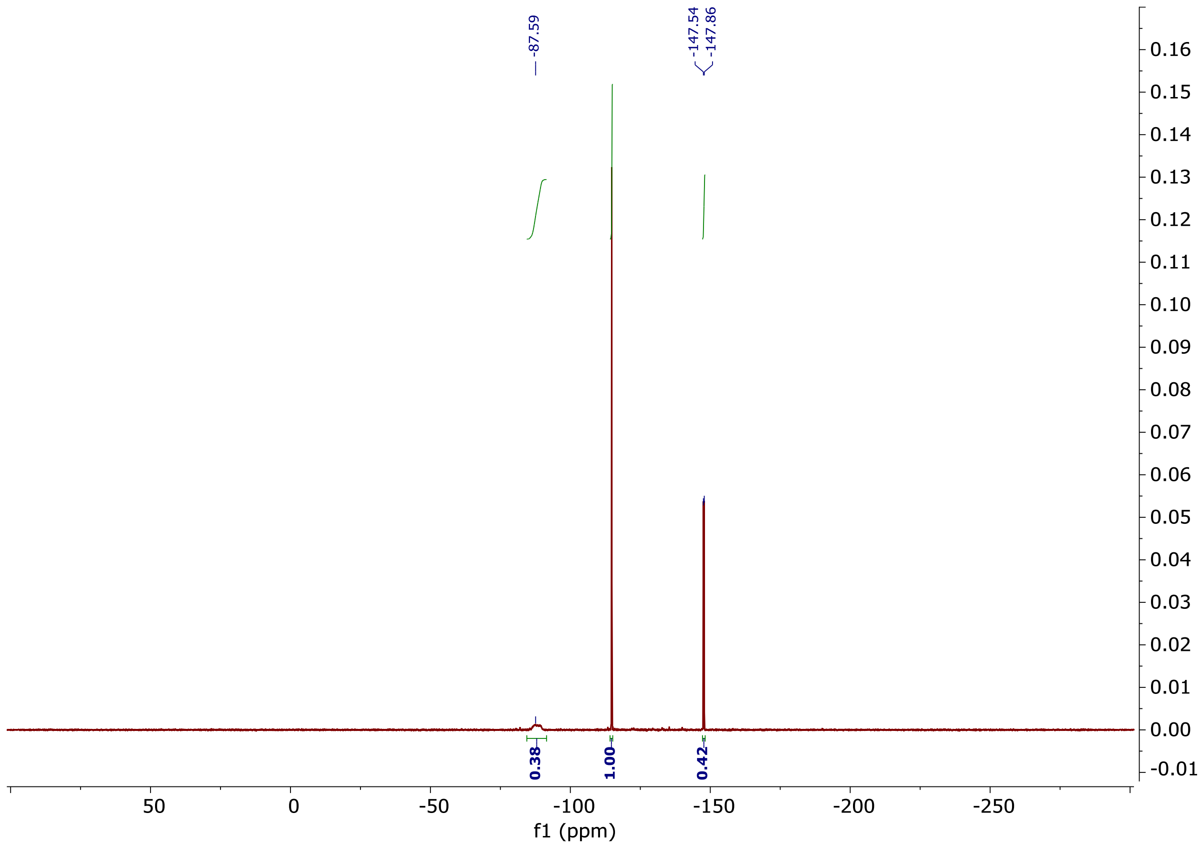

Figure S8.18. ^{19}F NMR (376 MHz) spectrum of the crude obtained after the 0.06 mmol scale divided cell electrolysis of perfluorodecalin in dry DMF (10 *F*) with 50 µL of fluorobenzene as internal standard. The broad and low intensity fluoride (F^-) peak is visible at -87.59 ppm and the bifluoride (HF_2^-) doublet is visible at -147.70 ppm (*J*=119.4 Hz).

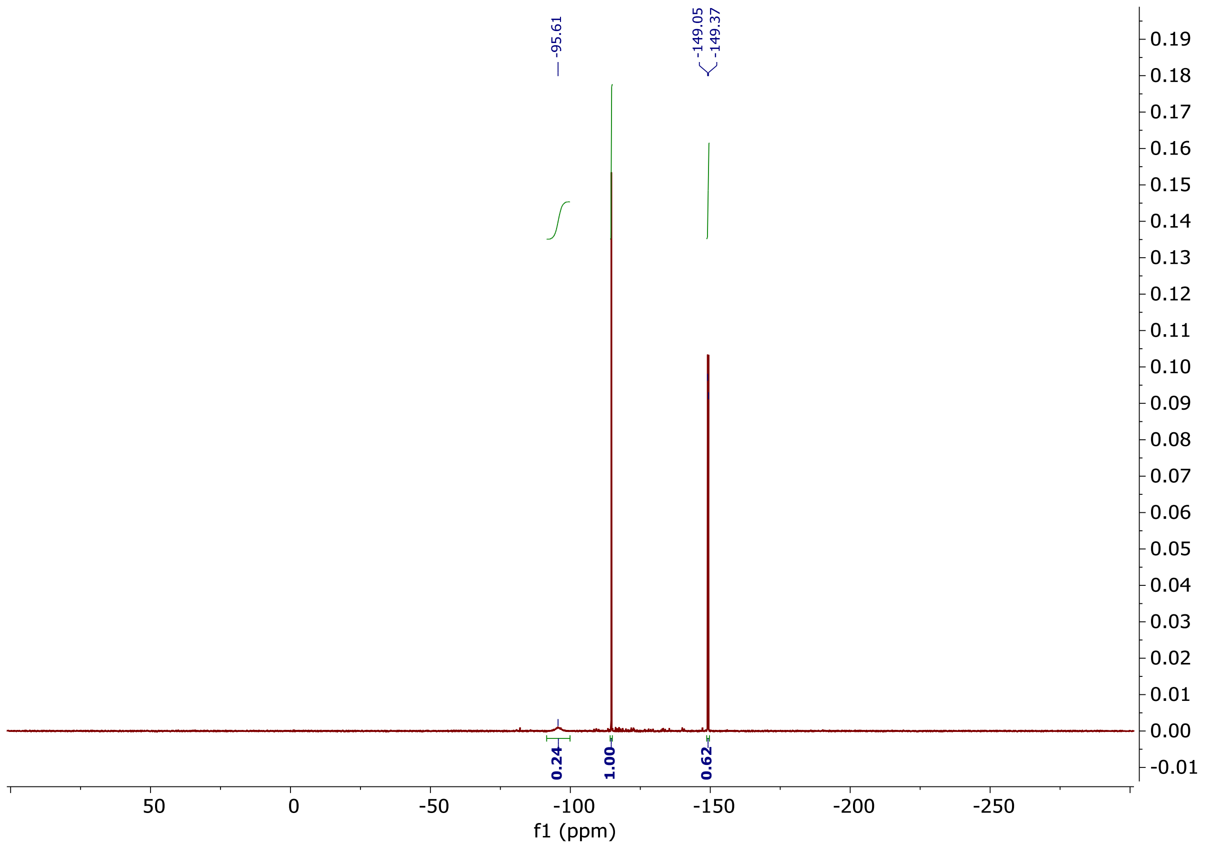

Figure S8.19. ^{19}F NMR (376 MHz) spectrum of the crude obtained after the 0.06 mmol scale divided cell electrolysis of perfluorodecalin in dry DMA (10 *F*) with 50 µL of fluorobenzene as internal standard. The broad and low intensity fluoride (F^-) peak is visible at -95.61 ppm and the bifluoride (HF_2^-) doublet is visible at -149.21 ppm (J=118.7 Hz).

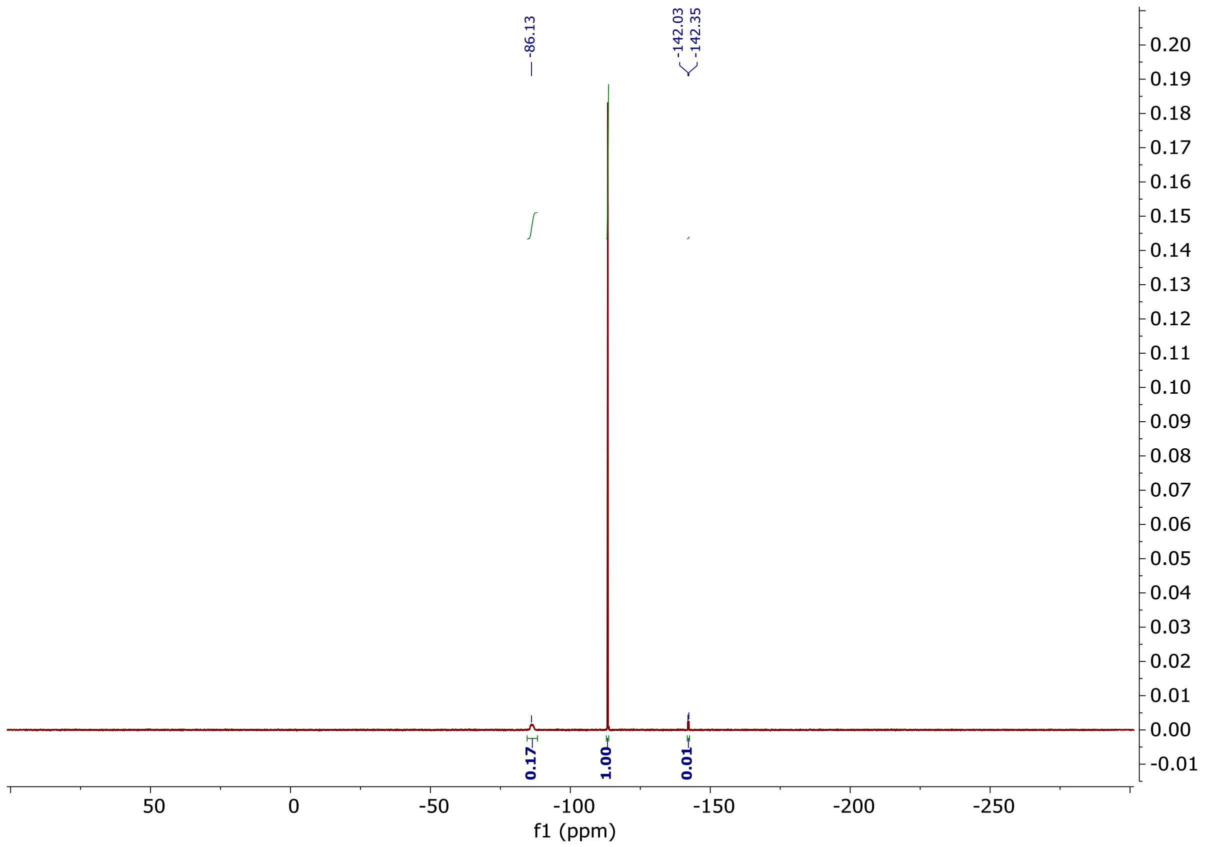

Figure S8.20. ^{19}F NMR (376 MHz) spectrum of the crude obtained after the 0.06 mmol scale divided cell electrolysis of perfluorodecalin in dry DMSO (10 *F*) with 50 µL of fluorobenzene as internal standard. The broad and low intensity fluoride (F^-) peak is visible at -86.13 ppm and the low intensity bifluoride (HF_2^-) doublet is visible at -142.19 ppm (*J*=122.0 Hz).

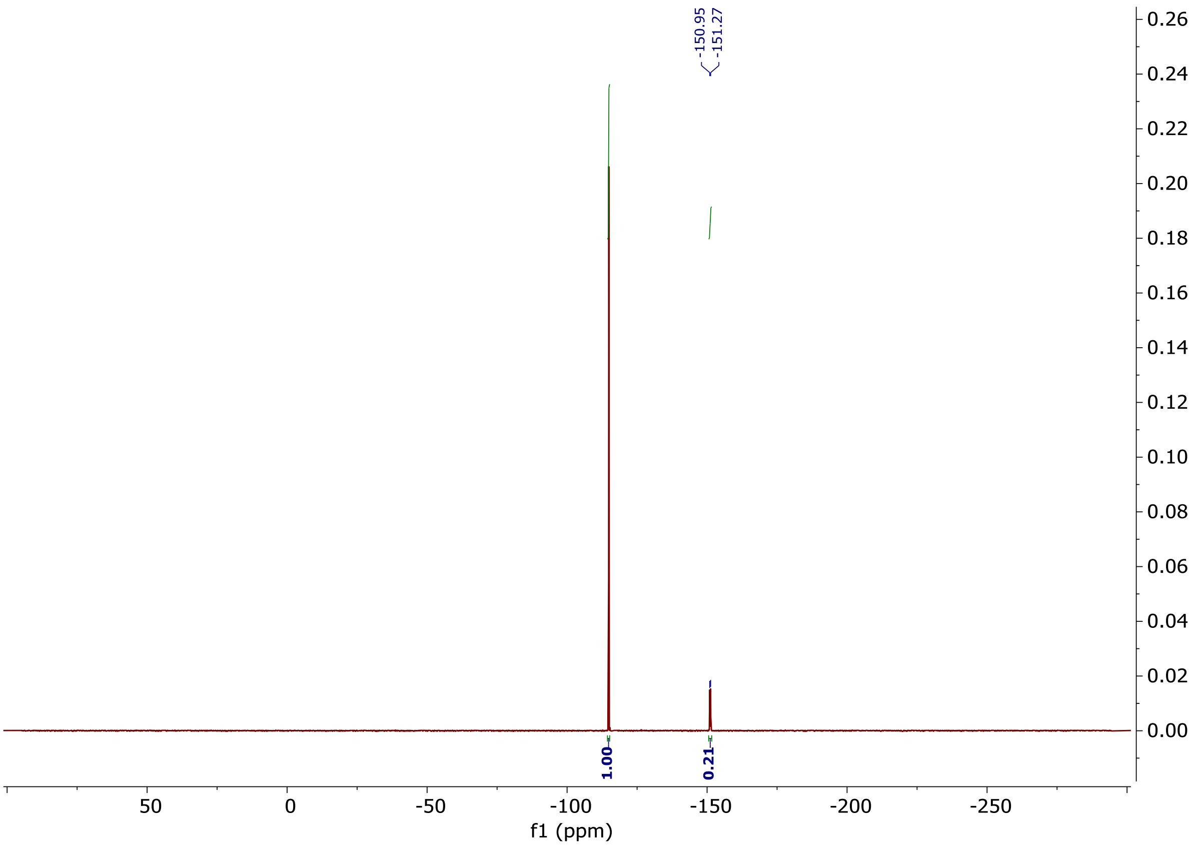

Figure S8.21. ^{19}F NMR (376 MHz) spectrum of the crude obtained after the 0.06 mmol scale divided cell electrolysis of perfluorodecalin in dry THF (10 *F*) with 50 µL of fluorobenzene as internal standard. The bifluoride (HF_2^-) doublet is visible at -151.11 ppm (*J*=119.2 Hz).

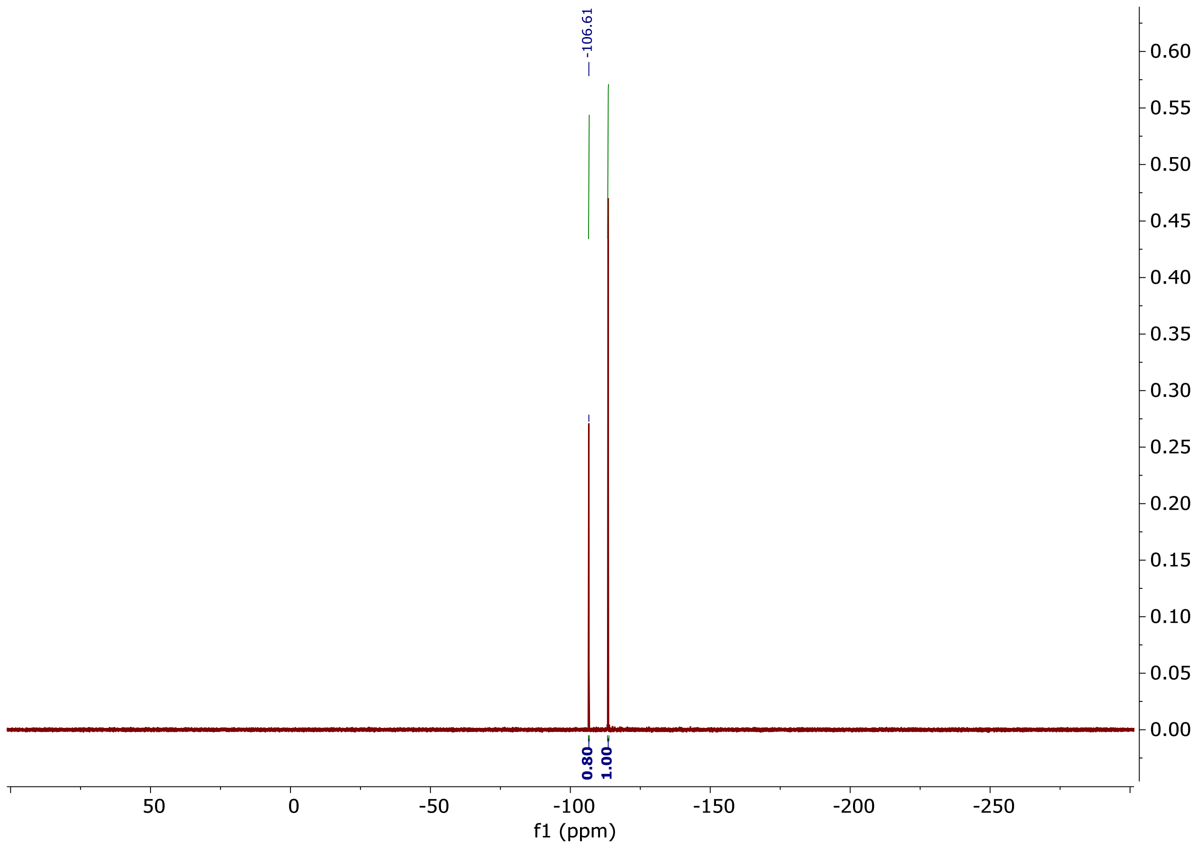

Figure S8.22. ^{19}F NMR (376 MHz) spectrum of the crude obtained after the 0.06 mmol scale divided cell electrolysis of perfluorodecalin in dry acetone with a stainless steel cathode (18 *F*) and with 50 µL of fluorobenzene as internal standard. The fluoride (F^-) peak is visible at -106.61 ppm.

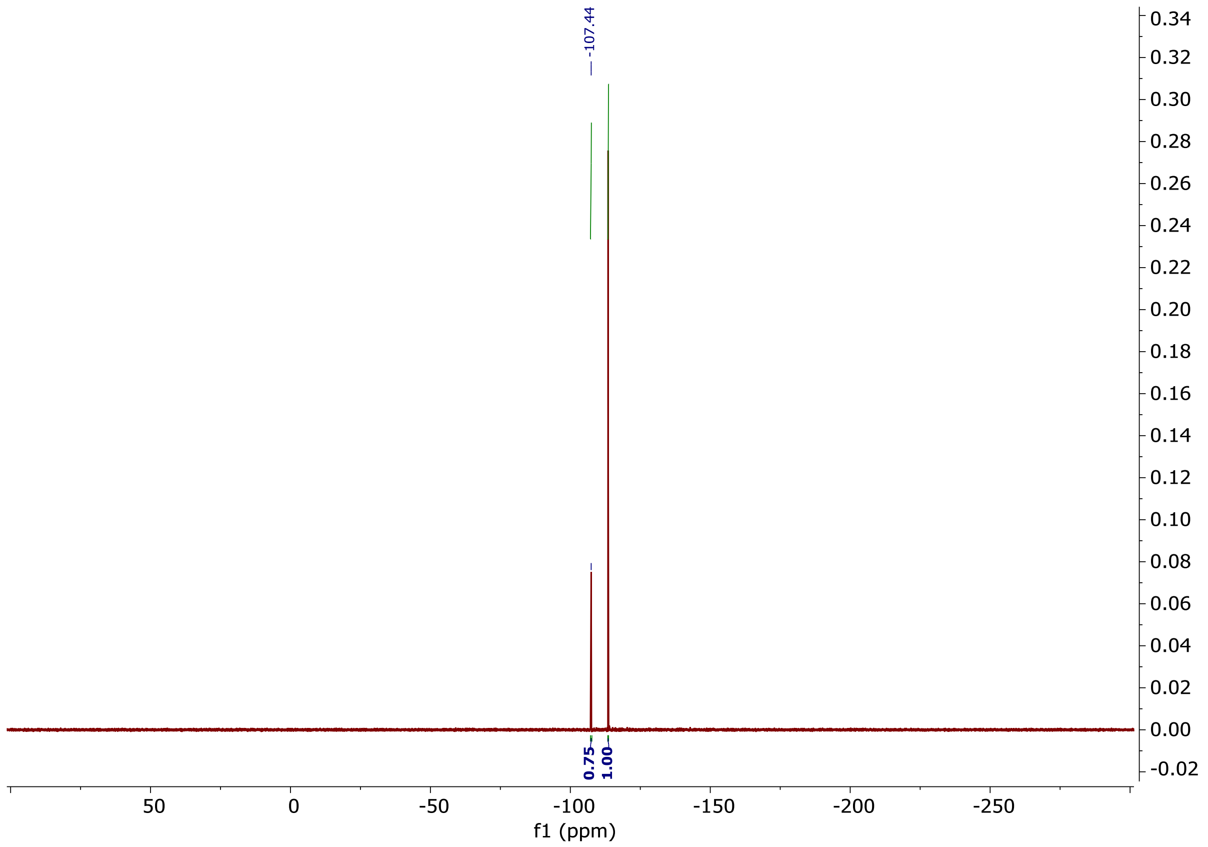

Figure S8.23. ^{19}F NMR (376 MHz) spectrum of the crude obtained after the 0.06 mmol scale divided cell electrolysis of perfluorodecalin in dry acetone with a stainless steel cathode (22 *F*) and with 50 µL of fluorobenzene as internal standard. The fluoride (F^-) peak is visible at -107.44 ppm.

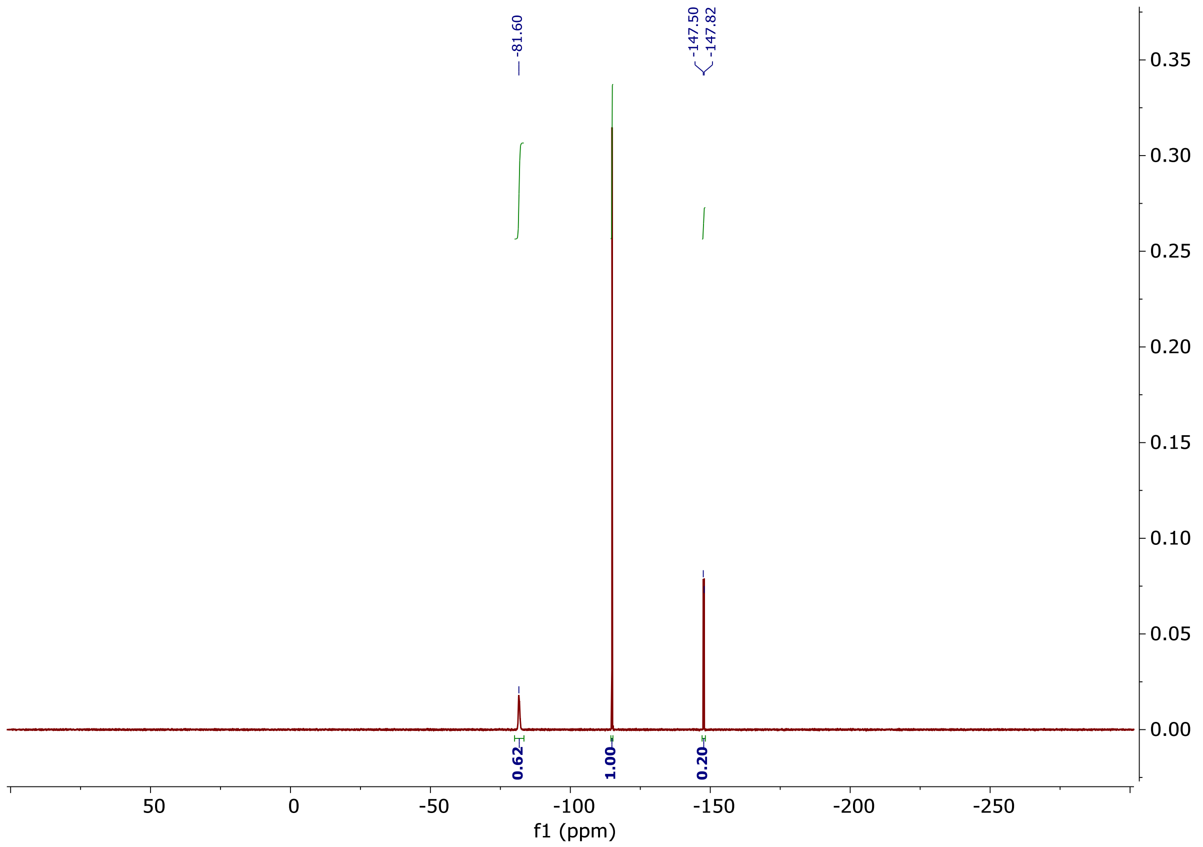

Figure S8.24. ^{19}F NMR (376 MHz) spectrum of the crude obtained after the 0.06 mmol scale divided cell electrolysis of perfluorodecalin in dry acetonitrile (18 *F*) with 50 µL of fluorobenzene as internal standard. The fluoride (F^-) peak is visible at -81.60 ppm and the bifluoride (HF_2^-) doublet is visible at -147.66 ppm (J=120.2 Hz).

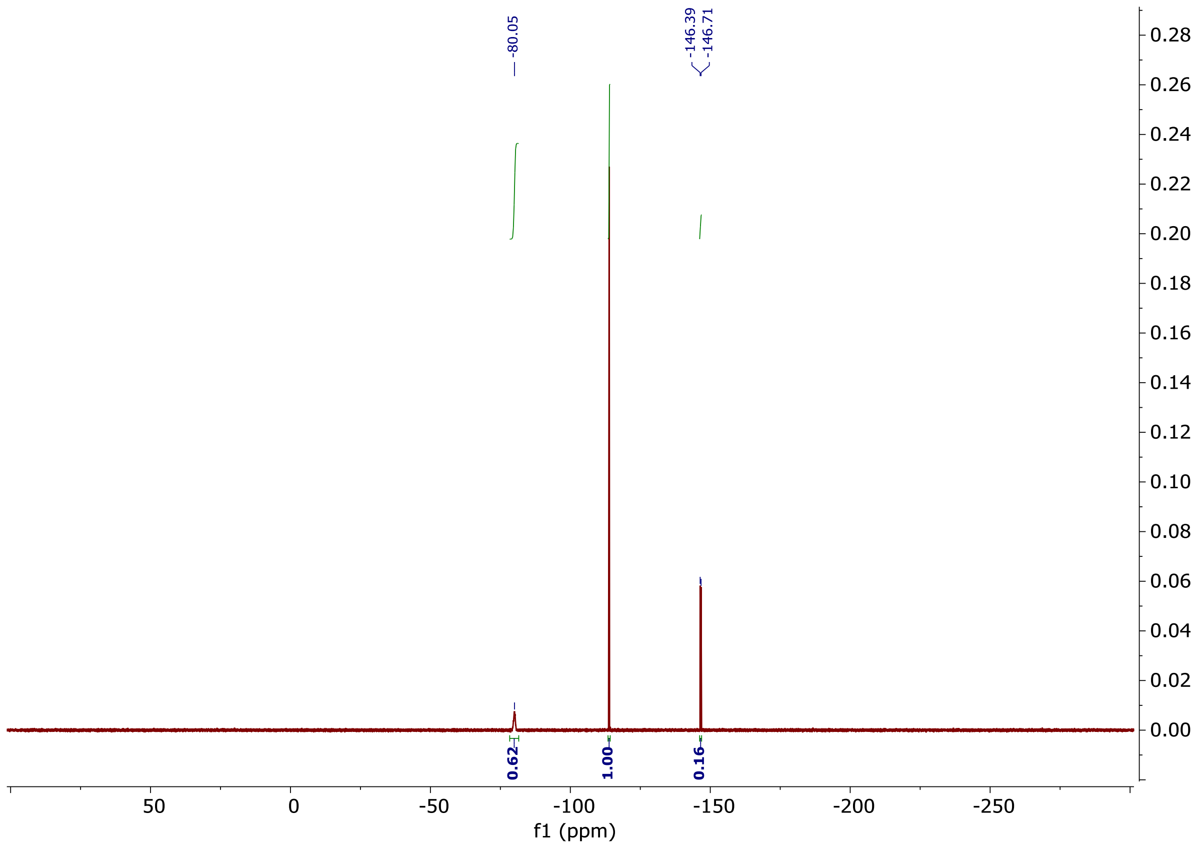

Figure S8.25. ^{19}F NMR (376 MHz) spectrum of the crude obtained after the 0.06 mmol scale divided cell electrolysis of perfluorodecalin in dry acetonitrile (22 *F*) with 50 µL of fluorobenzene as internal standard. The fluoride (F^-) peak is visible at -80.05 ppm and the bifluoride (HF_2^-) doublet is visible at -146.55 ppm (*J*=121.2 Hz).

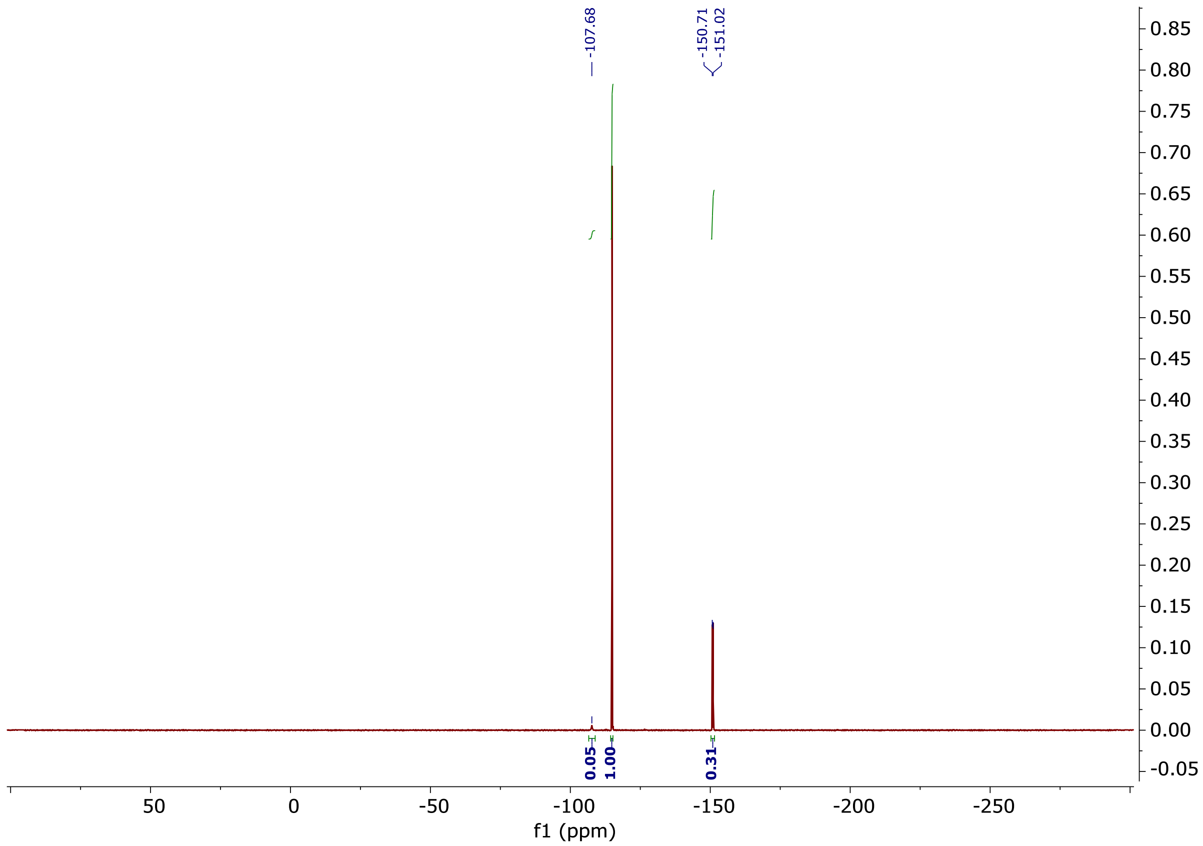

Figure S8.26. ^{19}F NMR (376 MHz) spectrum of a crude obtained after a 0.06 mmol scale divided cell electrolysis of perfluorodecalin in dry THF (18 *F*) with 50 µL of fluorobenzene as internal standard. The broad and low intensity fluoride (F^-) peak is visible at -107.68 ppm and the bifluoride (HF_2^-) doublet is visible at -150.86 ppm (*J*=119.0 Hz). In this electrolysis, the selectivity was in favor of bifluoride, which is not always the case (see below).

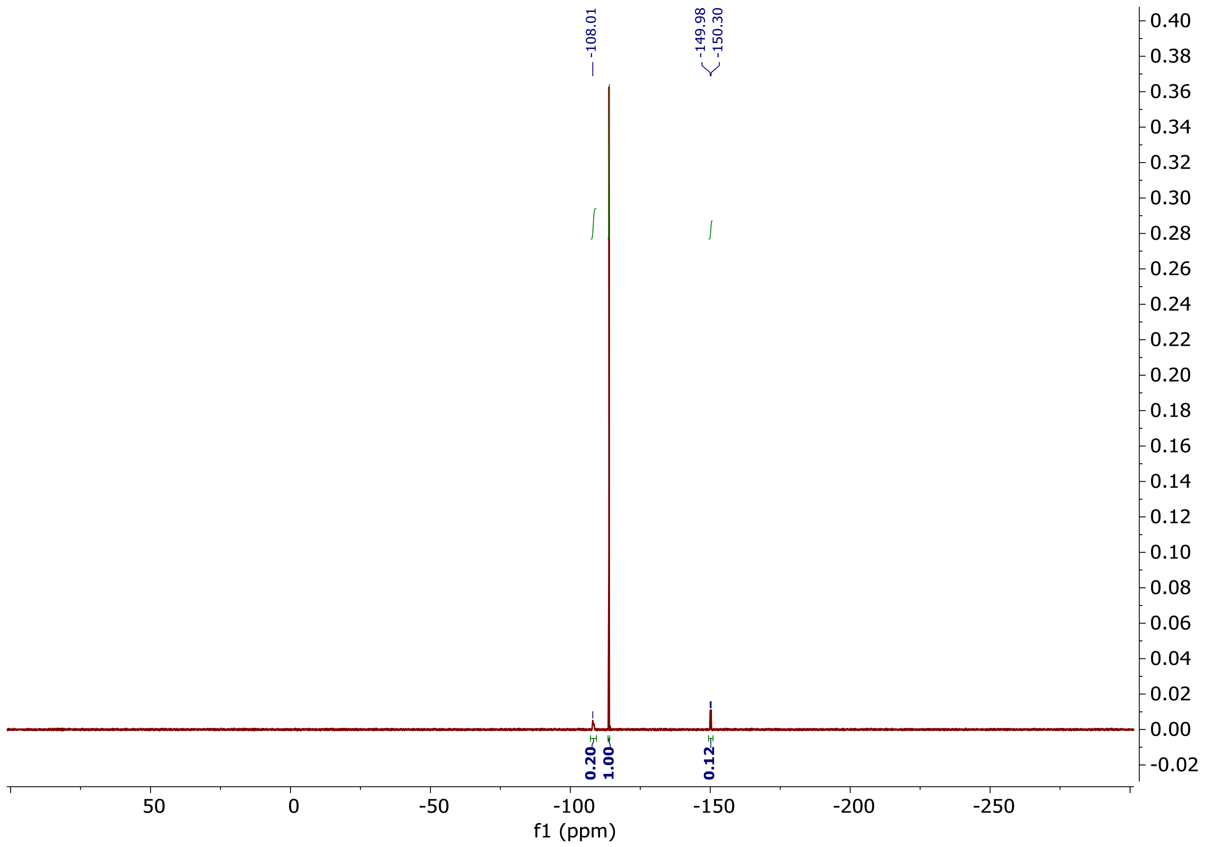

Figure S8.27. ^{19}F NMR (376 MHz) spectrum of another crude obtained after a 0.06 mmol scale divided cell electrolysis of perfluorodecalin in dry THF (18 *F*) with 50 µL of fluorobenzene as internal standard. The broad and low intensity fluoride (F^-) peak is visible at -108.01 ppm and the bifluoride (HF_2^-) doublet is visible at -150.14 ppm (*J*=120.3 Hz). This time, the selectivity was in favor of fluoride, showing the variability of selectivity obtained in divided cell electrolysis with a charge passed of 18 *F* in THF with perfluorodecalin as substrate.

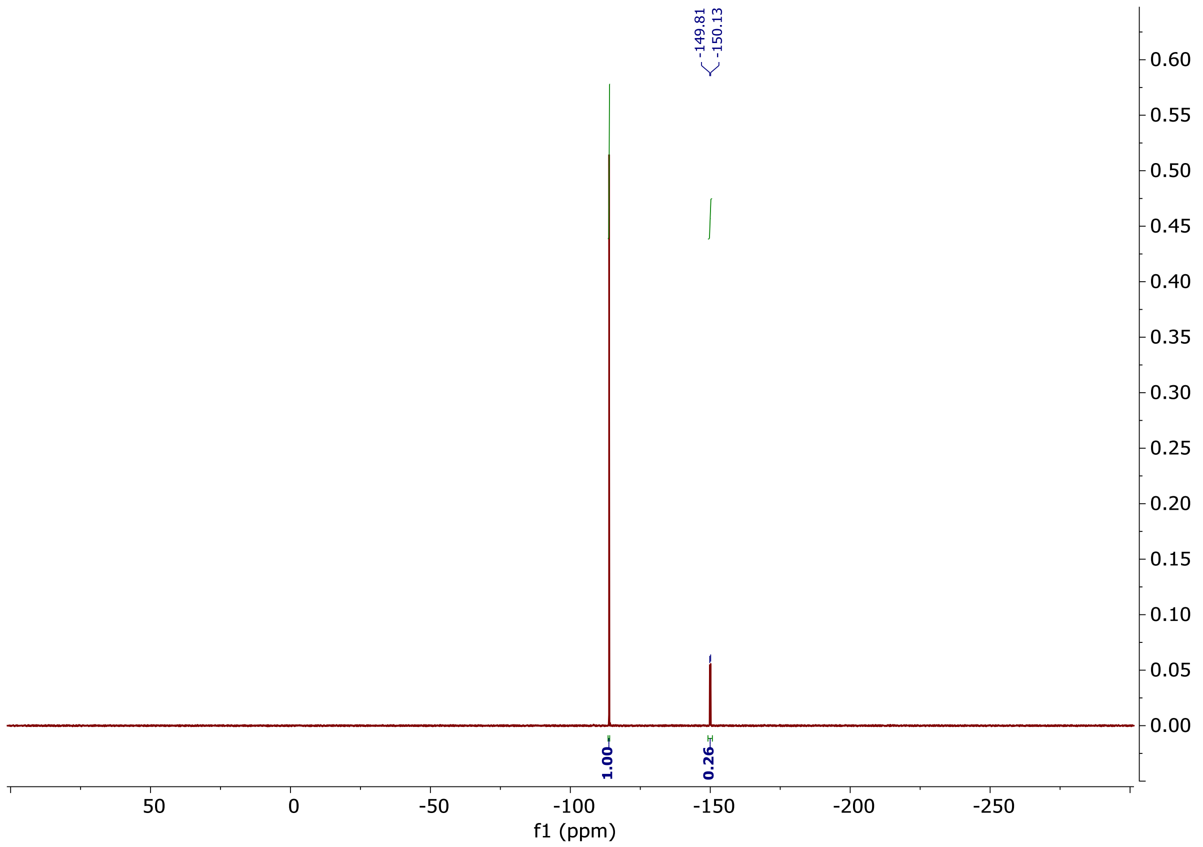

Figure S8.28. ^{19}F NMR (376 MHz) spectrum the crude obtained after the 0.06 mmol scale divided cell electrolysis of perfluorodecalin in dry THF (22 *F*) with 50 µL of fluorobenzene as internal standard. The bifluoride (HF_2^-) doublet is visible at -149.97 ppm (J=120.0 Hz).

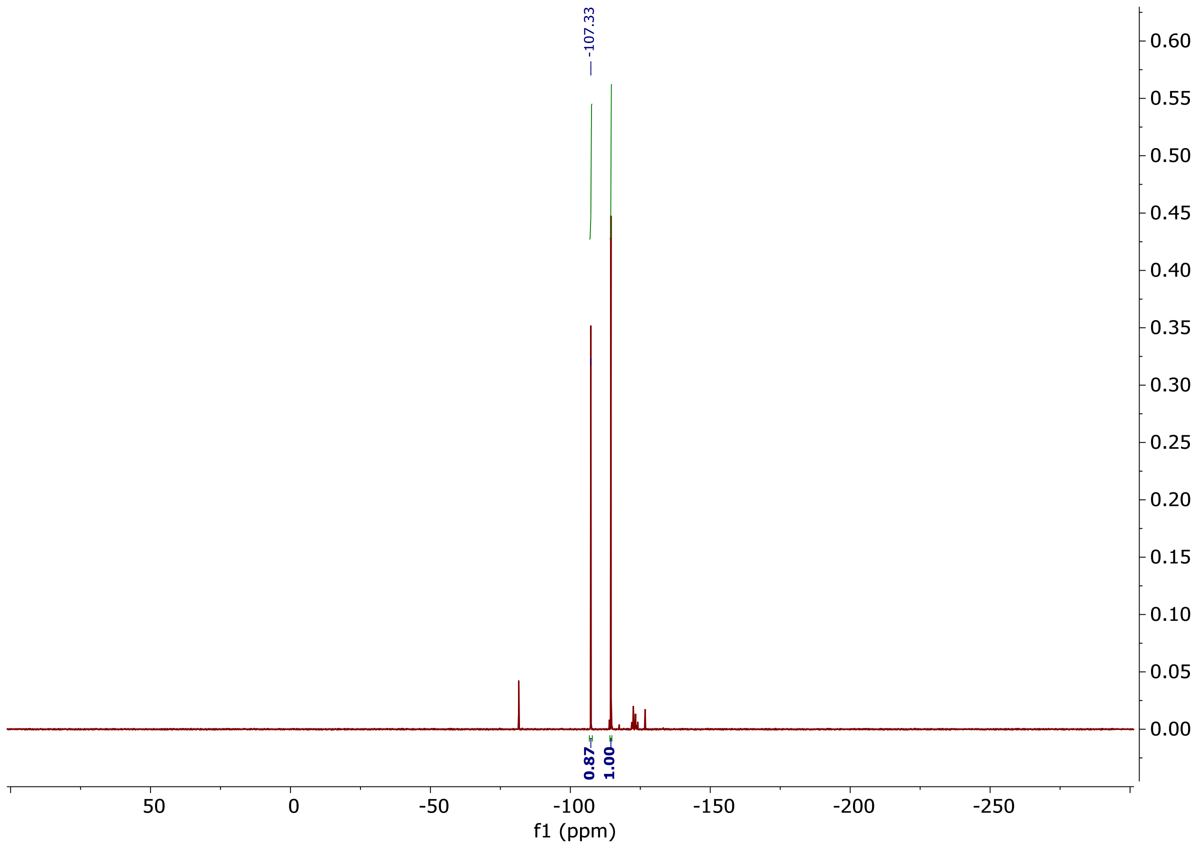

Figure S8.29. ^{19}F NMR (376 MHz) spectrum of the crude obtained after the 0.06 mmol scale divided cell electrolysis of 1H,1H,2H-perfluoro-1-decene in dry acetone (17 *F*) with 50 µL of fluorobenzene as internal standard. The fluoride (F^-) peak is visible at -107.33 ppm. Remaining peaks of 1H,1H,2H-perfluoro-1-decene are visible.

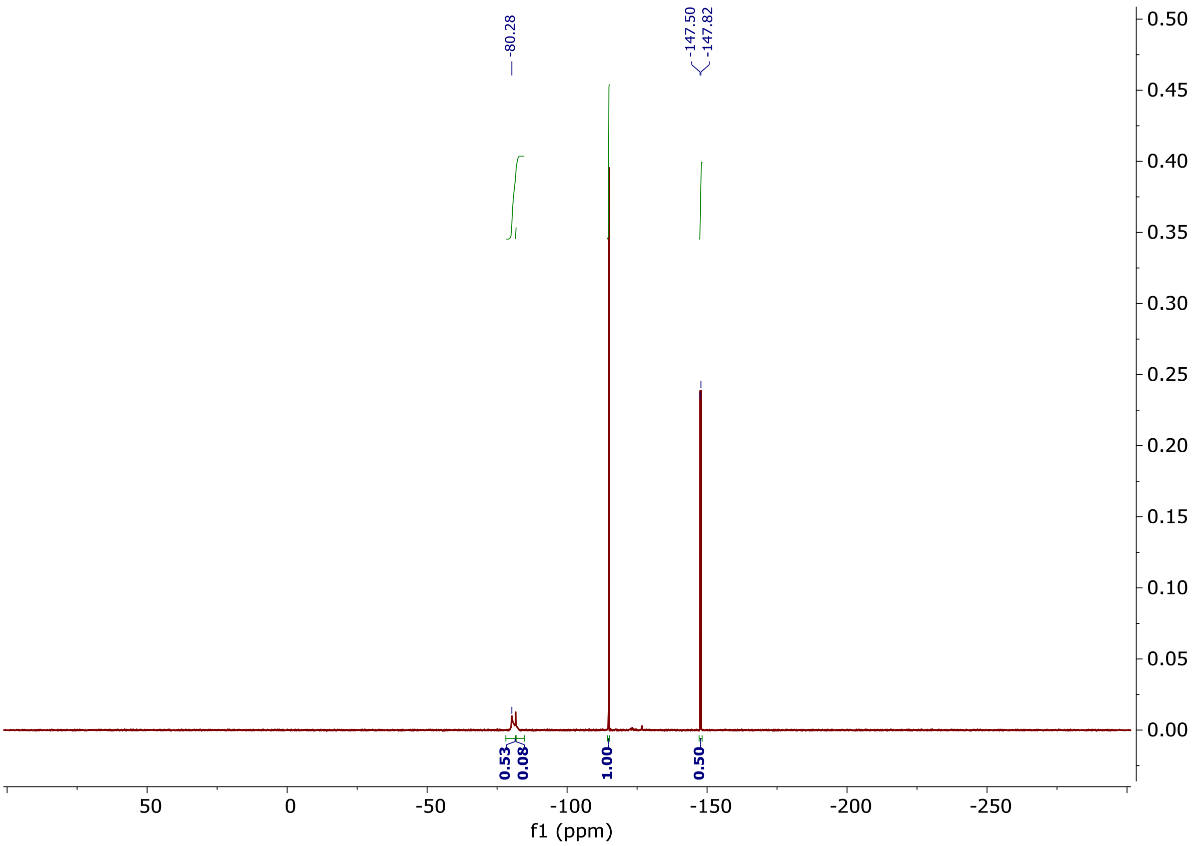

Figure S8.30. ^{19}F NMR (376 MHz) spectrum of the crude obtained after the 0.06 mmol scale divided cell electrolysis of 1H,1H,2H-perfluoro-1-decene in dry acetonitrile (17 *F*) with 50 µL of fluorobenzene as internal standard. The broad fluoride (F^-) peak is visible at -80.28 ppm and the bifluoride (HF_2^-) doublet is visible at -147.66 ppm (*J*=122.0 Hz). Small peaks likely associated to smaller fluorinated chains from 1H,1H,2H-perfluoro-1-decene structure are visible, including one at -81.63 ppm whose integration has been removed from the integration of the F^- peak.

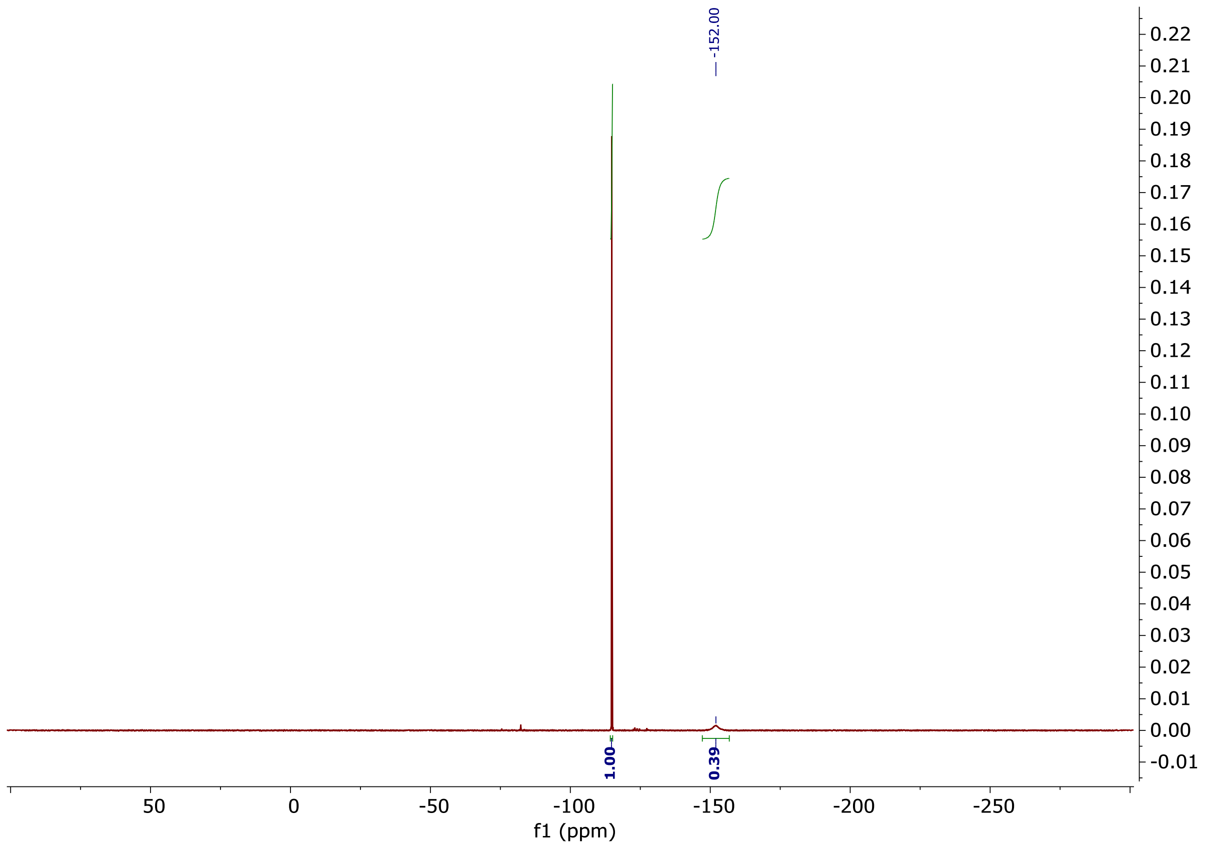

Figure S8.31. ^{19}F NMR (376 MHz) spectrum of the crude obtained after the 0.06 mmol scale divided cell electrolysis of 1H,1H,2H-perfluoro-1-decene in dry THF (17 *F*) with 50 µL of fluorobenzene as internal standard. The bifluoride (HF_2^-) signal appears at -152.00 ppm as a very broad peak on this spectrum.

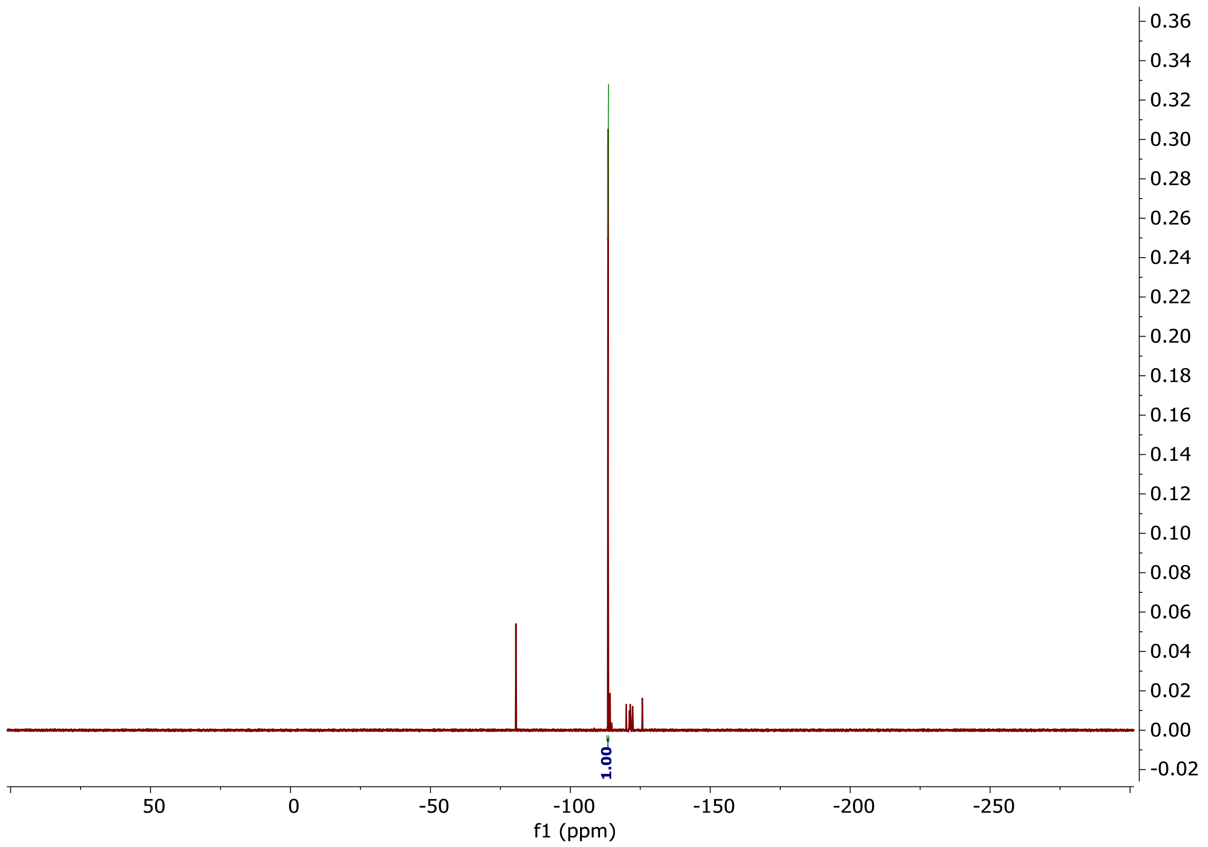

Figure S8.32. ^{19}F NMR (376 MHz) spectrum of the crude obtained after the 0.06 mmol scale divided cell electrolysis of PFOS-TBA in dry acetone (17 *F*) with 50 µL of fluorobenzene as internal standard. The electrolysis was unsuccessful: no fluoride (F^-) or bifluoride (HF_2^-) peaks are visible and unaltered peaks of PFOS-TBA are present.

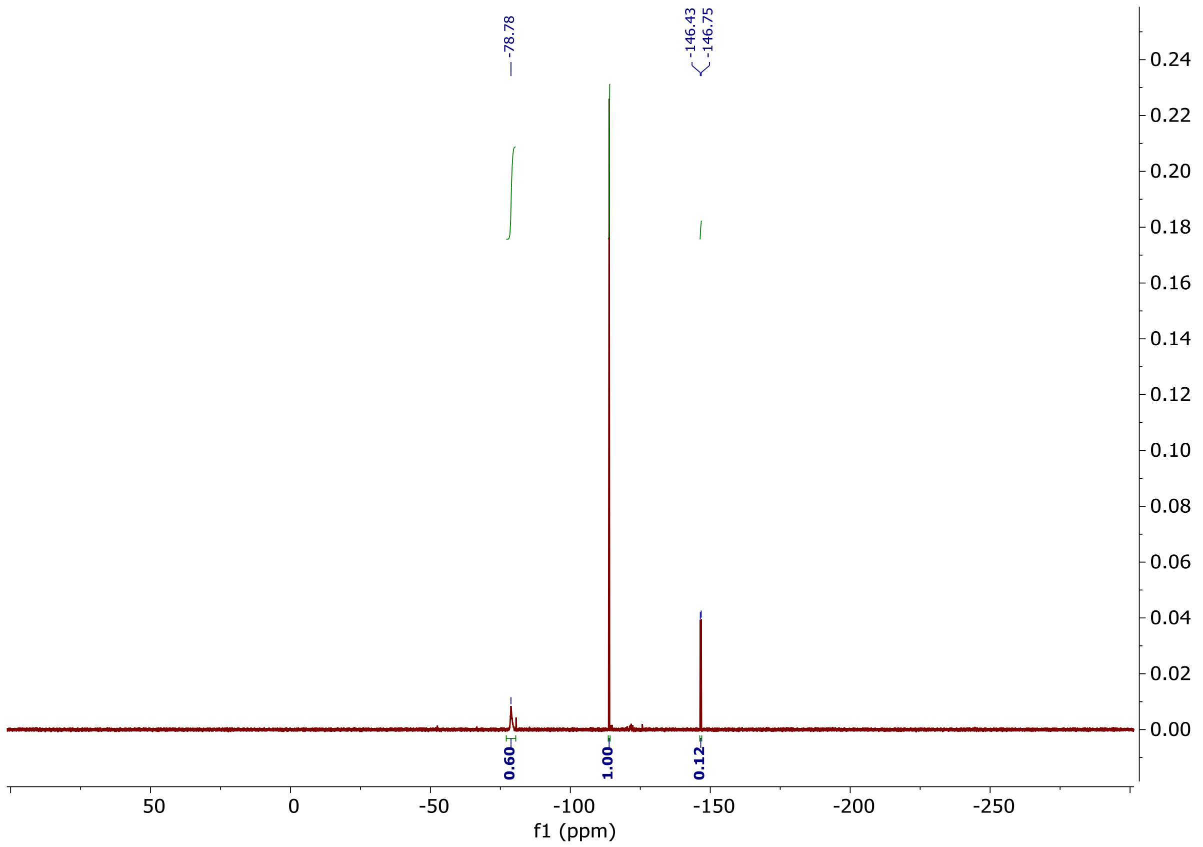

Figure S8.33. ^{19}F NMR (376 MHz) spectrum of the crude obtained after the 0.06 mmol scale divided cell electrolysis of PFOS-TBA in dry acetonitrile (17 *F*) with 50 µL of fluorobenzene as internal standard. The broad fluoride (F^-) peak is visible at -78.78 ppm and the bifluoride (HF_2^-) doublet is visible at -146.59 ppm (*J*=121.2 Hz). Small peaks likely associated to smaller fluorinated chains from PFOS-TBA structure are visible.

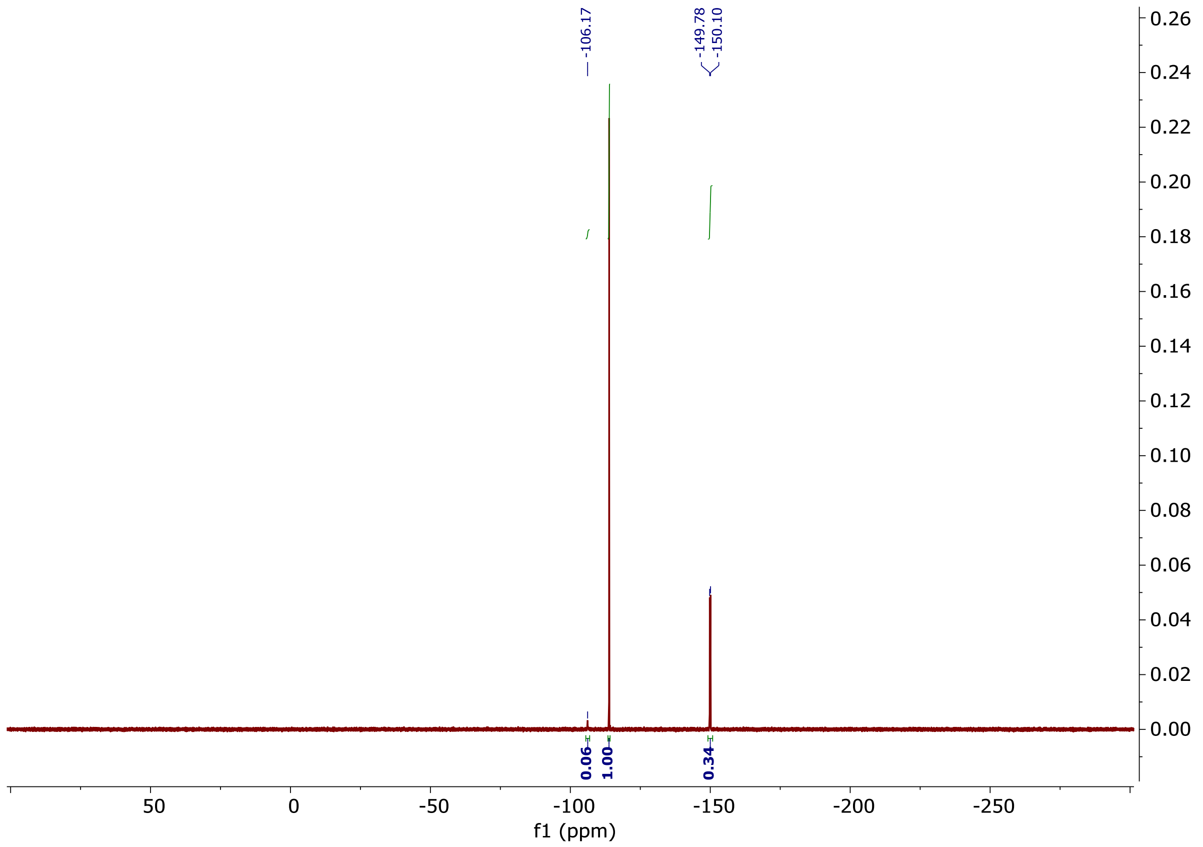

Figure S8.34. ^{19}F NMR (376 MHz) spectrum of the crude obtained after the 0.06 mmol scale divided cell electrolysis of PFOS-TBA in dry THF (17 *F*) with 50 µL of fluorobenzene as internal standard. The broad and low intensity fluoride (F^-) peak is visible at -106.17 ppm and the bifluoride (HF_2^-) doublet is visible at -149.94 ppm (J=119.6 Hz).

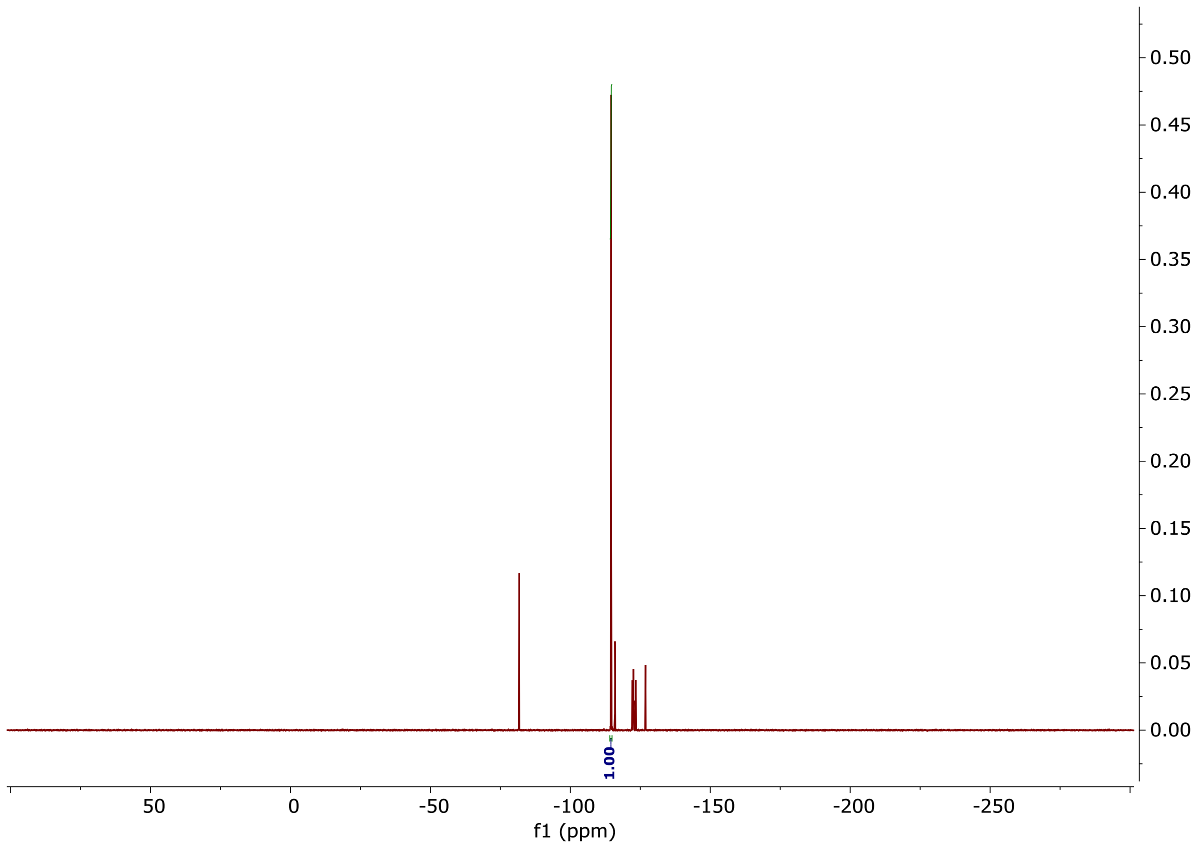

Figure S8.35. ^{19}F NMR (376 MHz) spectrum of the crude obtained after the 0.06 mmol scale divided cell electrolysis of PFOA in dry acetone (15 *F*) with 50 µL of fluorobenzene as internal standard. The electrolysis was unsuccessful: no fluoride (F^-) or bifluoride (HF_2^-) peaks are visible and unaltered peaks of PFOA are present.

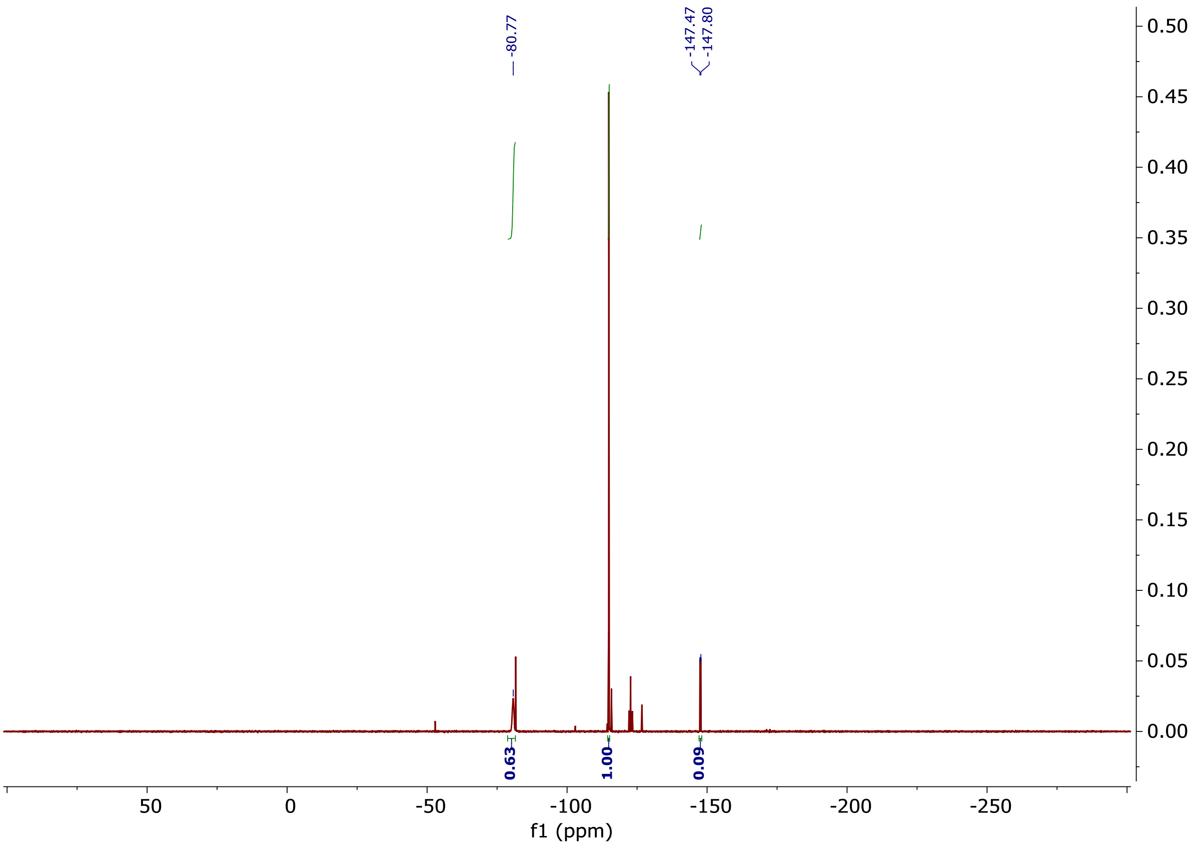

Figure S8.36. ^{19}F NMR (376 MHz) spectrum of the crude obtained after the 0.06 mmol scale divided cell electrolysis of PFOA in dry acetonitrile (15 *F*) with 50 µL of fluorobenzene as internal standard. The broad fluoride (F^-) peak is visible at -80.77 ppm and the bifluoride (HF_2^-) doublet is visible at -147.63 ppm (*J*=121.5 Hz). Peaks likely associated to smaller fluorinated chains from PFOA structure are visible.

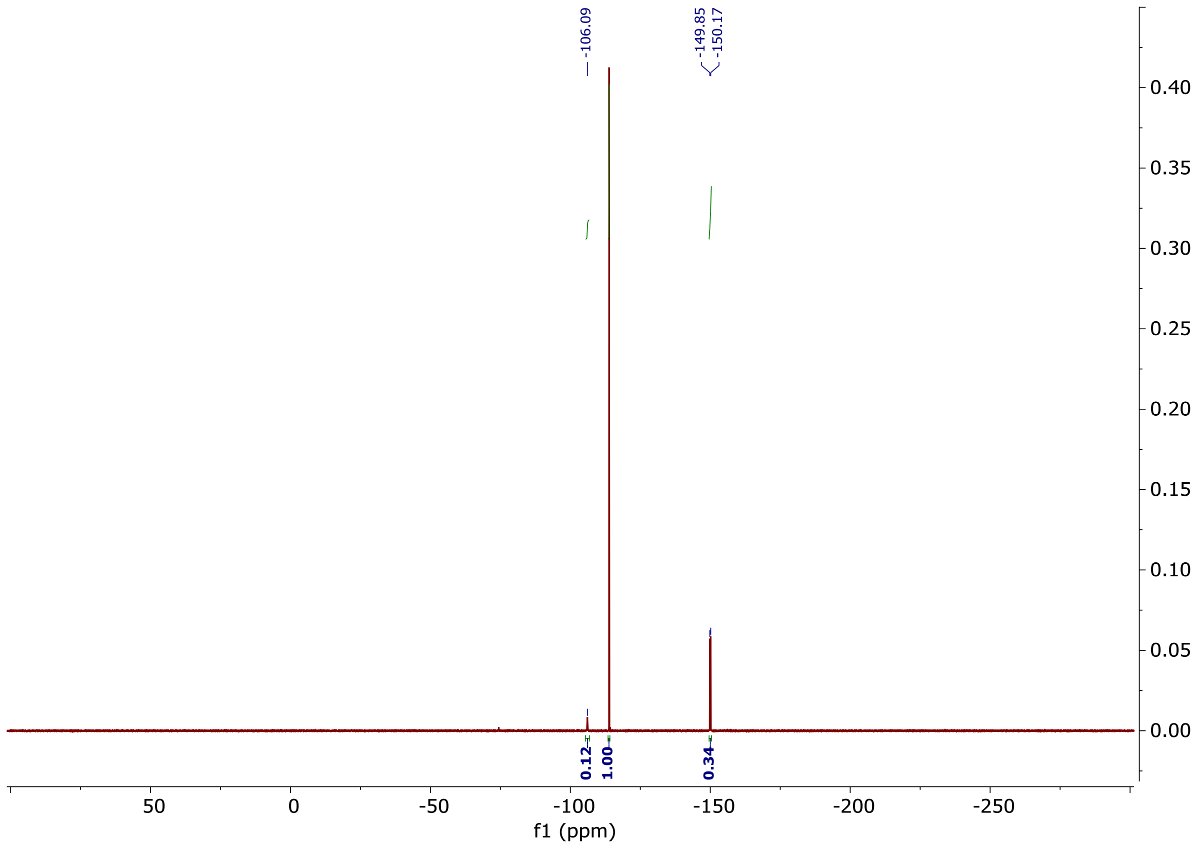

Figure S8.37. ^{19}F NMR (376 MHz) spectrum of the crude obtained after the 0.06 mmol scale divided cell electrolysis of PFOA in dry THF (15 *F*) with 50 µL of fluorobenzene as internal standard. The broad and low intensity fluoride (F^-) peak is visible at -106.09 ppm and the bifluoride (HF_2^-) doublet is visible at -150.01 ppm (*J*=120.0 Hz).

F^-/HF_2^- selectivity – Effect of the presence of water

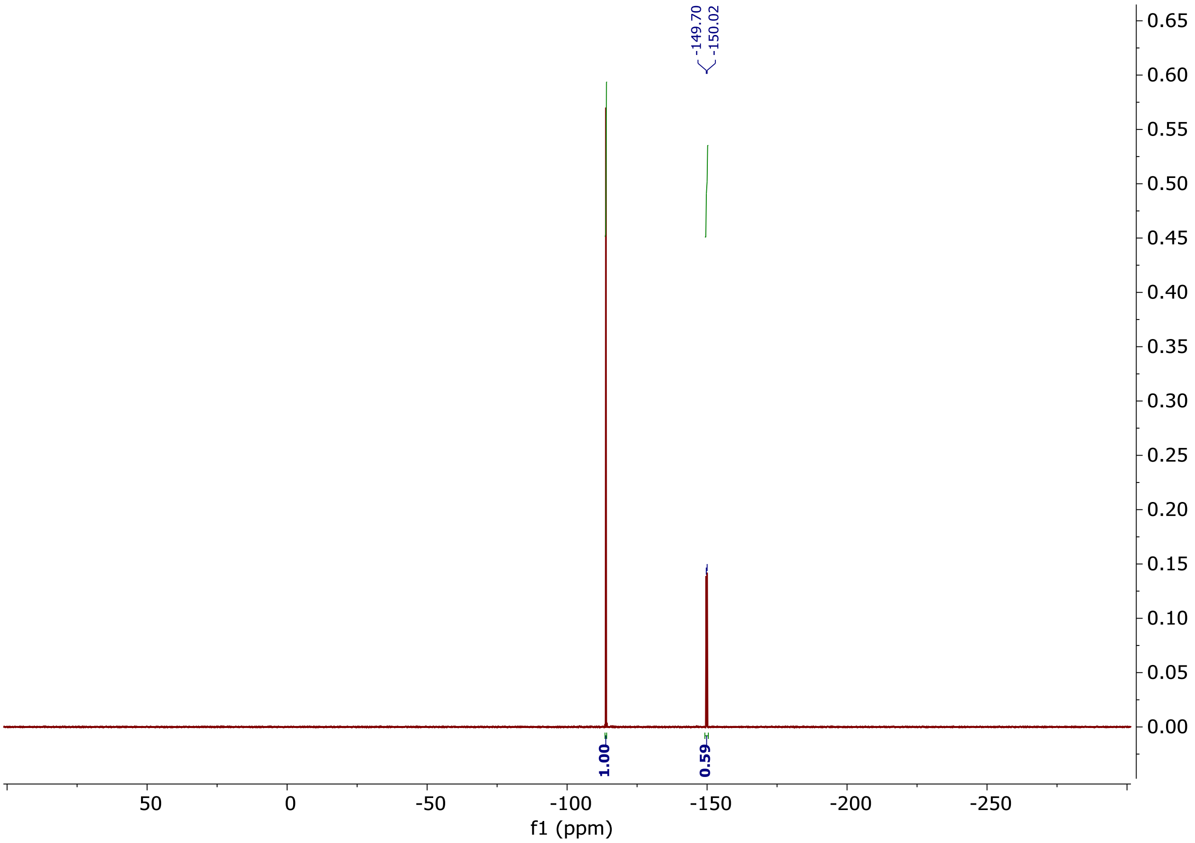

Figure S8.38. ^{19}F NMR (376 MHz) spectrum of the crude obtained after the 0.06 mmol scale divided cell electrolysis of perfluorodecalin in dry THF + 500 ppm H_2O (10 *F*) with 50 µL of fluorobenzene as internal standard. The bifluoride (HF_2^-) doublet is visible at -149.86 ppm (*J*=119.6 Hz).

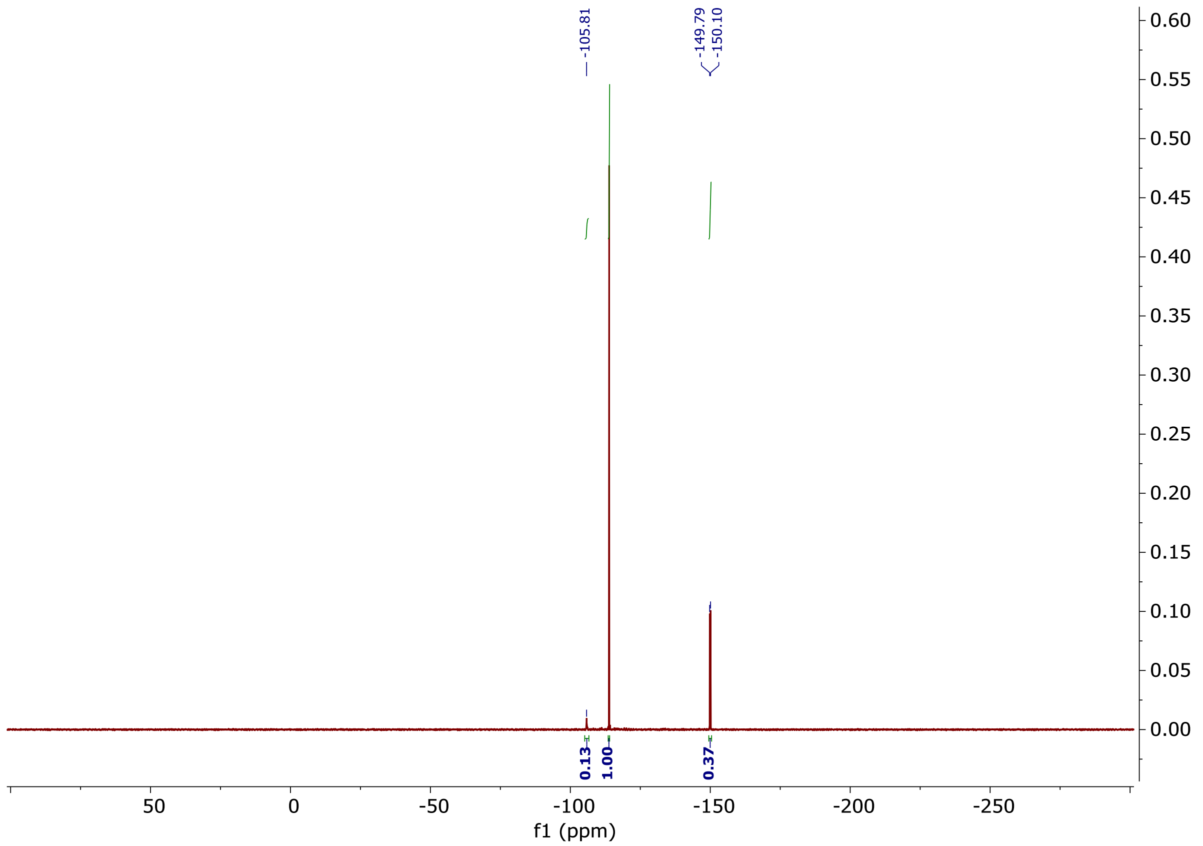

Figure S8.39. ^{19}F NMR (376 MHz) spectrum of the crude obtained after the 0.06 mmol scale divided cell electrolysis of perfluorodecalin in dry THF + 1000 ppm H_2O (10 *F*) with 50 µL of fluorobenzene as internal standard. The fluoride (F^-) peak is visible at -105.81 ppm and the bifluoride (HF_2^-) doublet is visible at -149.95 ppm (*J*=120.0 Hz).

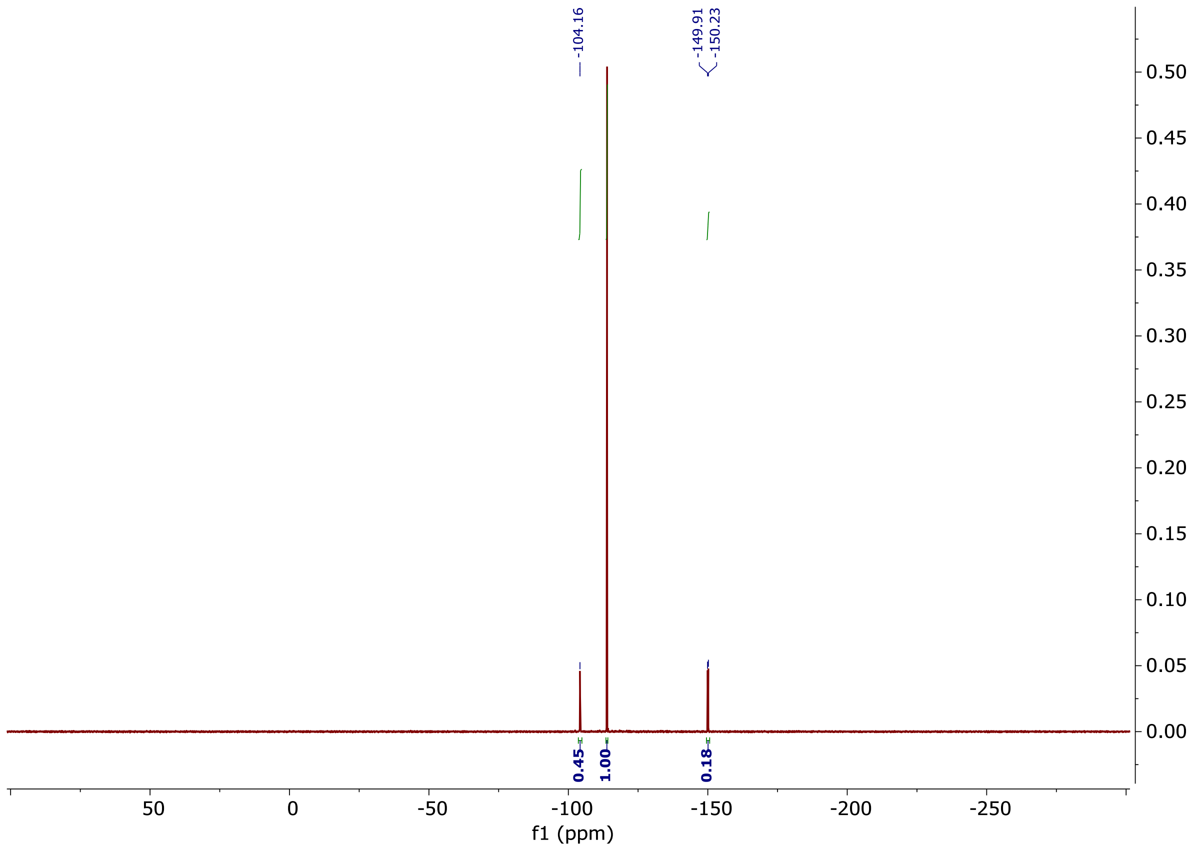

Figure S8.40. ^{19}F NMR (376 MHz) spectrum of the crude obtained after the 0.06 mmol scale divided cell electrolysis of perfluorodecalin in dry THF + 2000 ppm H_2O (10 *F*) with 50 µL of fluorobenzene as internal standard. The fluoride (F^-) peak is visible at -104.16 ppm and the bifluoride (HF_2^-) doublet is visible at -150.07 ppm (*J*=119.8 Hz).

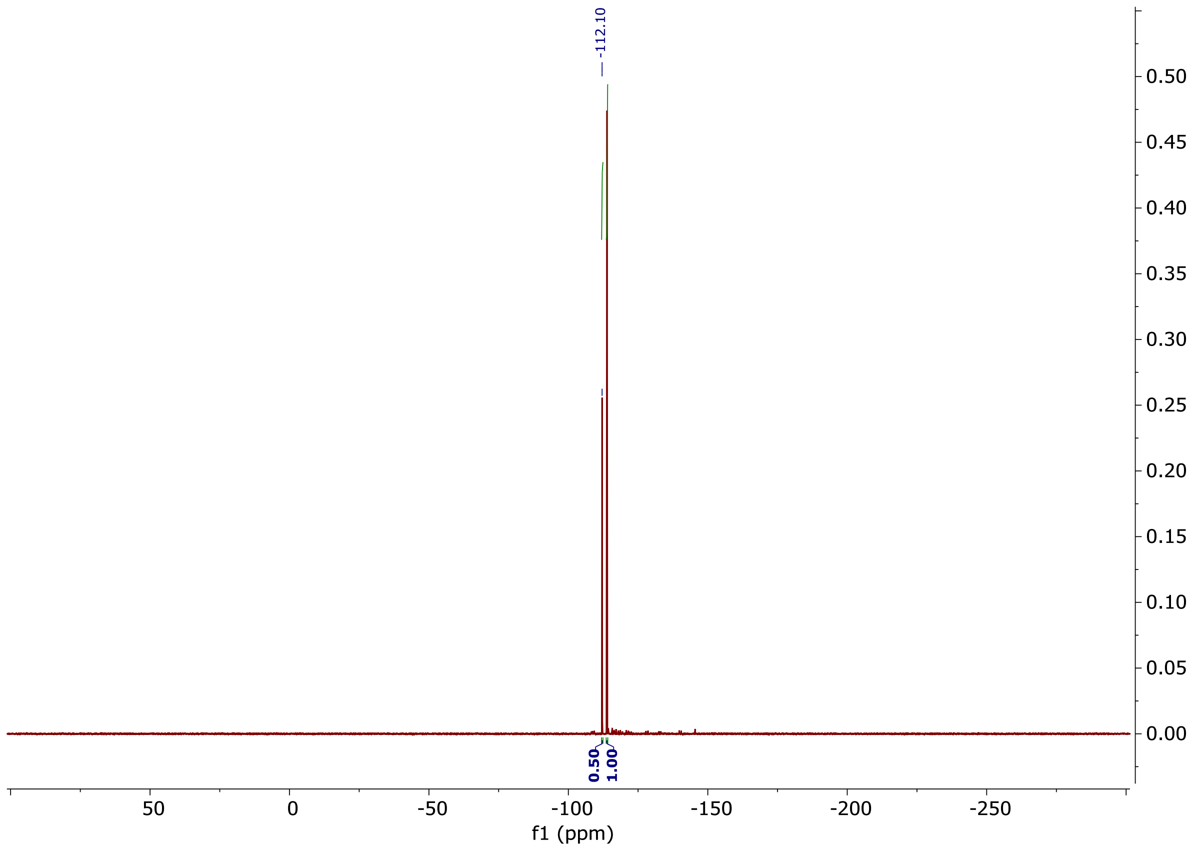

Figure S8.41. ^{19}F NMR (376 MHz) spectrum of the crude obtained after the 0.06 mmol scale divided cell electrolysis of perfluorodecalin in dry THF + 5000 ppm H_2O (10 *F*) with 50 µL of fluorobenzene as internal standard. The fluoride (F^-) peak is visible at -112.10 ppm.

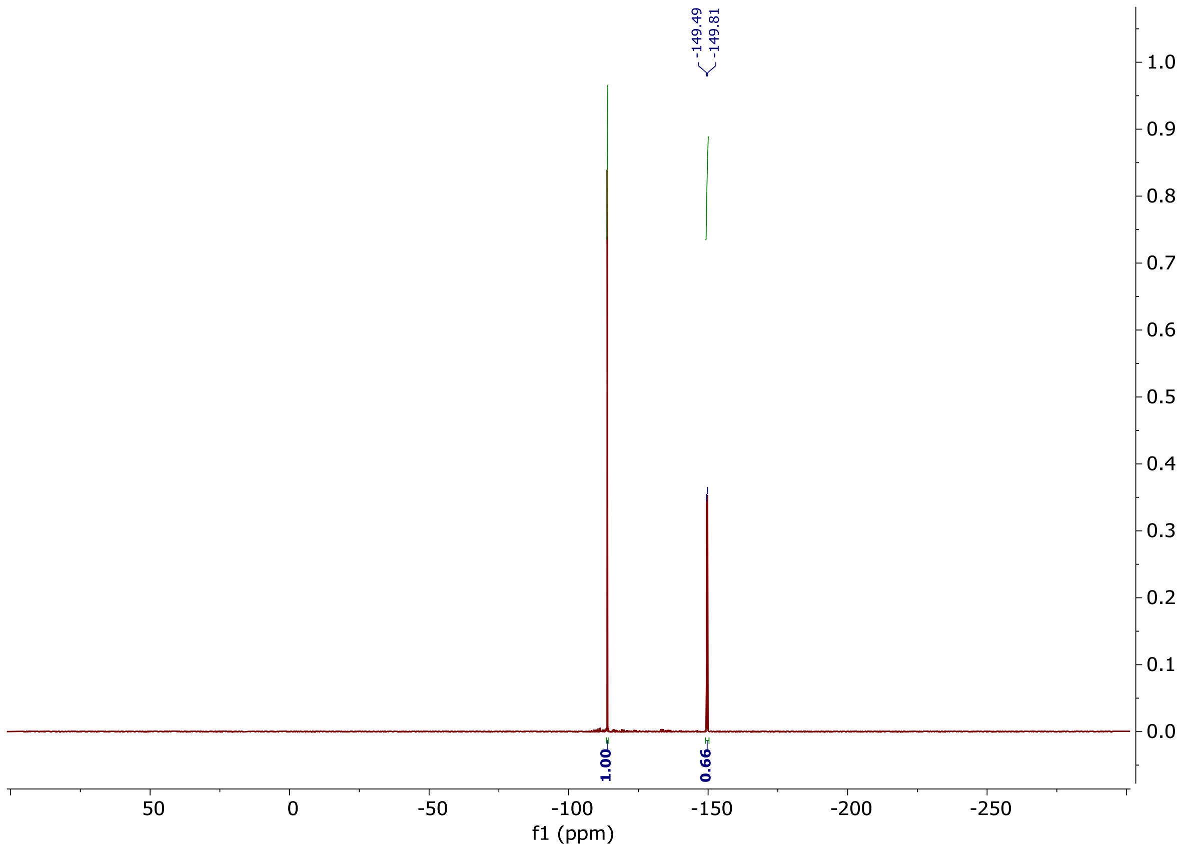

Figure S8.42. ^{19}F NMR (376 MHz) spectrum of the crude obtained after the 0.06 mmol scale divided cell electrolysis of perfluorodecalin in dry THF (10 *F*) with 0.1 M TBABr + 0.1 M $TBAClO_4$ instead of 0.2 M TBABr as supporting salt and 50 µL of fluorobenzene as internal standard. The bifluoride (HF_2^-) doublet is visible at -149.65 ppm (*J*=119.3 Hz).

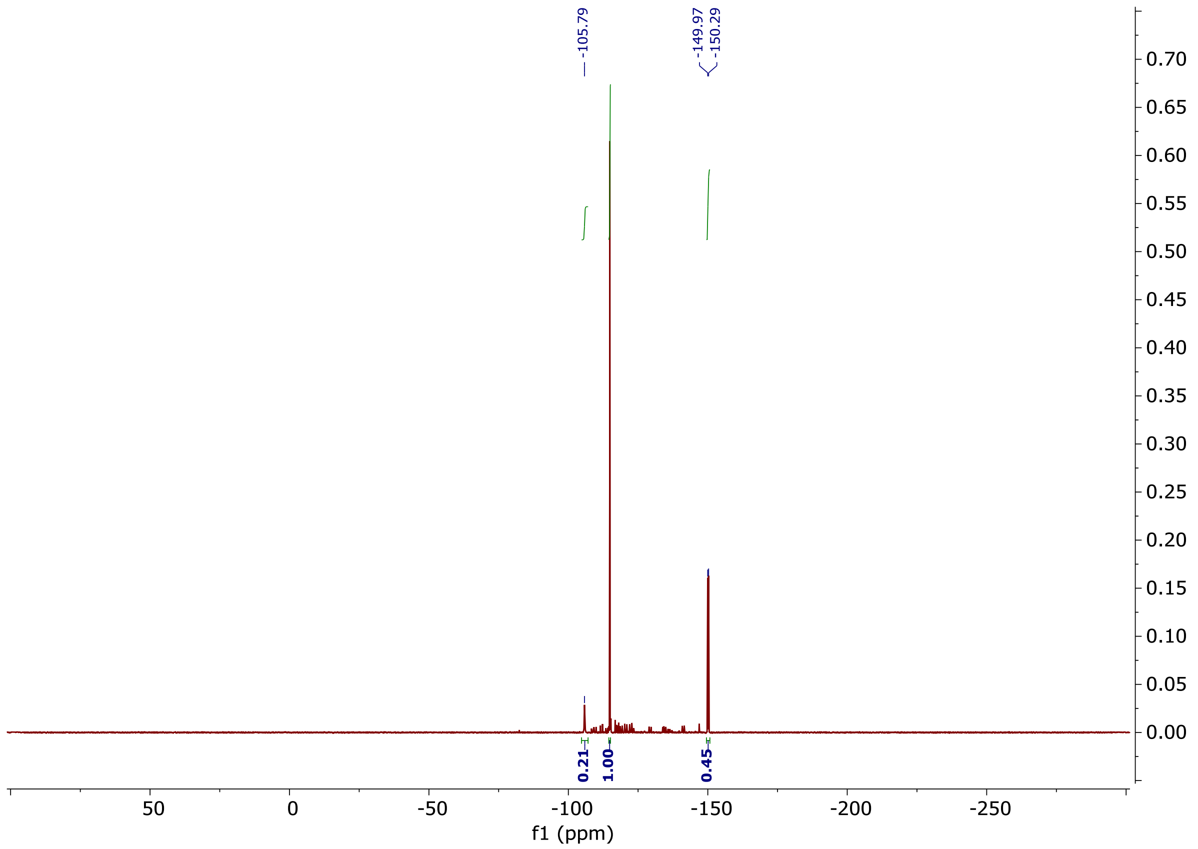

Figure S8.43. ^{19}F NMR (376 MHz) spectrum of the crude obtained after the 0.06 mmol scale divided cell electrolysis of perfluorodecalin in dry THF (10 *F*) with 0.2 M $TBAClO_4$ instead of 0.2 M TBABr as supporting salt and 50 µL of fluorobenzene as internal standard. The fluoride (F^-) peak is visible at -105.79 ppm and the bifluoride (HF_2^-) doublet is visible at -150.13 ppm (*J*=119.7 Hz).

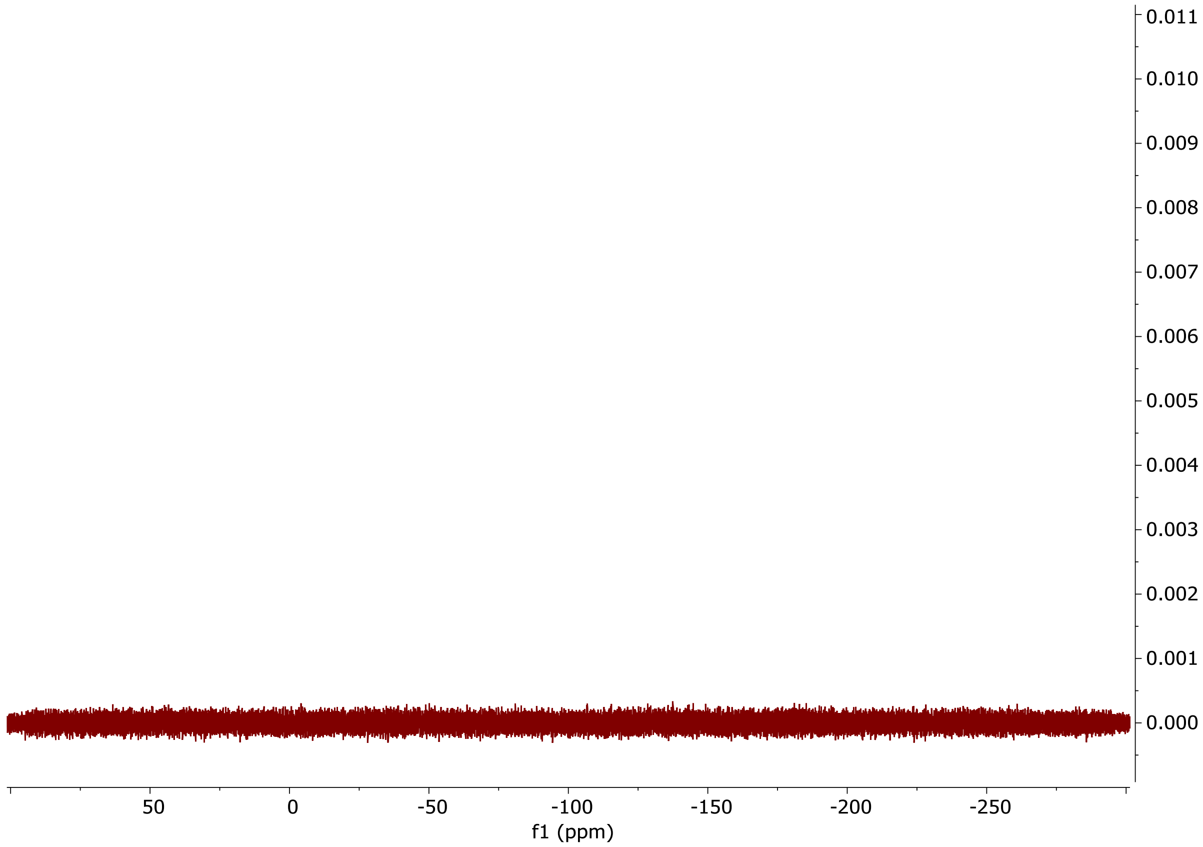

Figure S8.44. ^{19}F NMR (376 MHz) spectrum of the crude obtained after mixing 0.06 mmol of dry CsF in dry acetone for 3h.

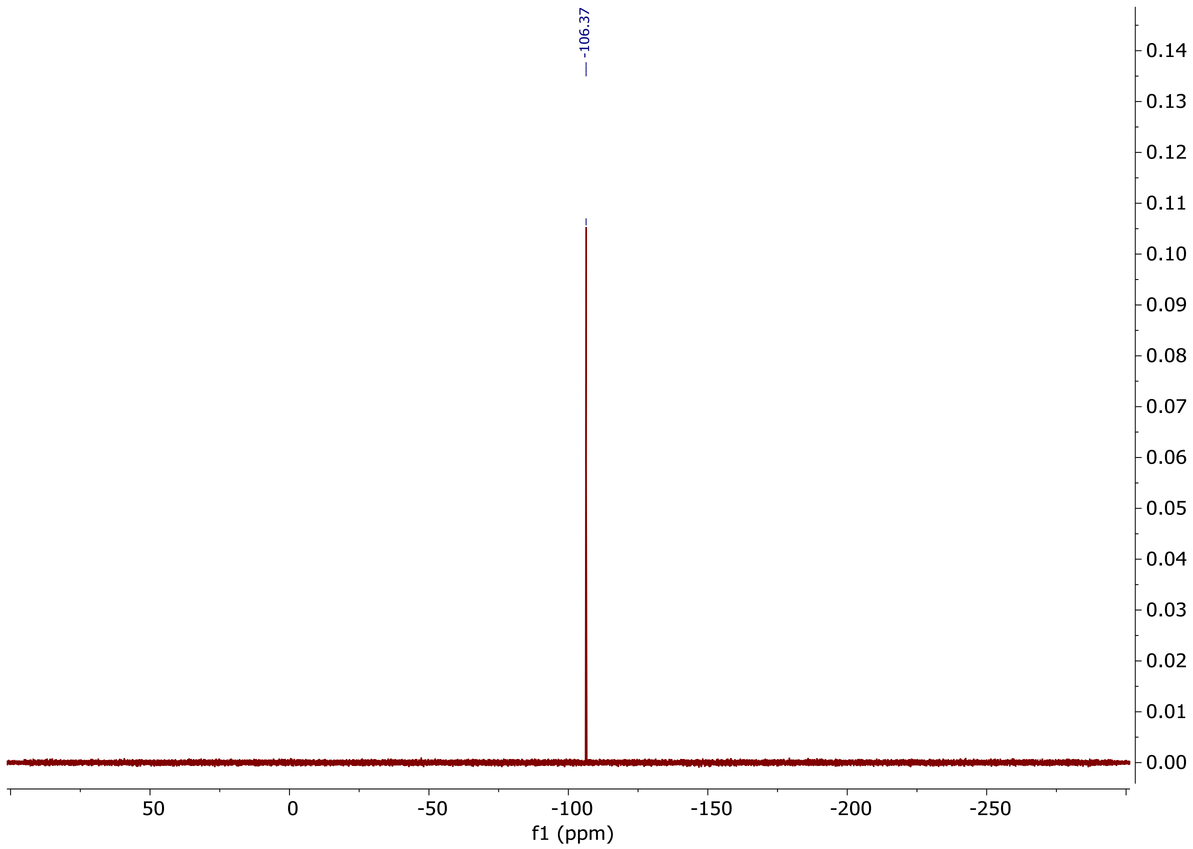

Figure S8.45. ^{19}F NMR (376 MHz) spectrum of the crude obtained after mixing 0.06 mmol of dry CsF in dry acetone/0.1 M TBABr for 3h. A fluoride peak is visible at -106.37 ppm.

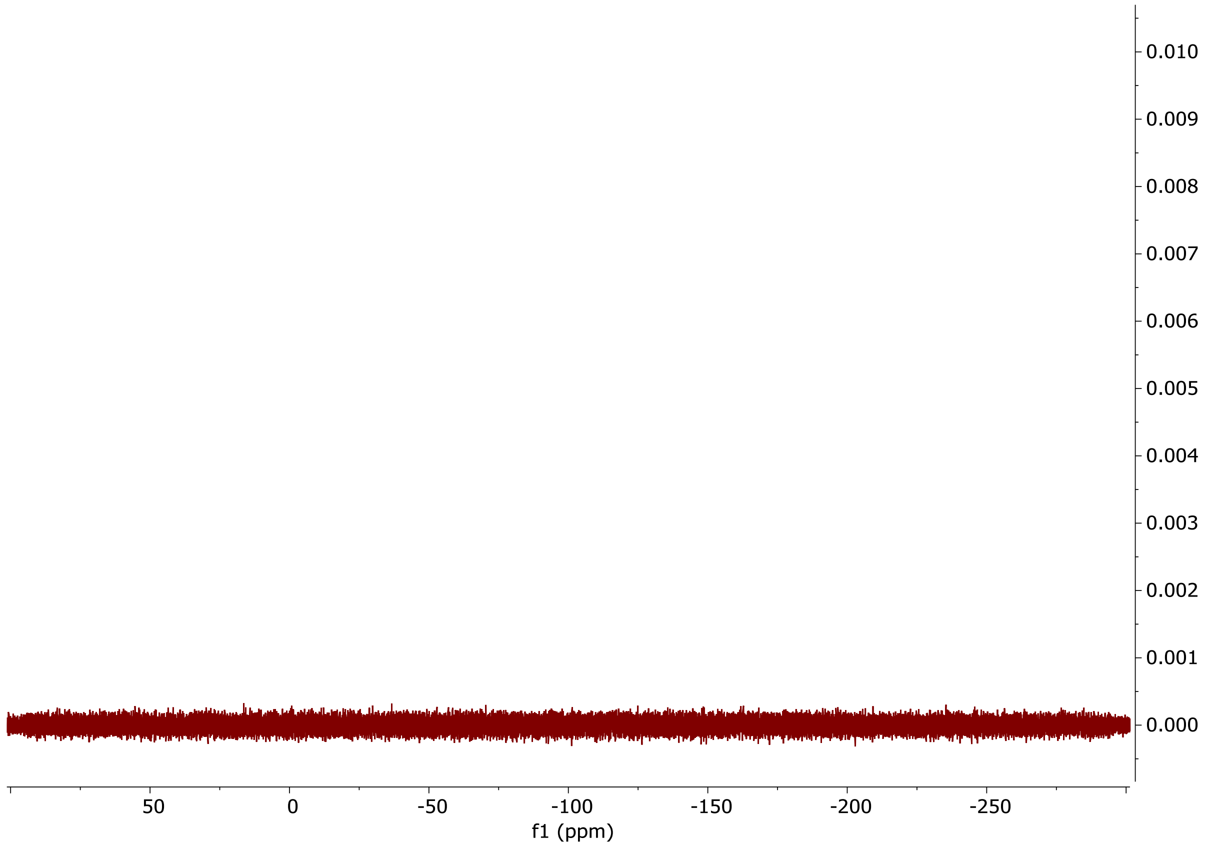

Figure S8.46. ^{19}F NMR (376 MHz) spectrum of the crude obtained after mixing 0.06 mmol of dry CsF in dry THF for 3h.

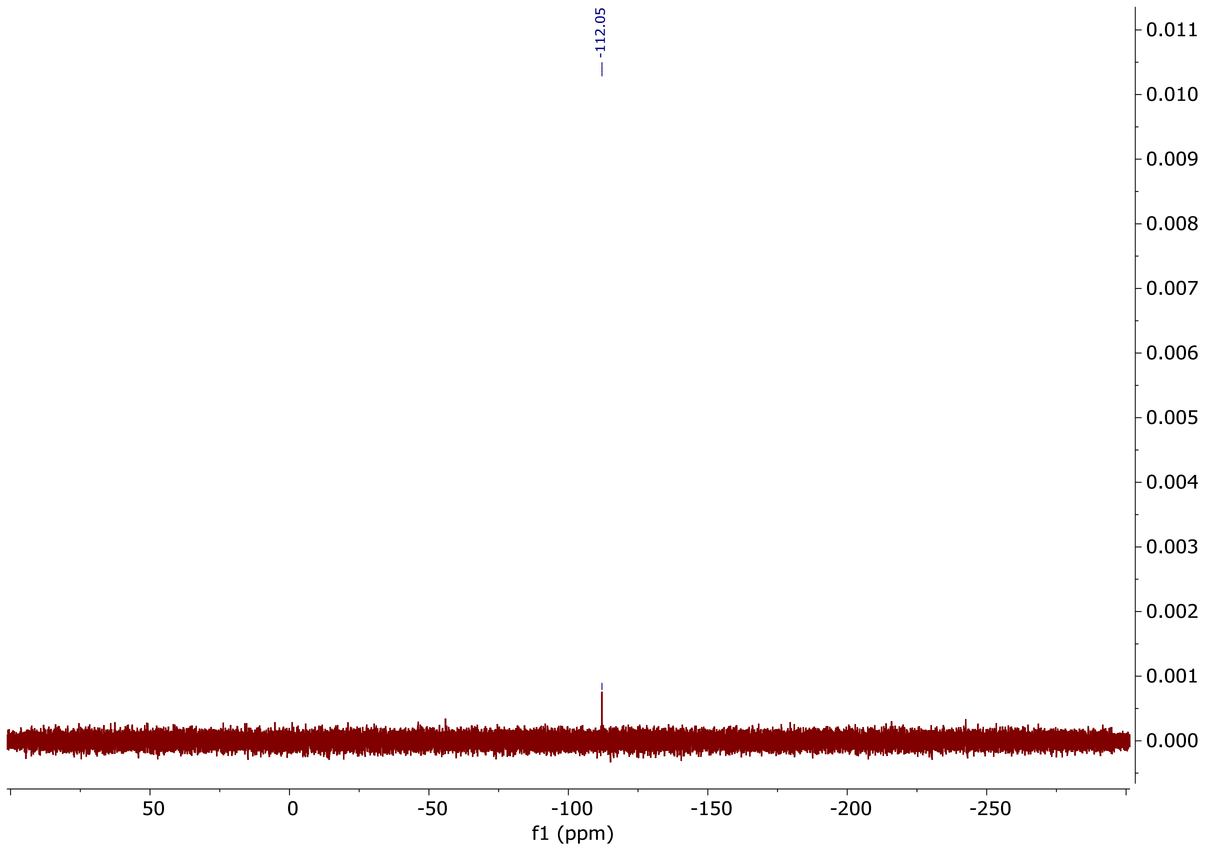

Figure S8.47. ^{19}F NMR (376 MHz) spectrum of the crude obtained after mixing 0.06 mmol of dry TMAF in dry THF/0.1 M TBABr for 3h. A very small fluoride peak and bifluoride doublet is visible at -112.05.

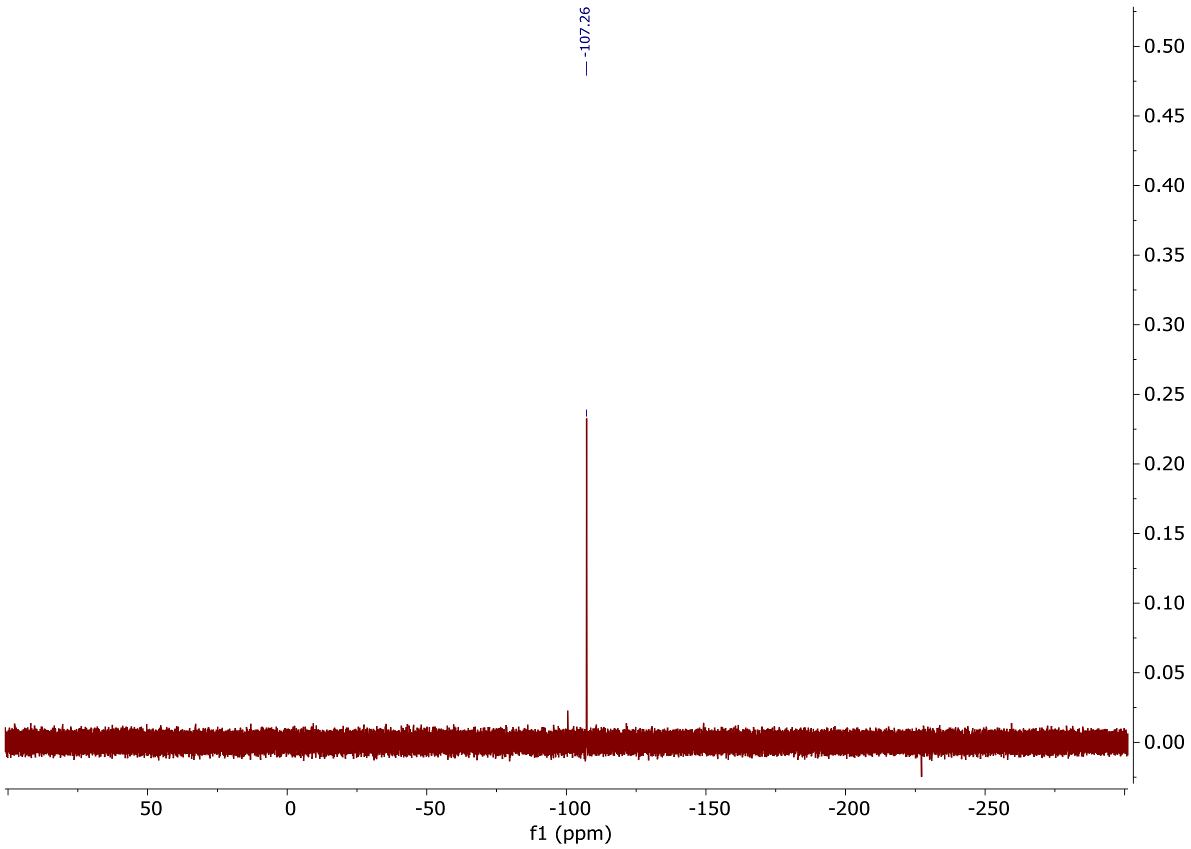

Figure S8.48. ^{19}F NMR (376 MHz) spectrum of the crude obtained after mixing 0.06 mmol of dry TMAF in dry acetone for 3h. A fluoride peak is visible at -107.26 ppm.

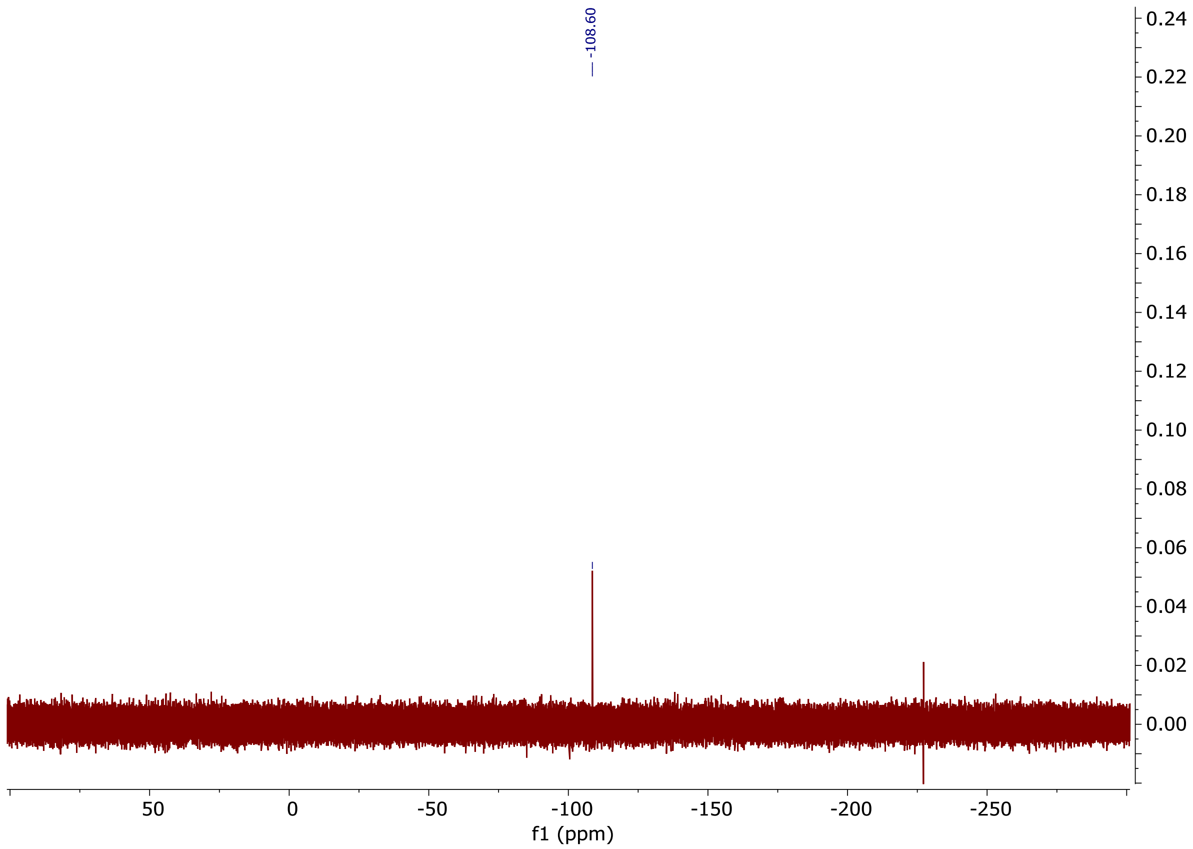

Figure S8.49. ^{19}F NMR (376 MHz) spectrum of the crude obtained after mixing 0.06 mmol of dry TMAF in dry acetone/0.1 M TBABr for 3h. A fluoride peak is visible at -108.60 ppm.

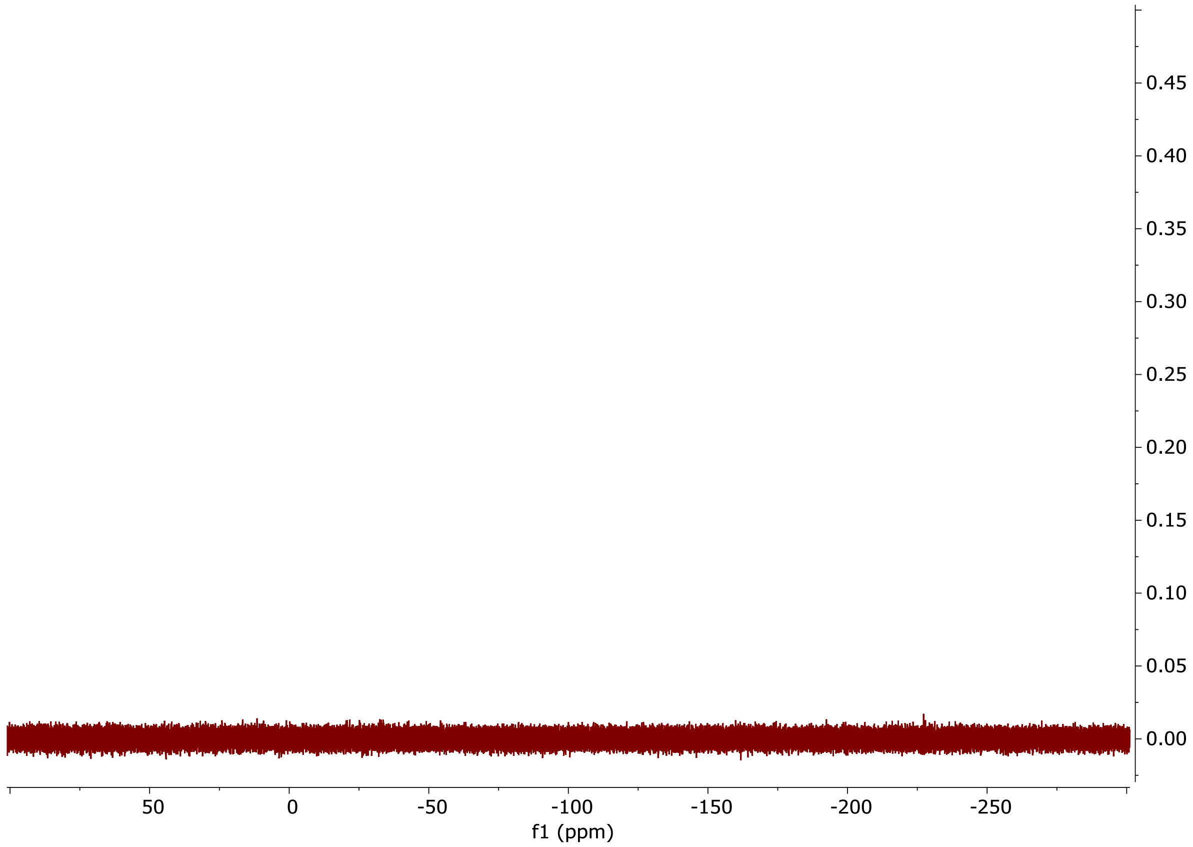

Figure S8.50. ^{19}F NMR (376 MHz) spectrum of the crude obtained after mixing 0.06 mmol of dry TMAF in dry THF for 3h.

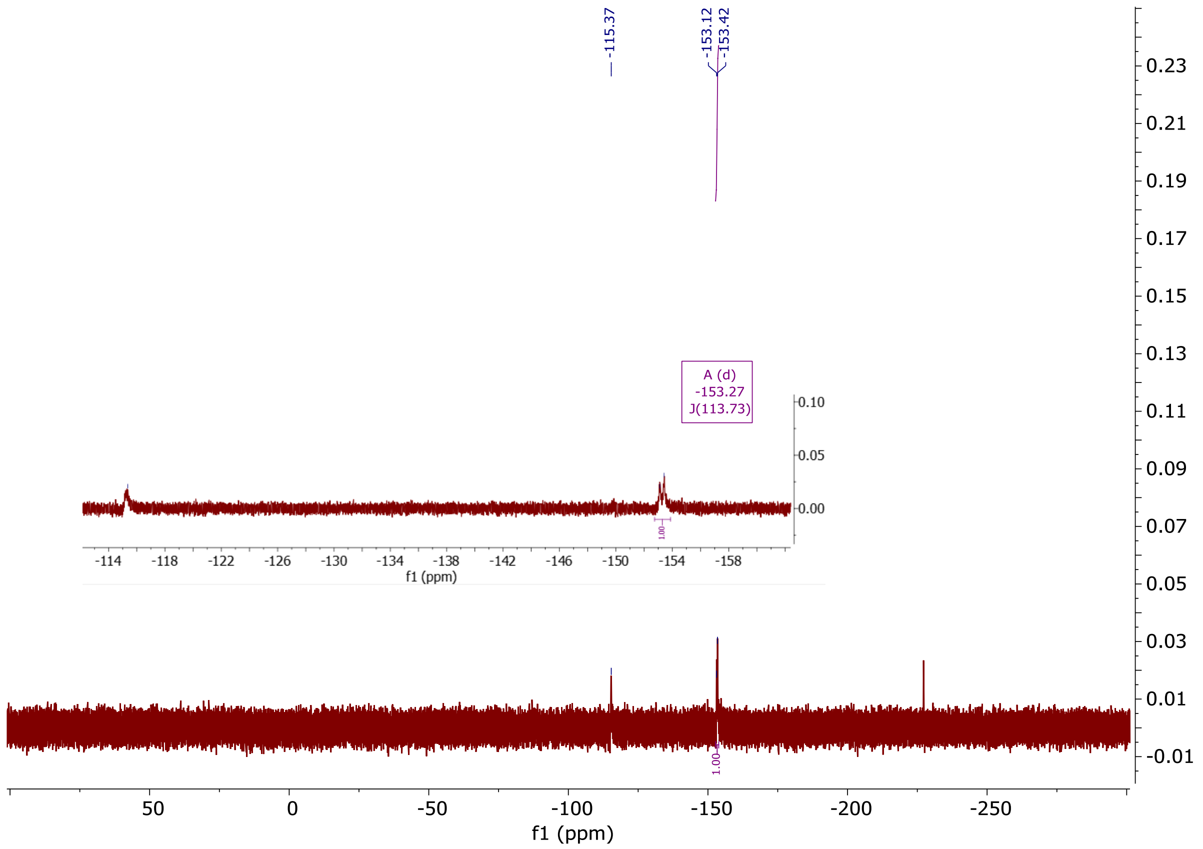

Figure S8.51. ^{19}F NMR (376 MHz) spectrum of the crude obtained after mixing 0.06 mmol of dry TMAF in dry THF/0.1 M TBABr for 3h. Very small fluoride peak and bifluoride doublet are visible at -115.37 and -153.27 ppm, respectively.

c. Divided cell electrolysis of PFAS – Isolation of fluoride salts

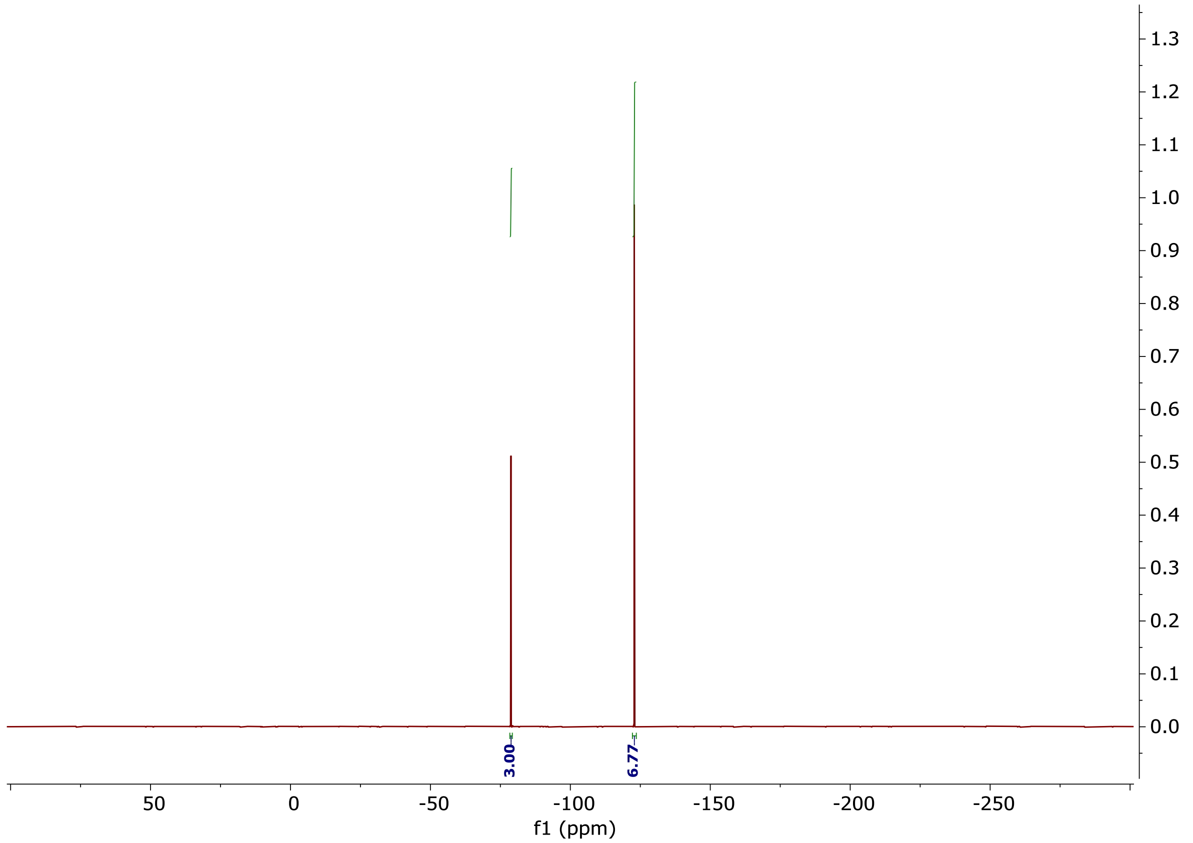

Figure S8.52. Quantitative ^{19}F NMR (376 MHz, D_2O) of 1.08 mg of LiF^{PFAS} from the divided cell electrolysis of PFD in acetone and 0.84 mg of sodium triflate as internal standard. LiF is 80% pure.

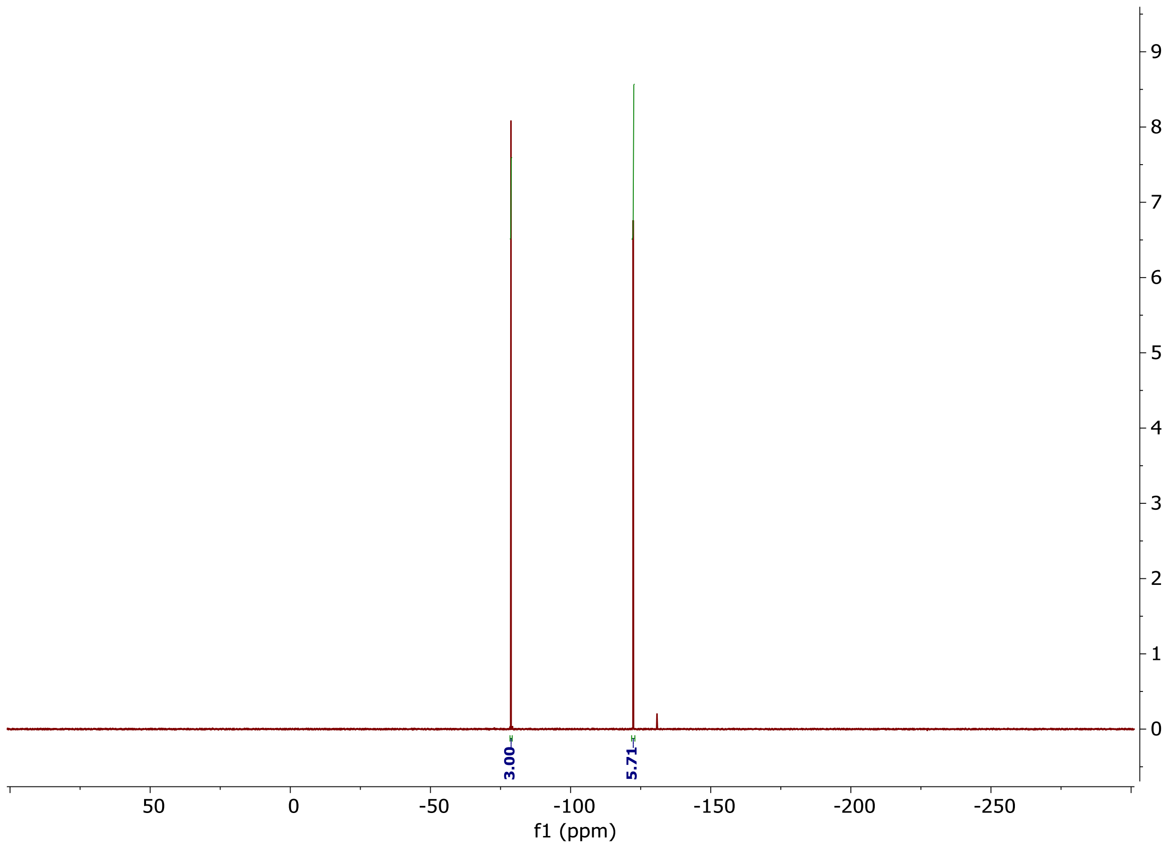

Figure S8.53. Quantitative ^{19}F NMR (376 MHz, D_2O) of 3.04 mg of NaF^{PFAS} from the divided cell electrolysis of PFD in acetone and 2.02 mg of sodium triflate as internal standard. NaF is 93% pure.

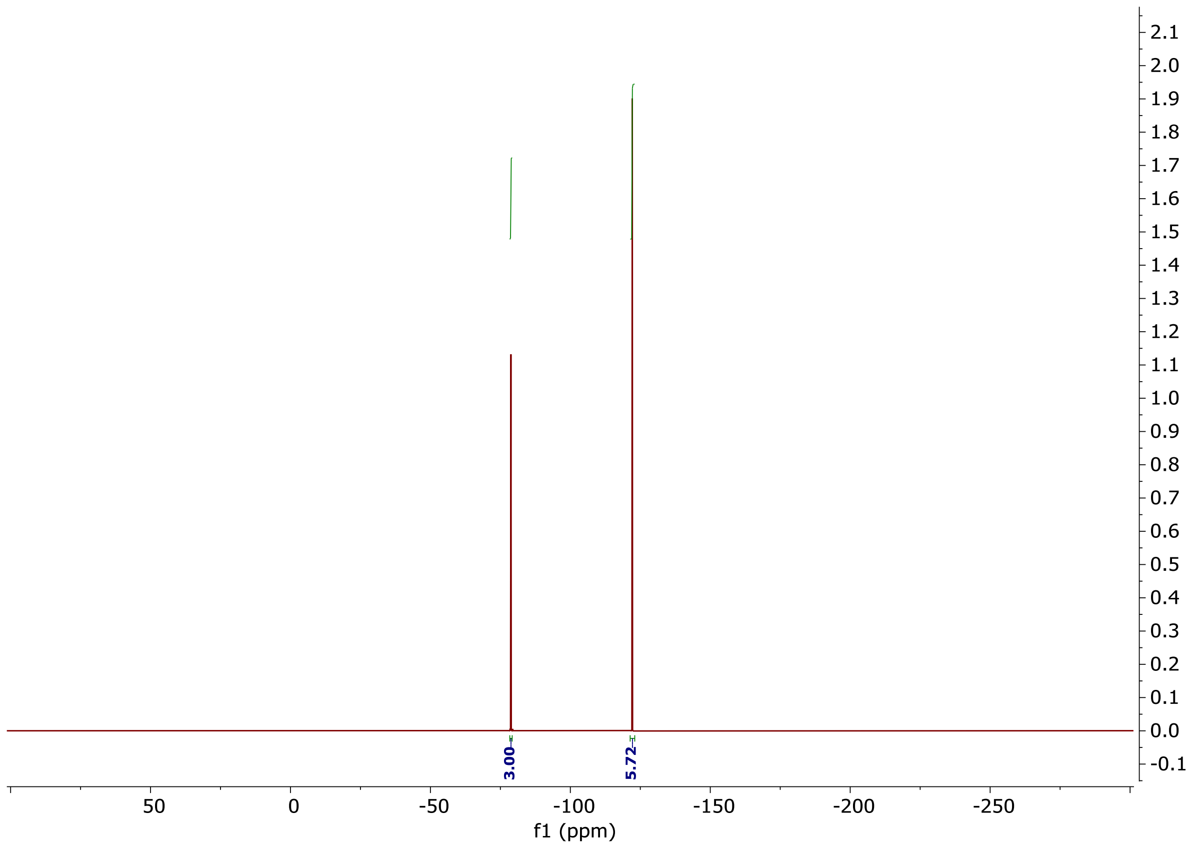

Figure S8.54. Quantitative ^{19}F NMR (376 MHz, D_2O) of 4.39 mg of KF^{PFAS} from the divided cell electrolysis of PFD in acetone and 2.03 mg of sodium triflate as internal standard. KF is 89% pure.

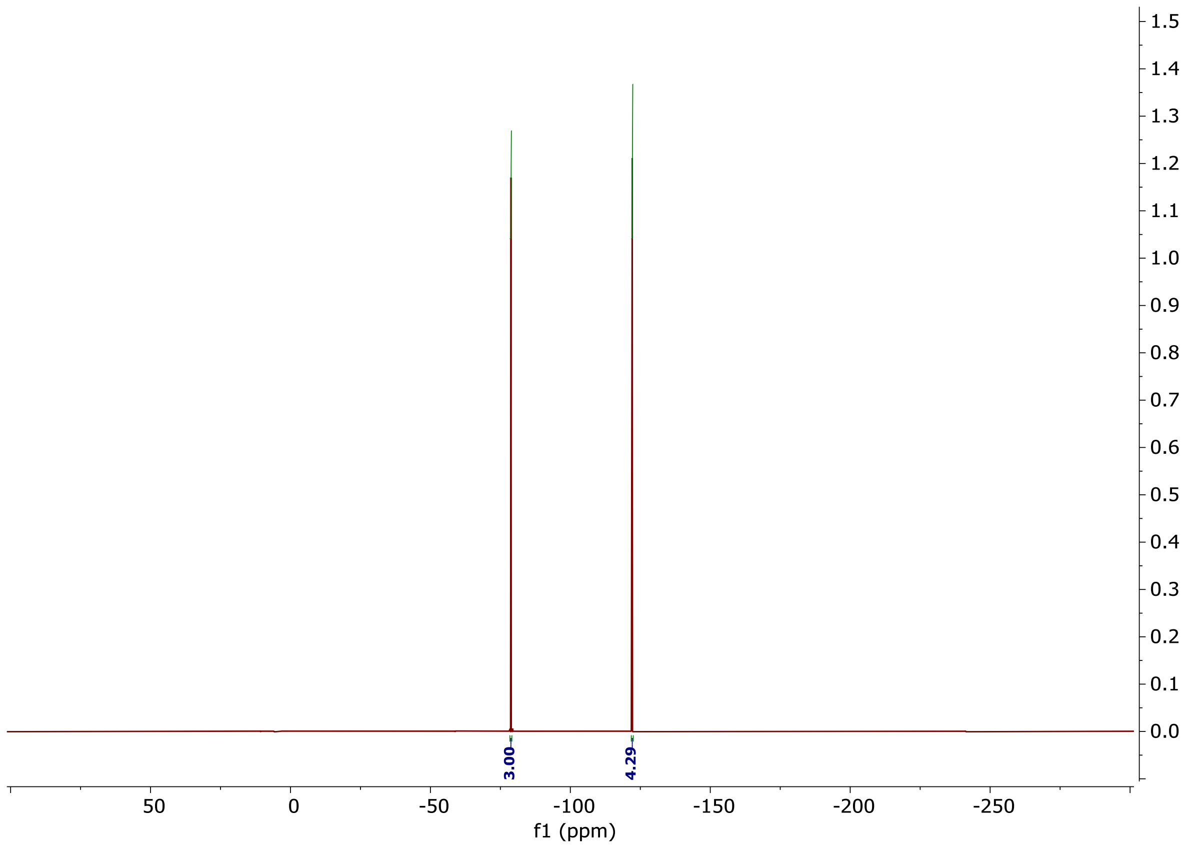

Figure S8.55. Quantitative ^{19}F NMR (376 MHz, D_2O) of 2.88 mg of KF^{PFAS} from the divided cell electrolysis of 1H,1H,2H-perfluoro-1-decene in MeCN and 1.82 mg of sodium triflate as internal standard. KF is 92% pure.

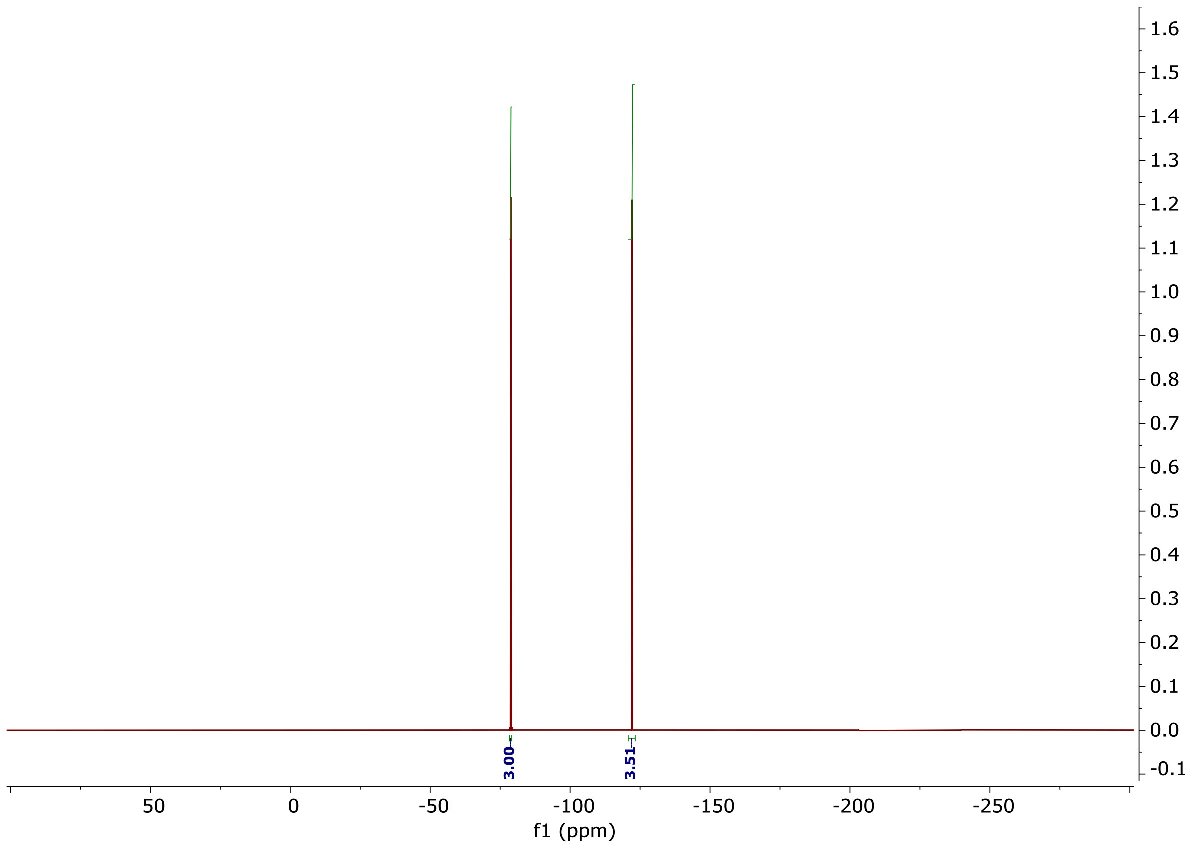

Figure S8.56. Quantitative ^{19}F NMR (376 MHz, D_2O) of 3.19 mg of KF^{PFAS} from the divided cell electrolysis of PFOS-TBA in MeCN and 2.44 mg of sodium triflate as internal standard. KF is 91% pure.

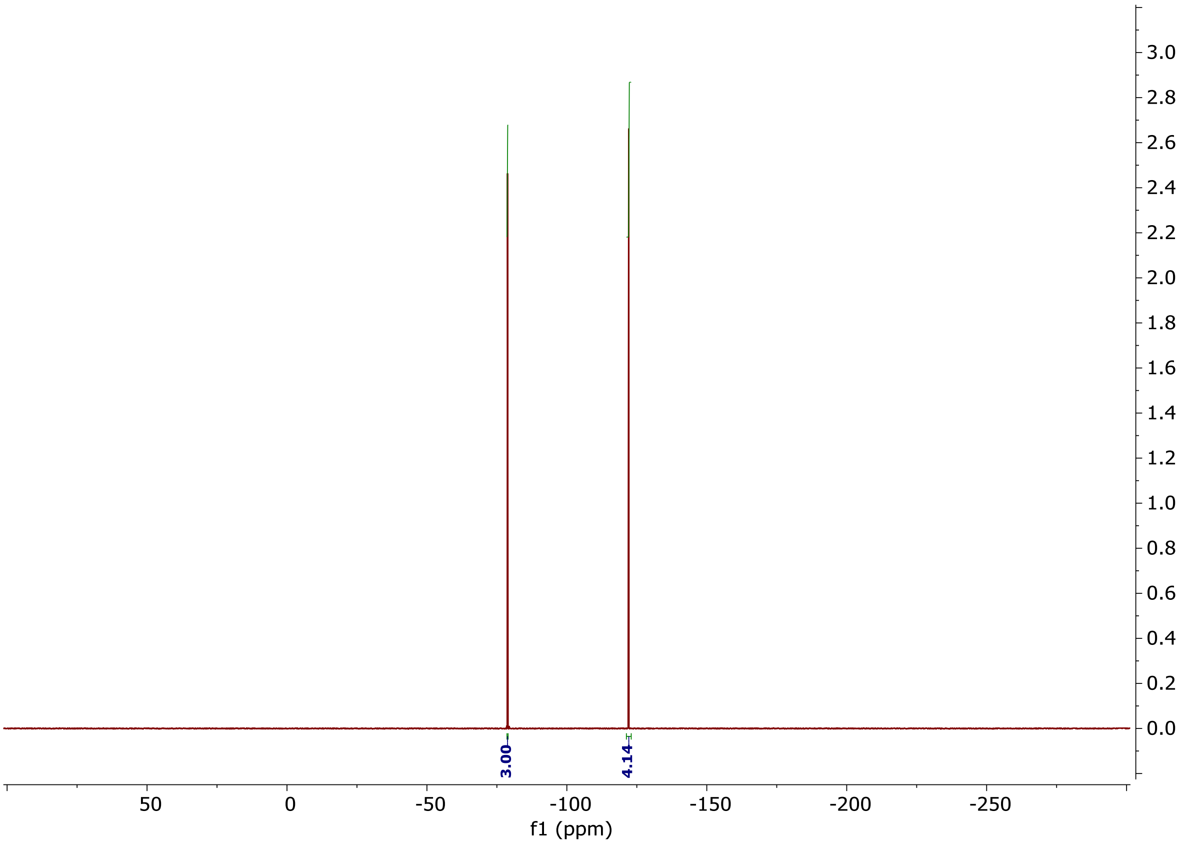

Figure S8.57. Quantitative ^{19}F NMR (376 MHz, D_2O) of 2.49 mg of KF^{PFAS} from the divided cell electrolysis of PFOA in MeCN and 1.40 mg of sodium triflate as internal standard. KF is 79% pure.

d. Divided cell electrolysis – TBAT synthesis

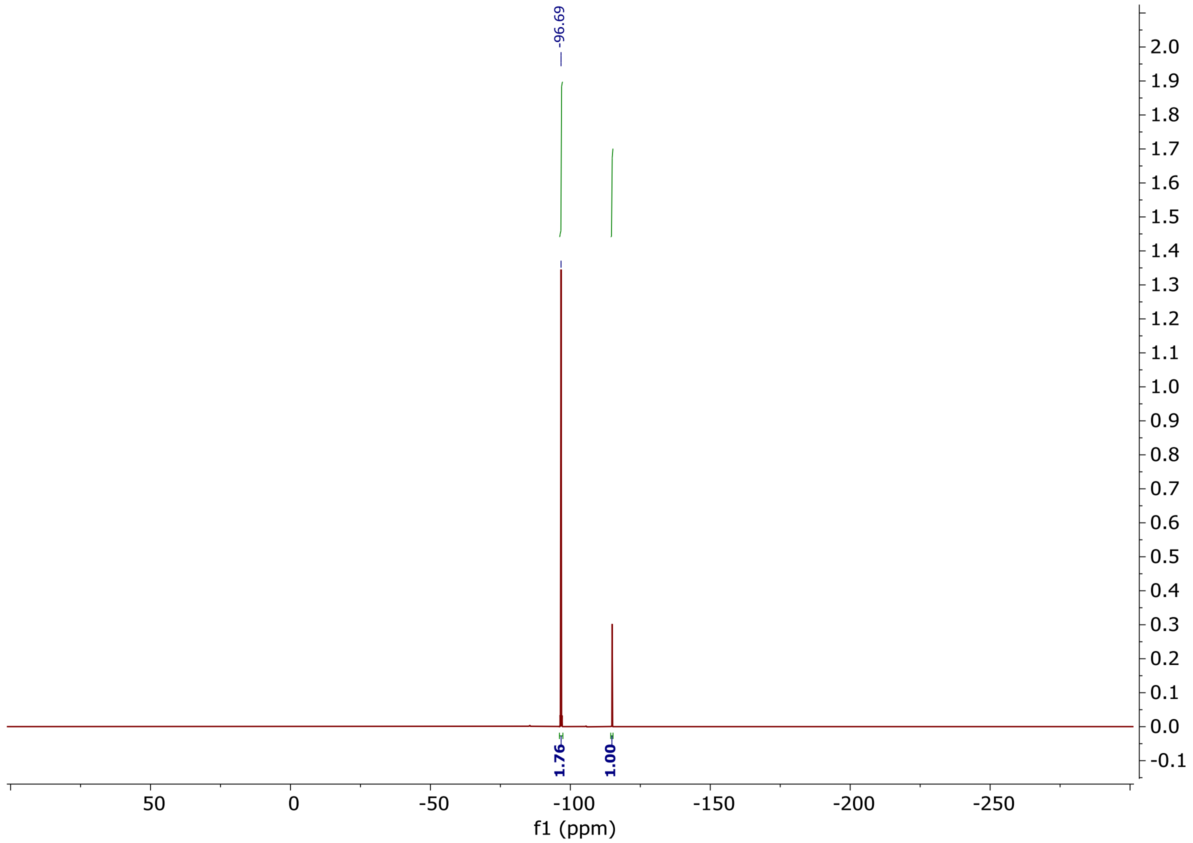

Figure S8.58. ^{19}F NMR (376 MHz) spectrum of the crude obtained after the 0.1 mmol divided cell electrolysis for TBAT synthesis from perfluorodecalin (24 µL) in dry acetonitrile with 50 µL of fluorobenzene as internal standard. The TBAT peak is visible at -96.69 ppm.

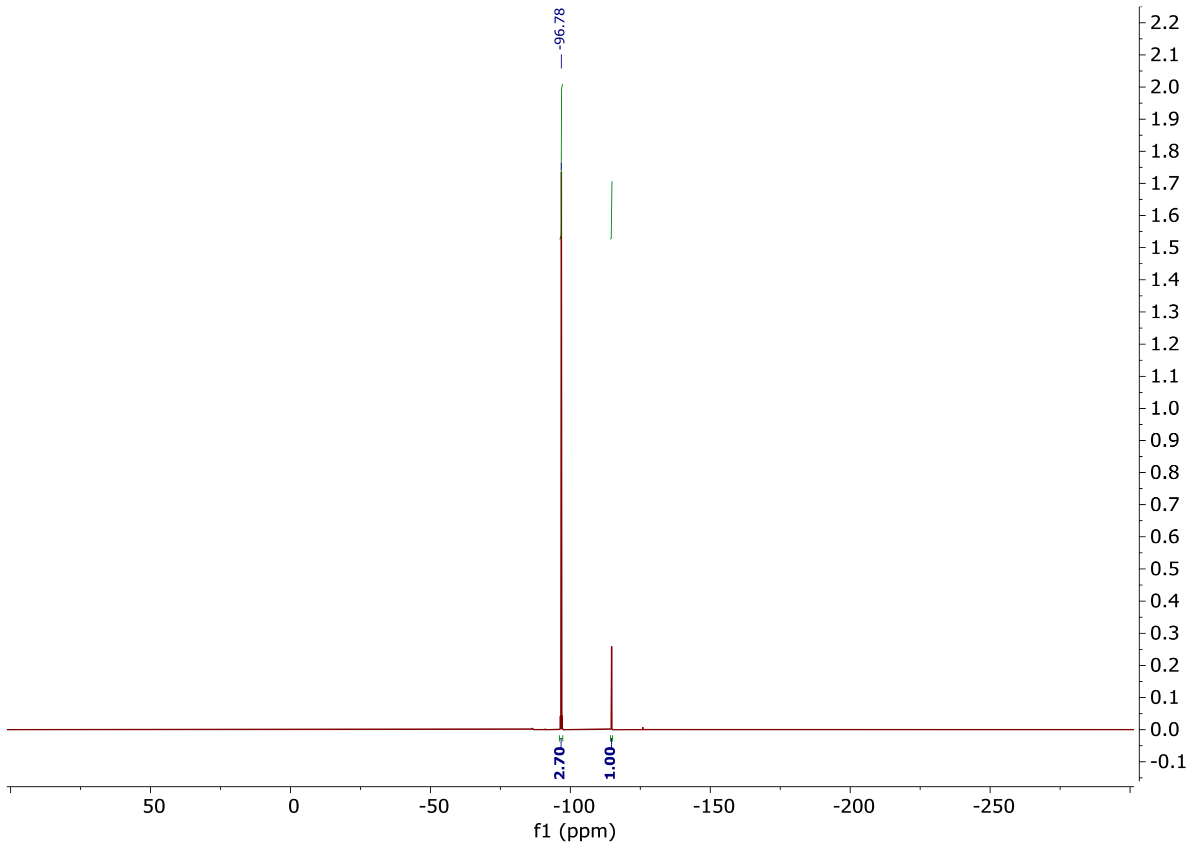

Figure S8.59. ^{19}F NMR (376 MHz) spectrum of the crude obtained after the 1.0 mmol divided cell electrolysis for TBAT synthesis from perfluorodecalin (242 µL) in dry acetonitrile with 400 µL of fluorobenzene as internal standard. The TBAT peak is visible at -96.78 ppm.

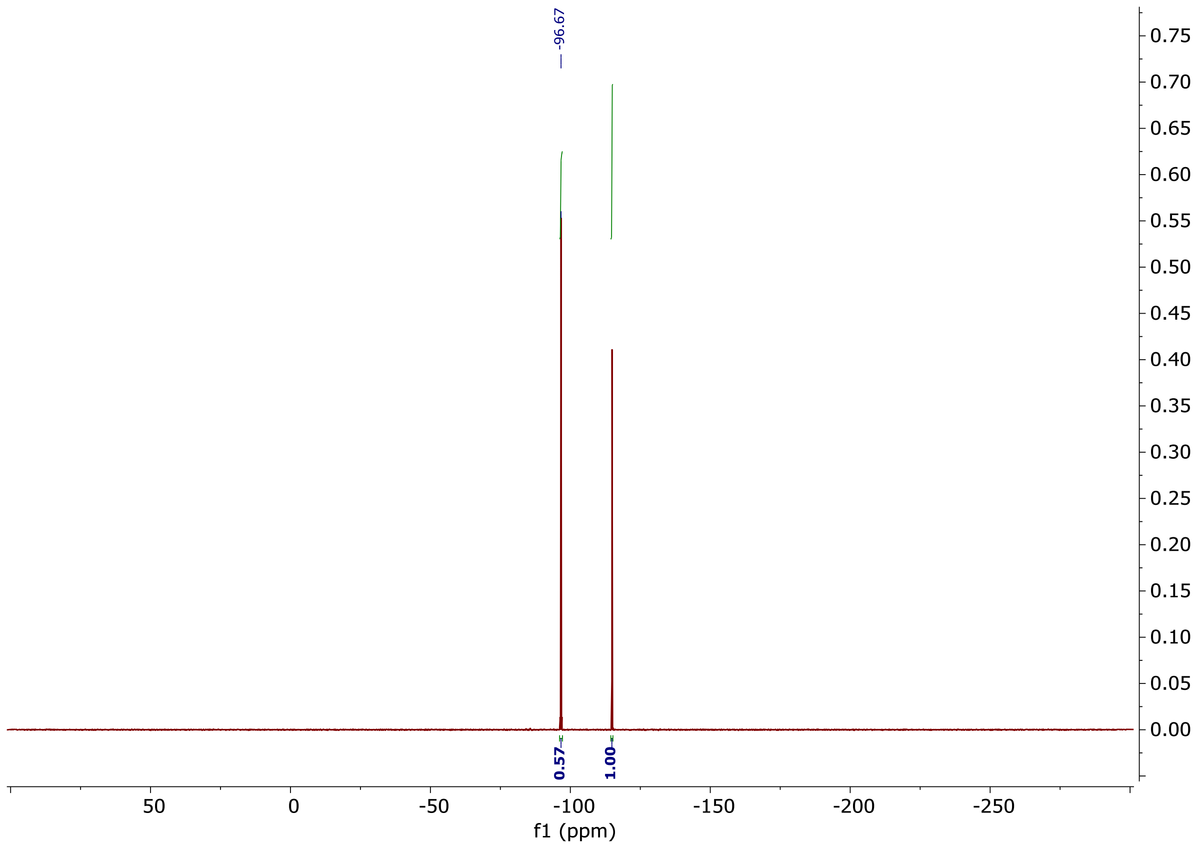

Figure S8.60. ^{19}F NMR (376 MHz) spectrum of the crude obtained after the 0.1 mmol divided cell electrolysis for TBAT synthesis from perfluorooctane (25 µL) in dry acetonitrile with 50 µL of fluorobenzene as internal standard. The TBAT peak is visible at -96.67 ppm.

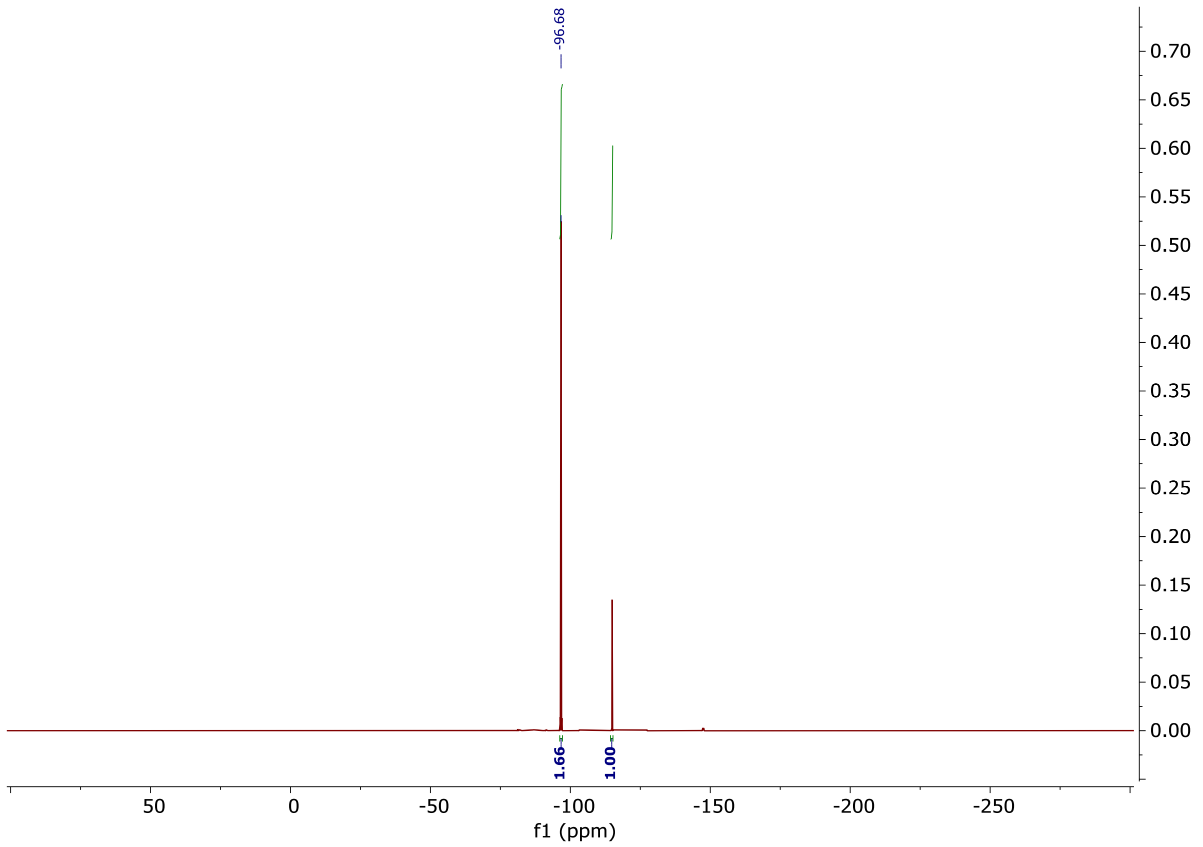

Figure S8.61. ^{19}F NMR (376 MHz) spectrum of the crude obtained after the 0.1 mmol divided cell electrolysis for TBAT synthesis from 1H,1H,2H-perfluoro-1-decene (27 µL)in dry acetonitrile with 50 µL of fluorobenzene as internal standard. The TBAT peak is visible at -96.68 ppm.

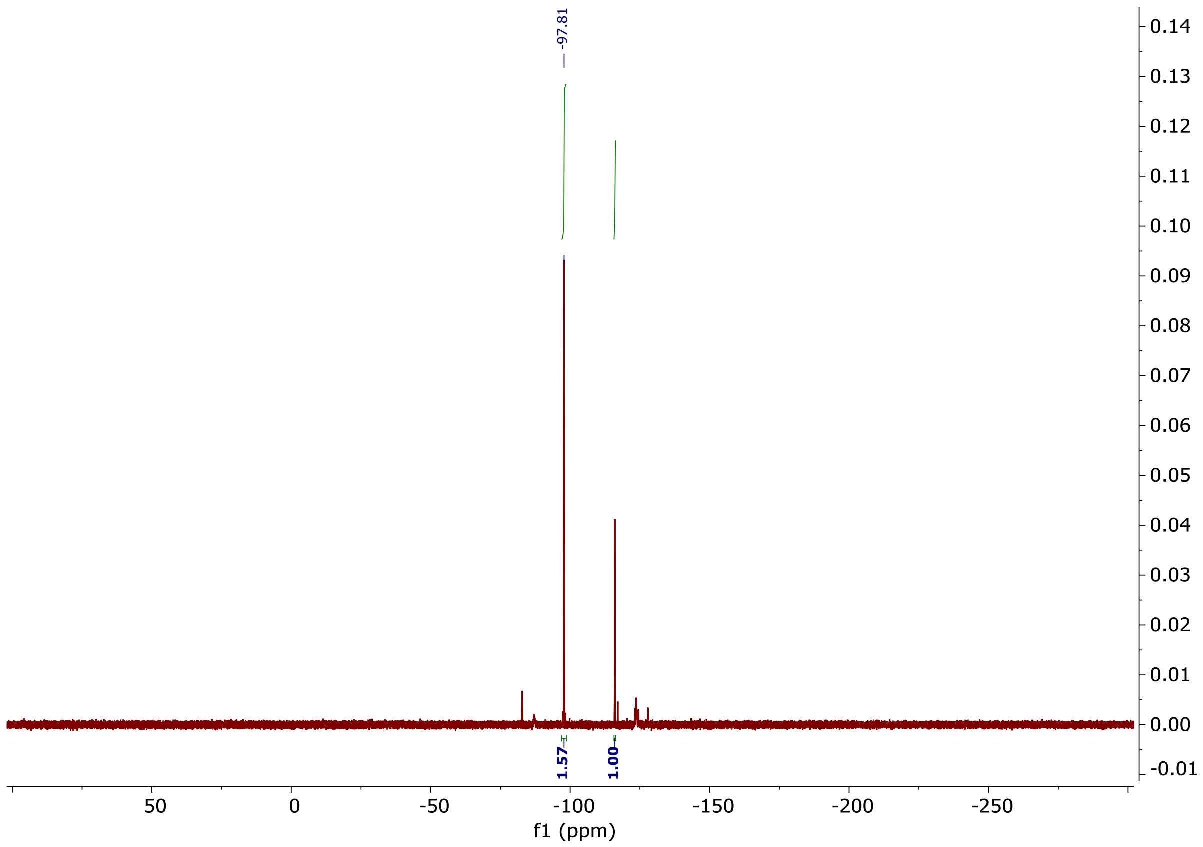

Figure S8.62. ^{19}F NMR (283 MHz) spectrum of the crude obtained after the 0.1 mmol divided cell electrolysis for TBAT synthesis from PFNA (47 mg) in dry acetonitrile with 50 µL of fluorobenzene as internal standard. The TBAT peak is visible at -97.81 ppm.

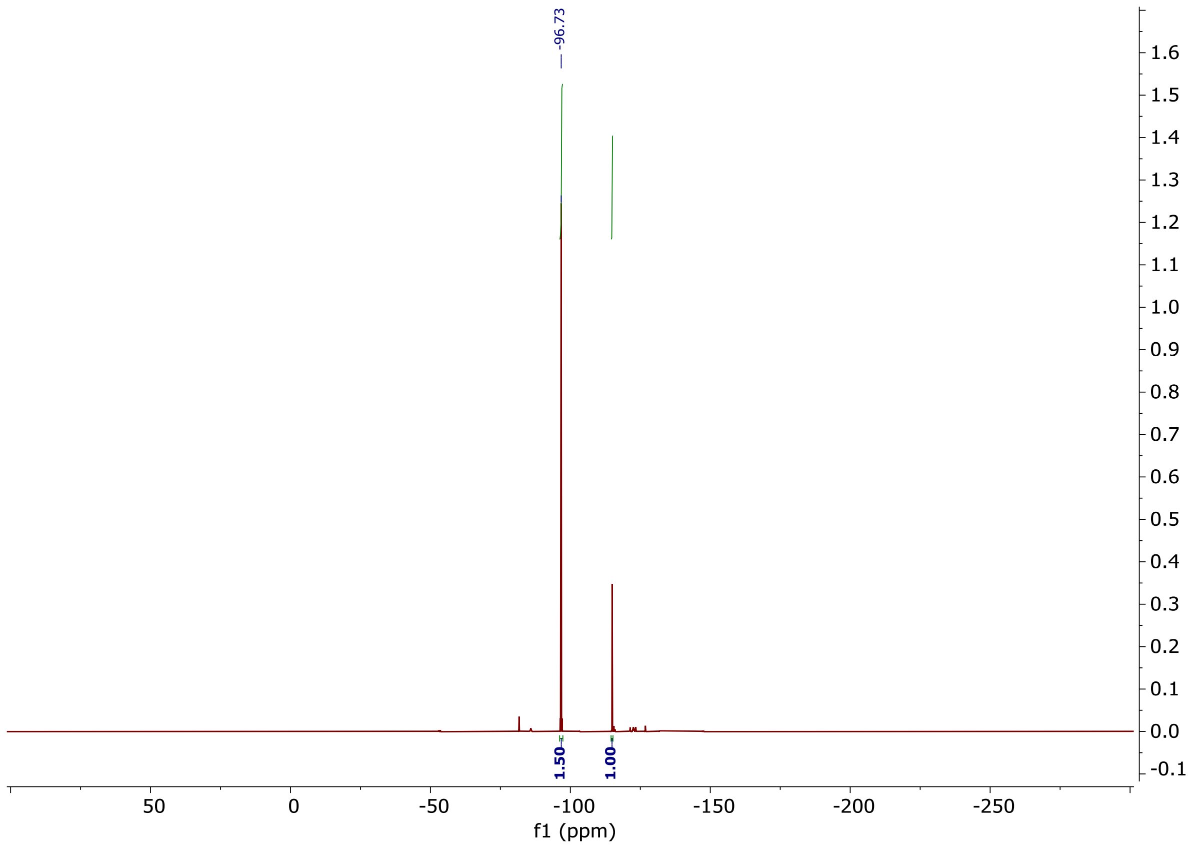

Figure S8.63. ^{19}F NMR (376 MHz) spectrum of the crude obtained after the 0.1 mmol divided cell electrolysis for TBAT synthesis from PFOS-TBA (74 mg) in dry acetonitrile with 50 µL of fluorobenzene as internal standard. The TBAT peak is visible at -96.73 ppm.

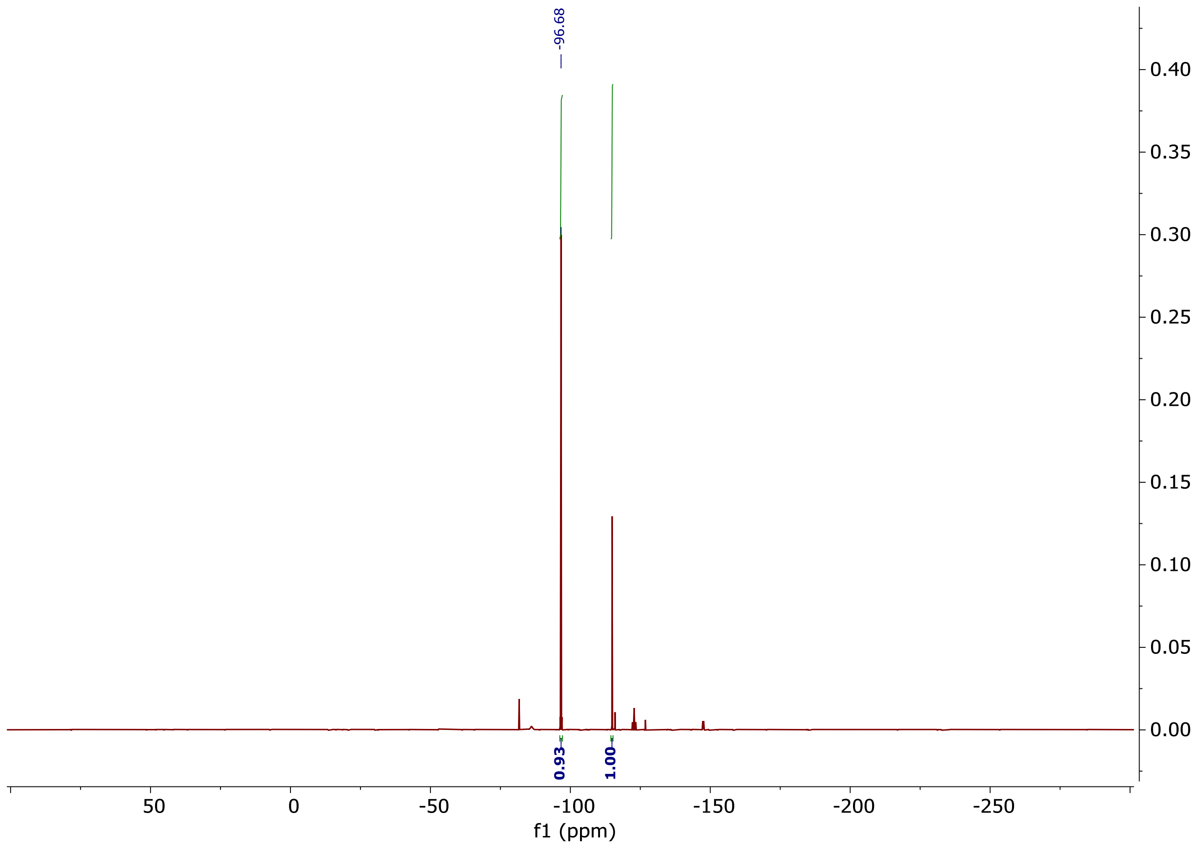

Figure S8.64. ^{19}F NMR (376 MHz) spectrum of the crude obtained after the divided cell electrolysis for TBAT synthesis from PFOA (42 mg) in dry acetonitrile with 50 µL of fluorobenzene as internal standard. The TBAT peak is visible at -96.68 ppm.

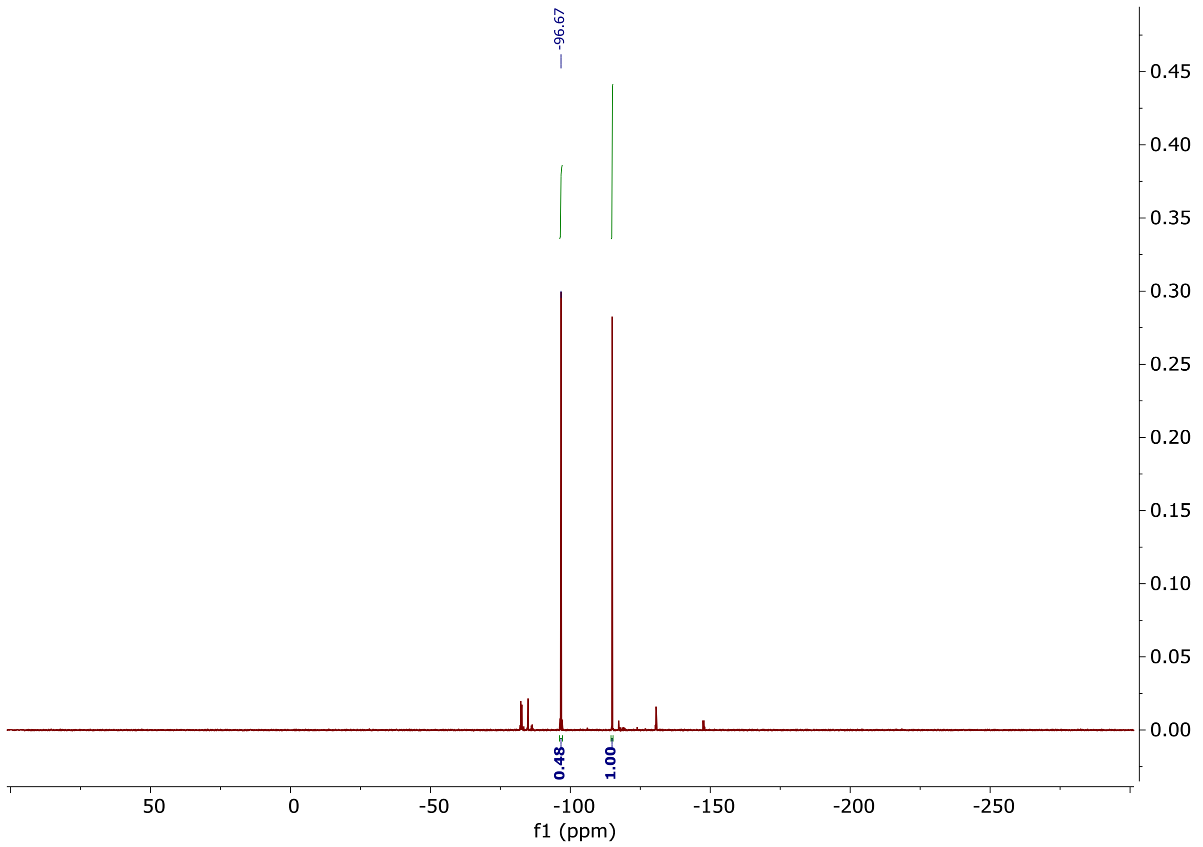

Figure S8.65. ^{19}F NMR (376 MHz) spectrum of the crude obtained after the 0.1 mmol divided cell electrolysis for TBAT synthesis from HFPO-DA (19 µL) in dry acetonitrile with 50 µL of fluorobenzene as internal standard. The TBAT peak is visible at -96.67 ppm.

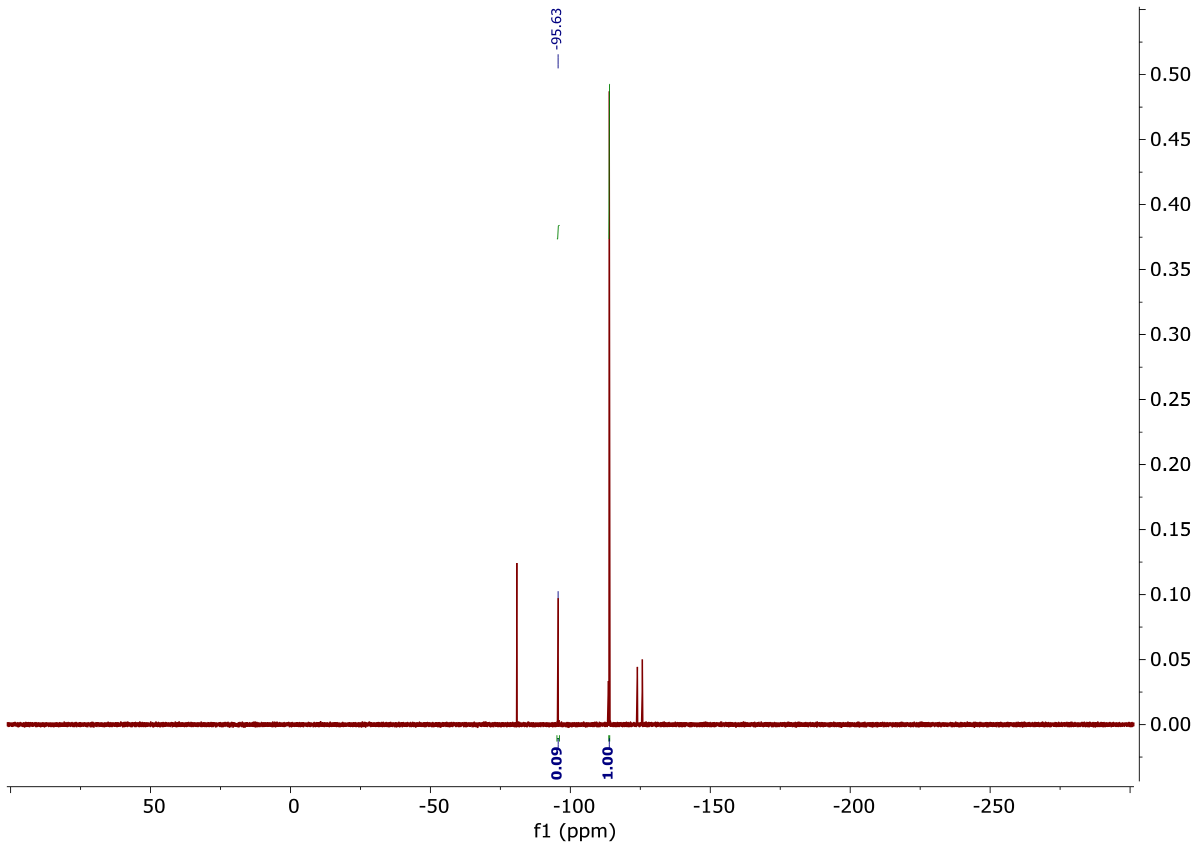

Figure S8.66. ^{19}F NMR (376 MHz) spectrum of the crude obtained after the 0.1 mmol divided cell electrolysis for TBAT synthesis from 4:2 FTSA (33 mg) in dry acetonitrile with 50 µL of fluorobenzene as internal standard. The TBAT peak is visible at -95.63 ppm.

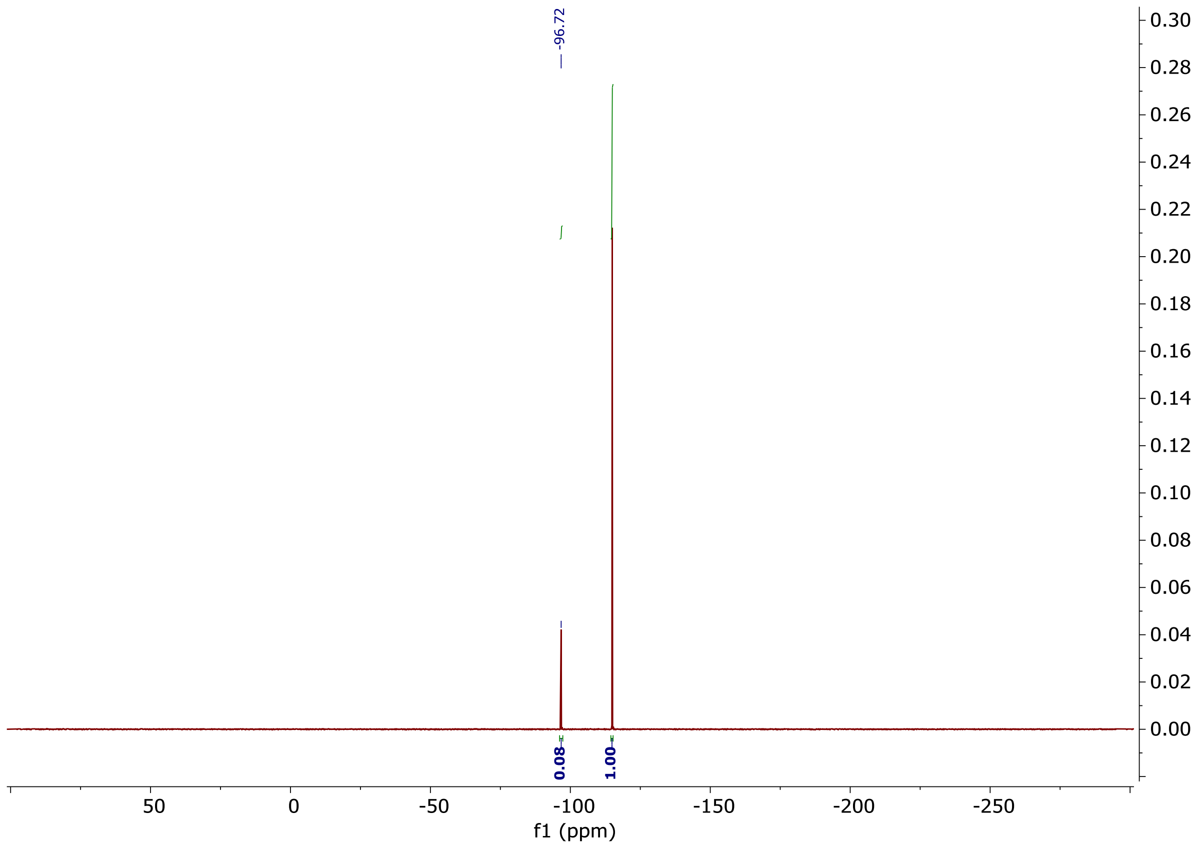

Figure S8.67. ^{19}F NMR (376 MHz) spectrum of the crude obtained after the 0.1 mmol divided cell electrolysis for TBAT synthesis from 1H,1H,2H-perfluoro-1-hexene (17 µL) in dry acetonitrile with 50 µL of fluorobenzene as internal standard. The TBAT peak is visible at -96.72 ppm.

Quantitative NMR – TBAT purity before recrystallization in trifluorotoluene

Given that $CDCl_3$ is not a practical solvent to acquire NMR spectra of TBAT ($CDCl_3$ must be freshly distilled before each NMR because TBAT is degraded by traces of acid in $CDCl_3$),[5] dry CD_3CN (dried over molecular sieves) was used as deuterated solvent.

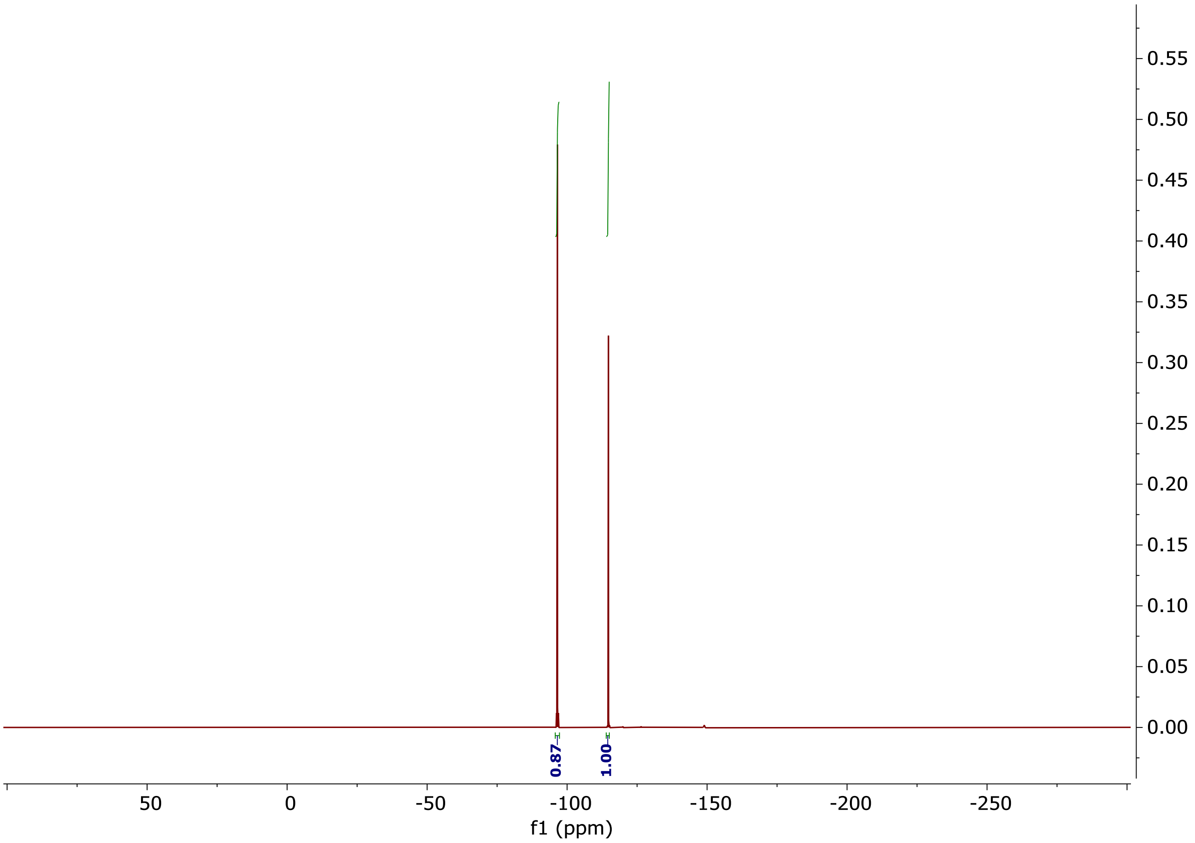

Figure S8.68. Quantitative ^{19}F NMR (376 MHz, dry CD_3CN) of 25.25 mg of TBAT^{PFAS} from perfluorodecalin and 10.0 µL of fluorobenzene as internal standard. TBAT crystals are 99% pure.

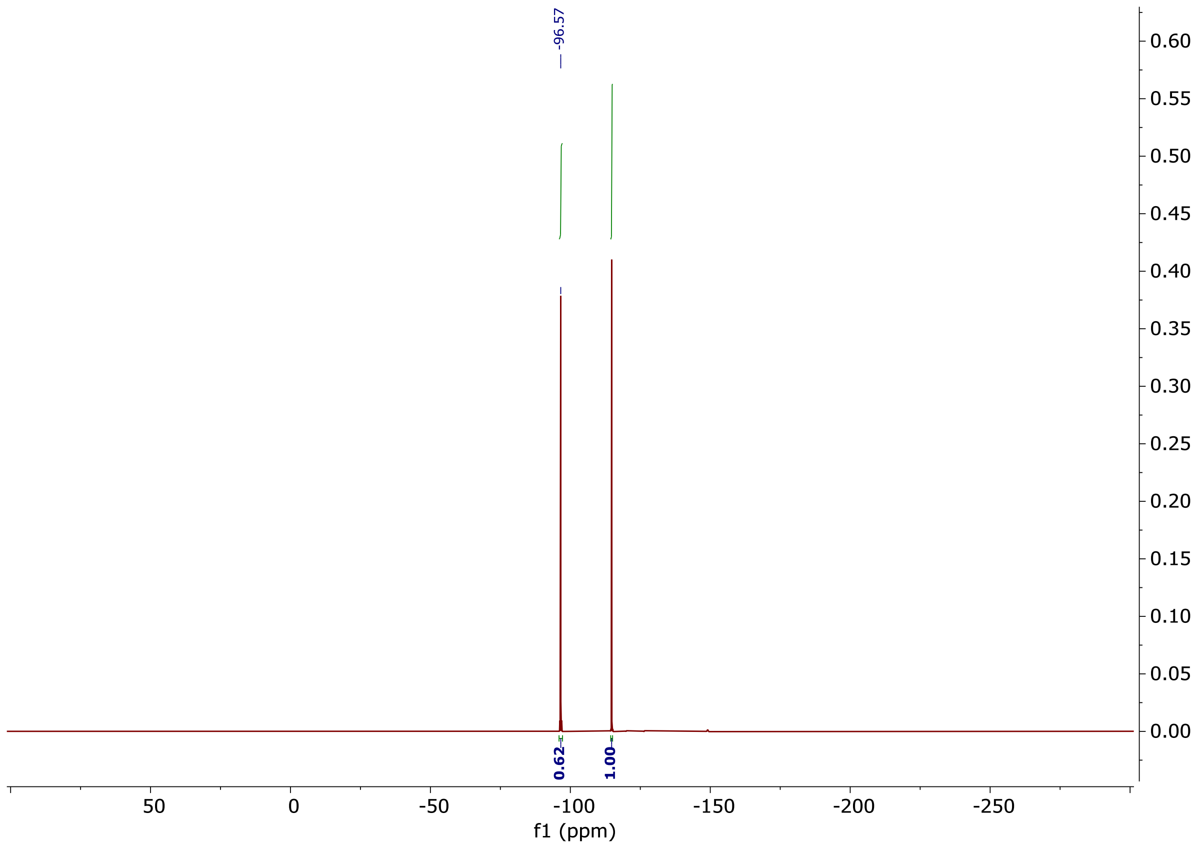

Figure S8.69. Quantitative ^{19}F NMR (376 MHz, dry CD_3CN) of 18.71 mg of $TBAT^{PFAS}$ from perfluorooctane and 10.0 µL of fluorobenzene as internal standard. TBAT crystals are 95% pure.

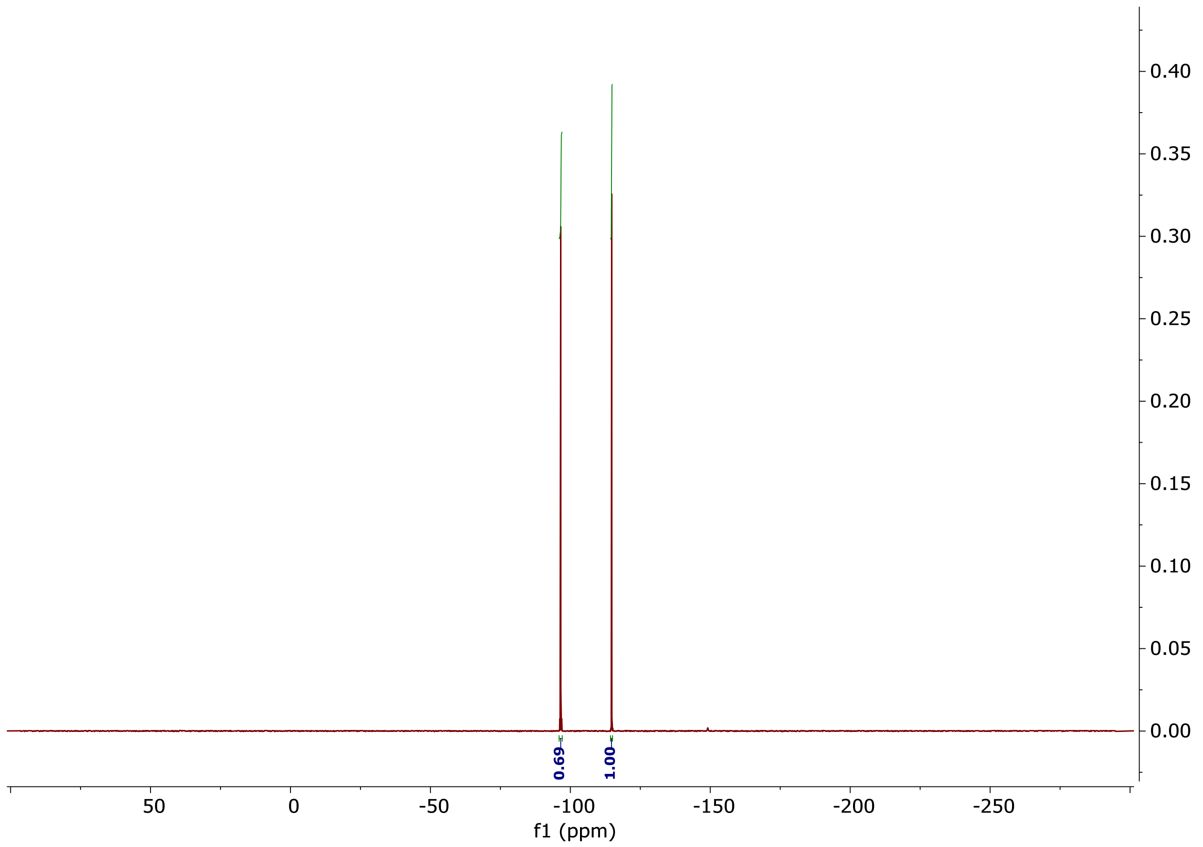

Figure S8.70. Quantitative ^{19}F NMR (376 MHz, dry CD_3CN) of 20.31 mg of $TBAT^{PFAS}$ from 1H,1H,2H-perfluoro-1-decene and 10.0 µL of fluorobenzene as internal standard. TBAT crystals are 98% pure.

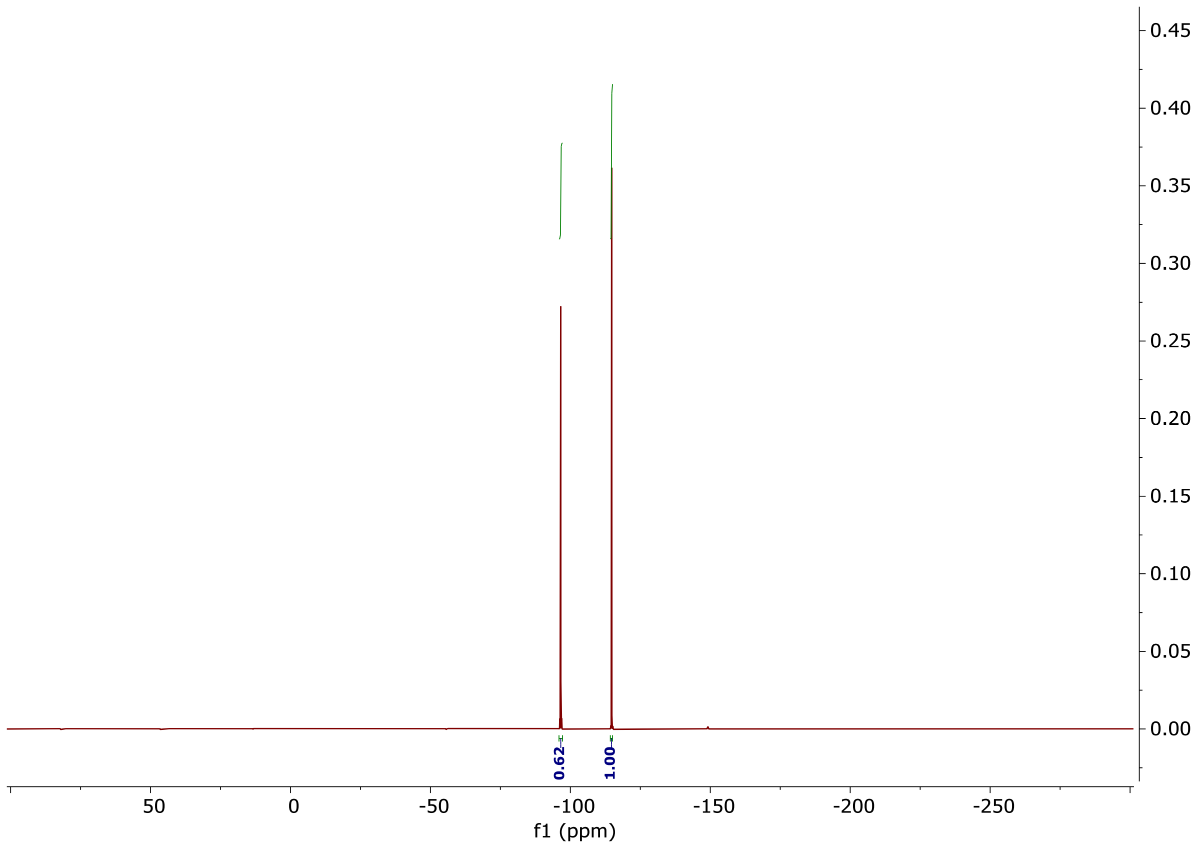

Figure S8.71. Quantitative ^{19}F NMR (376 MHz, dry CD_3CN) of 19.39 mg of $TBAT^{PFAS}$ from PFNA and 10.0 µL of fluorobenzene as internal standard. TBAT crystals are 92% pure.

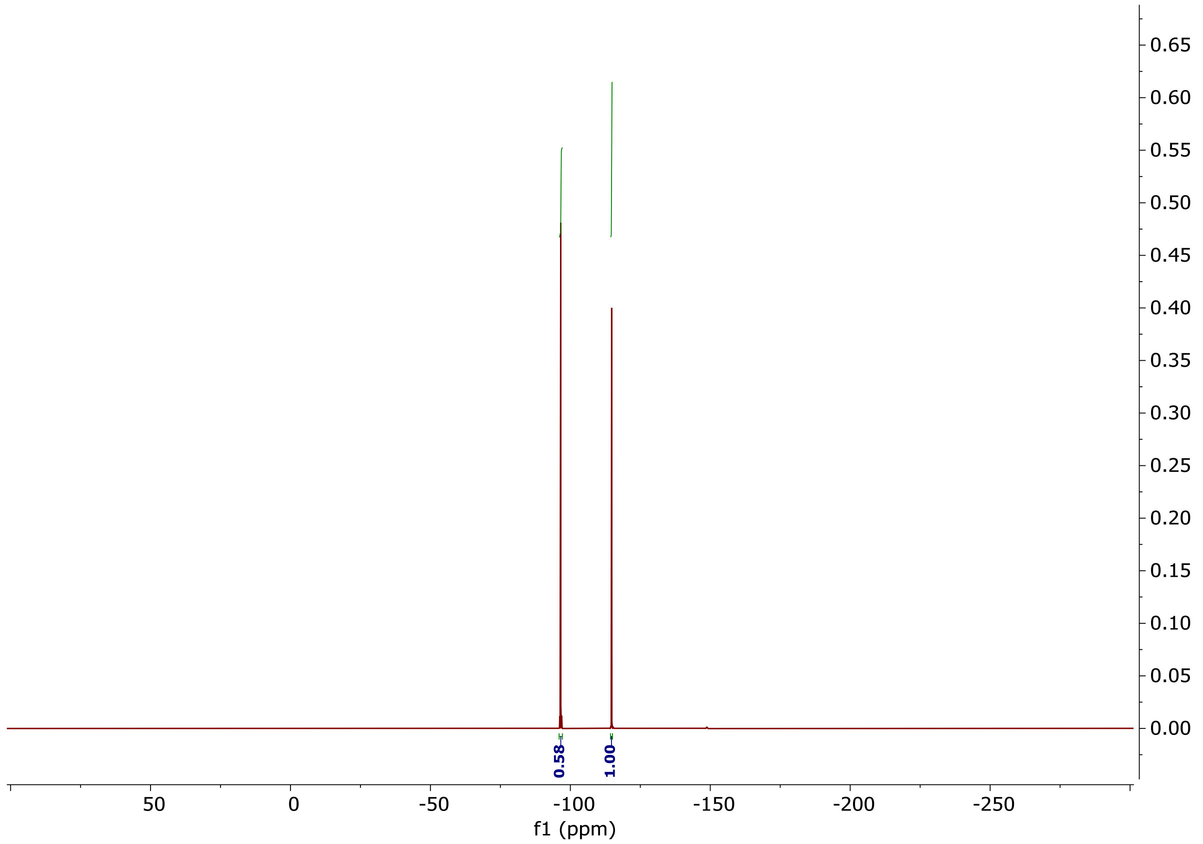

Figure S8.72. Quantitative ^{19}F NMR (376 MHz, dry CD_3CN) of 20.92 mg of $TBAT^{PFAS}$ from PFOS-TBA and 10.0 µL of fluorobenzene as internal standard. TBAT crystals are 80% pure.

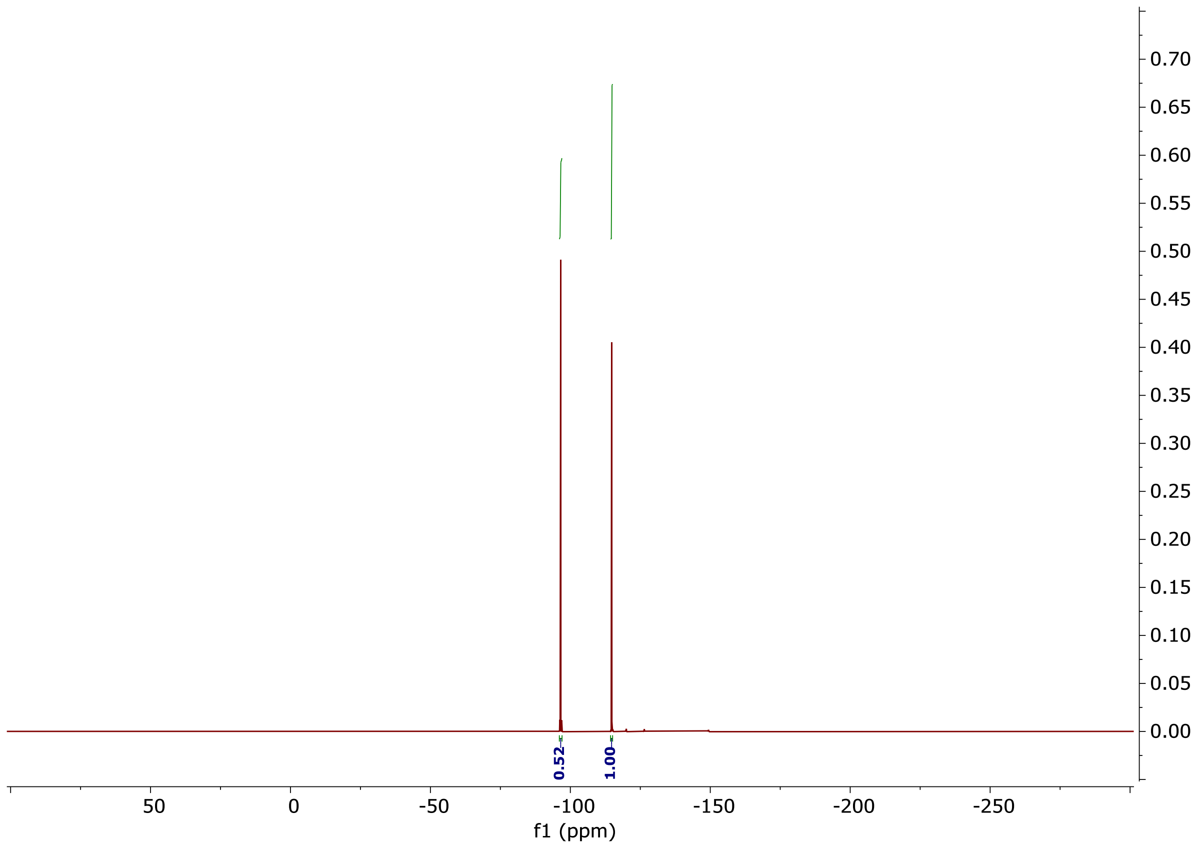

Figure S8.73. Quantitative ^{19}F NMR (376 MHz, dry CD_3CN) of 20.39 mg of TBAT^{PFAS} from PFOA and 10.0 µL of fluorobenzene as internal standard. TBAT crystals are 74% pure.

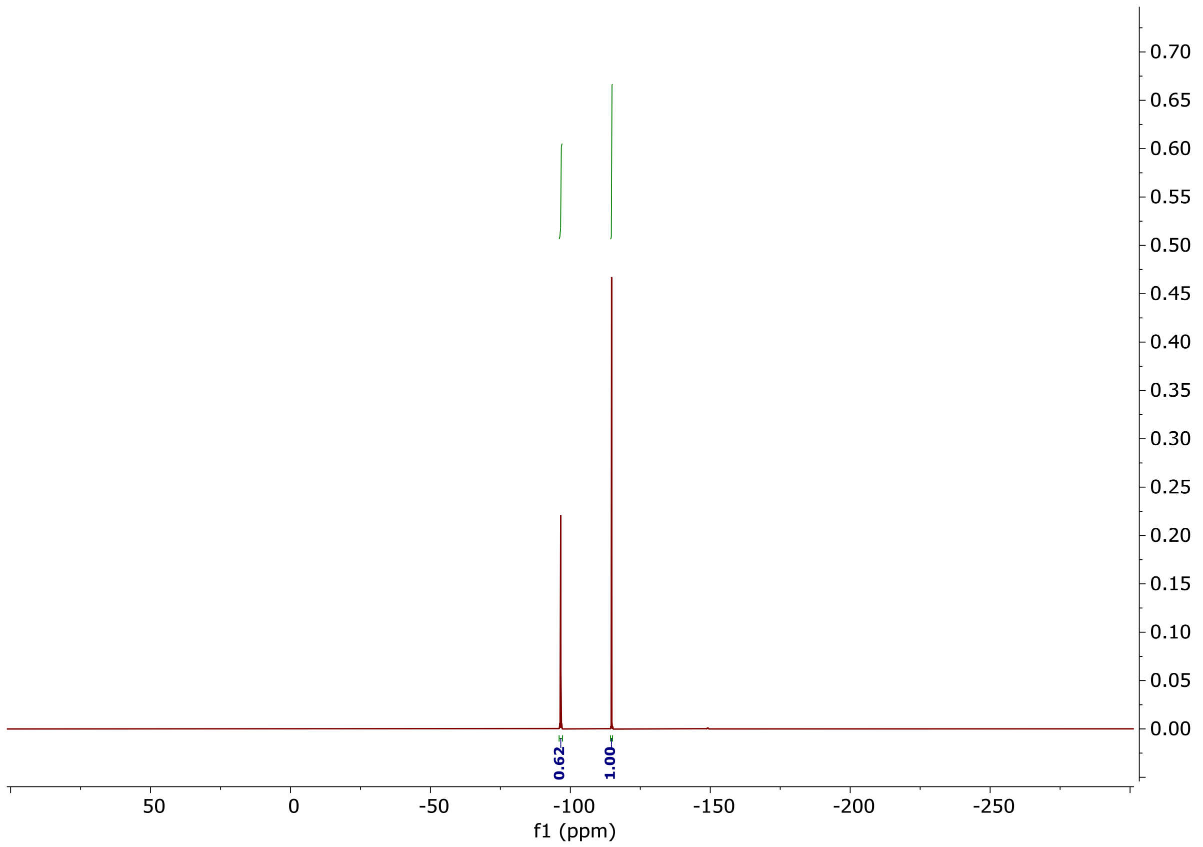

Figure S8.74. Quantitative ^{19}F NMR (376 MHz, dry CD_3CN) of 20.29 mg of $TBAT^{PFAS}$ from HFPO-DA and 10.0 µL of fluorobenzene as internal standard. TBAT crystals are 88% pure.

NMR of recrystallised TBAT.

Given that $CDCl_3$ is not a practical solvent to acquire NMR spectra of TBAT ($CDCl_3$ must be freshly distilled before each NMR because TBAT is degraded by traces of acid in $CDCl_3$),[5] dry CD_3CN (dried over molecular sieves) was used as deuterated solvent. Spectroscopic data is in perfect agreement with that reported in the literature.[5]

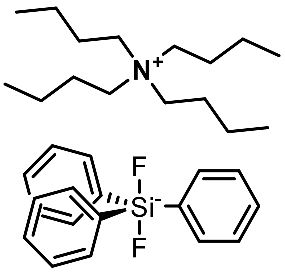

^{1}H NMR (500 MHz, CD_3CN): δ = 7.97 (dd, 6H, J=6.3, 1.7 Hz), 7.24-7.18 (m, 9H), 3.06-3.02 (m, 8H), 1.61-1.54 (m, 8H), 1.40-1.32 (m, 8H), 0.99 (t, 12H, J=7.3 Hz).
^{19}F NMR (470 MHz, CD_3CN): δ = -96.63, $J_{Si\text{-}F}$=252.8 Hz
^{13}C NMR (126 MHz, CD_3CN): δ = 150.9 (t, $J_{C\text{-}F}$=41.61 Hz), 137.1 (t, $J_{C\text{-}F}$=6.7 Hz), 126.5, 126.4, 58.1, 23.3, 19.3, 13.0.
^{29}Si NMR (99 MHz, CD_3CN): δ = -106.16 (t, $J_{Si\text{-}F}$=252.8 Hz).

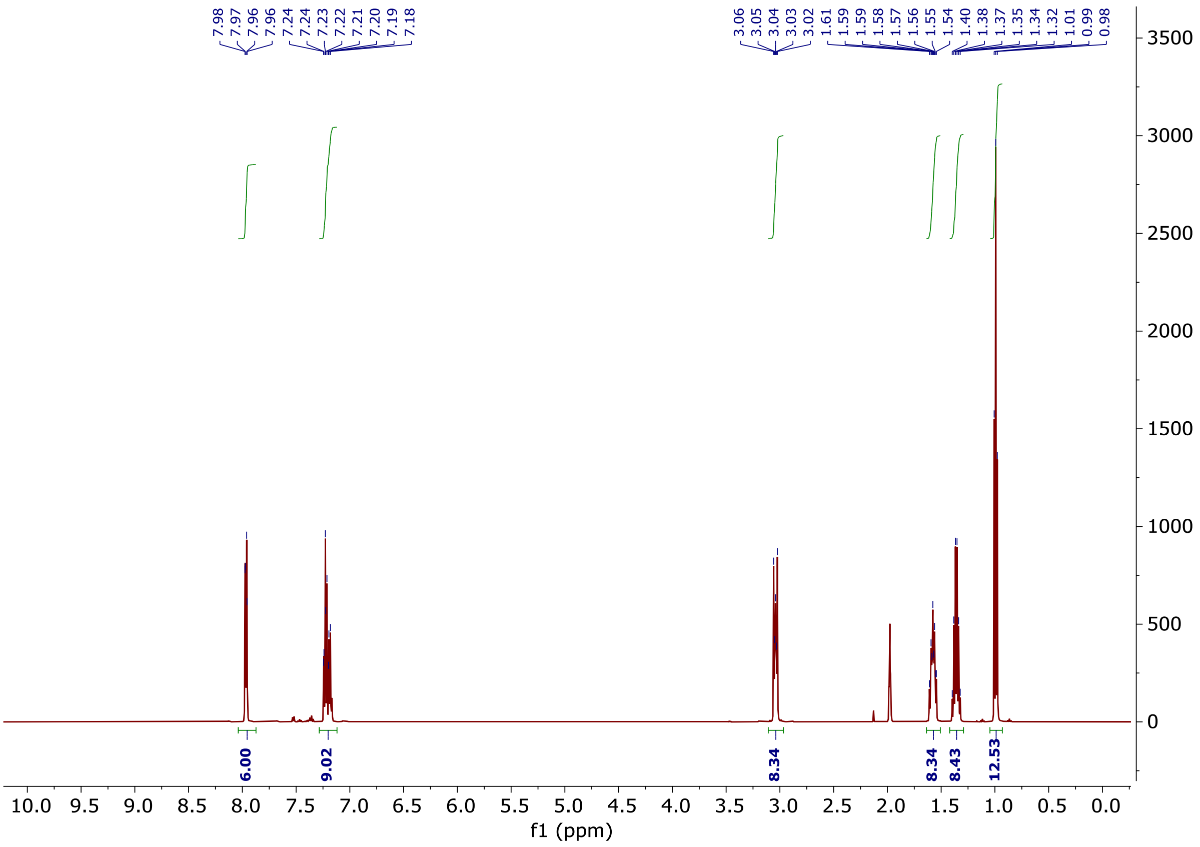

Figure S8.75. ^{1}H NMR (500 MHz, CD_3CN) of recrystallized TBAT. The NMR solvent residual peak is visible at 1.98 ppm and a small water peak is visible at 2.13 ppm.

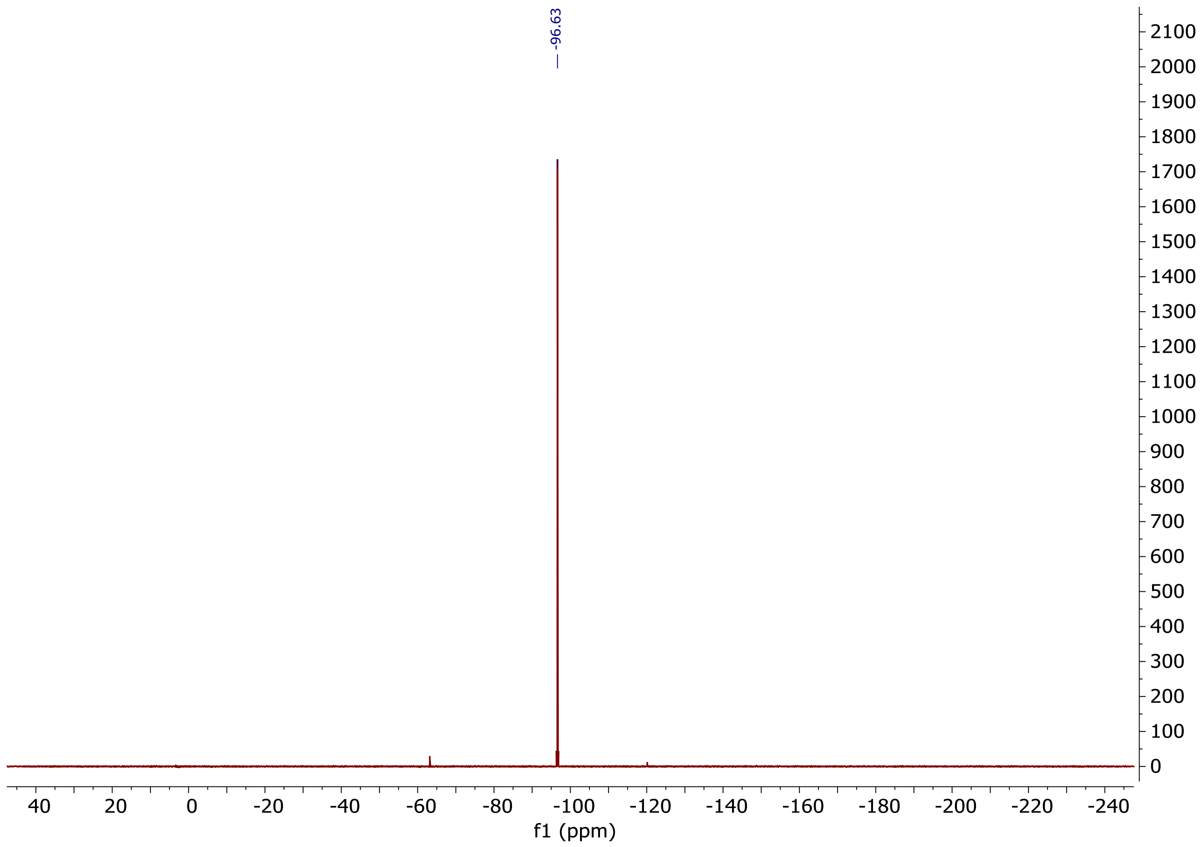

Figure S8.76. ^{19}F NMR (470 MHz, CD_3CN) of recrystallized TBAT. A very small $PhCF_3$ (recrystallization solvent) peak is visible at -63.17 ppm.

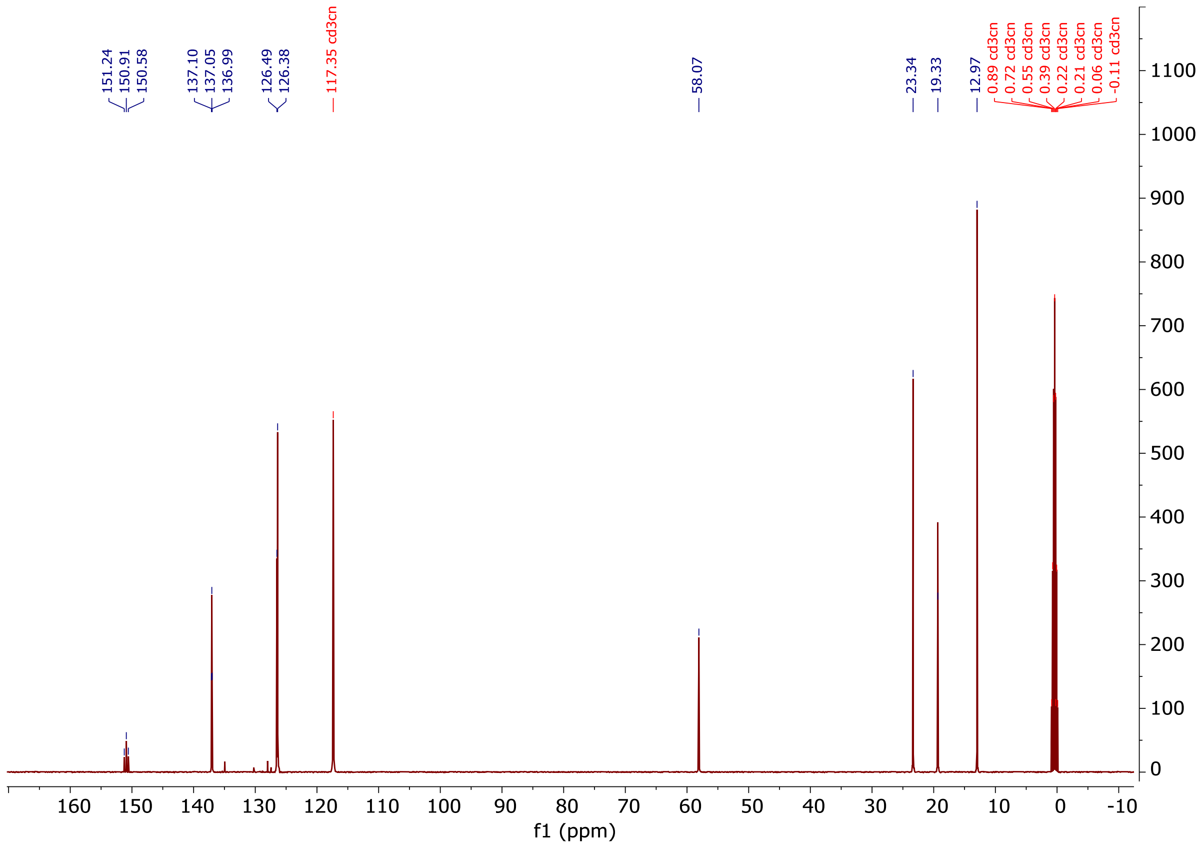

Figure S8.77. ^{13}C NMR (126 MHz, CD_3CN) of recrystallized TBAT. Note: the sample must be concentrated (~ 0.3 M) to obtain an intense-enough signal of the most deshielded aromatic carbon. A relaxation delay of 4s and 2000 scans were used (~ 3h NMR).

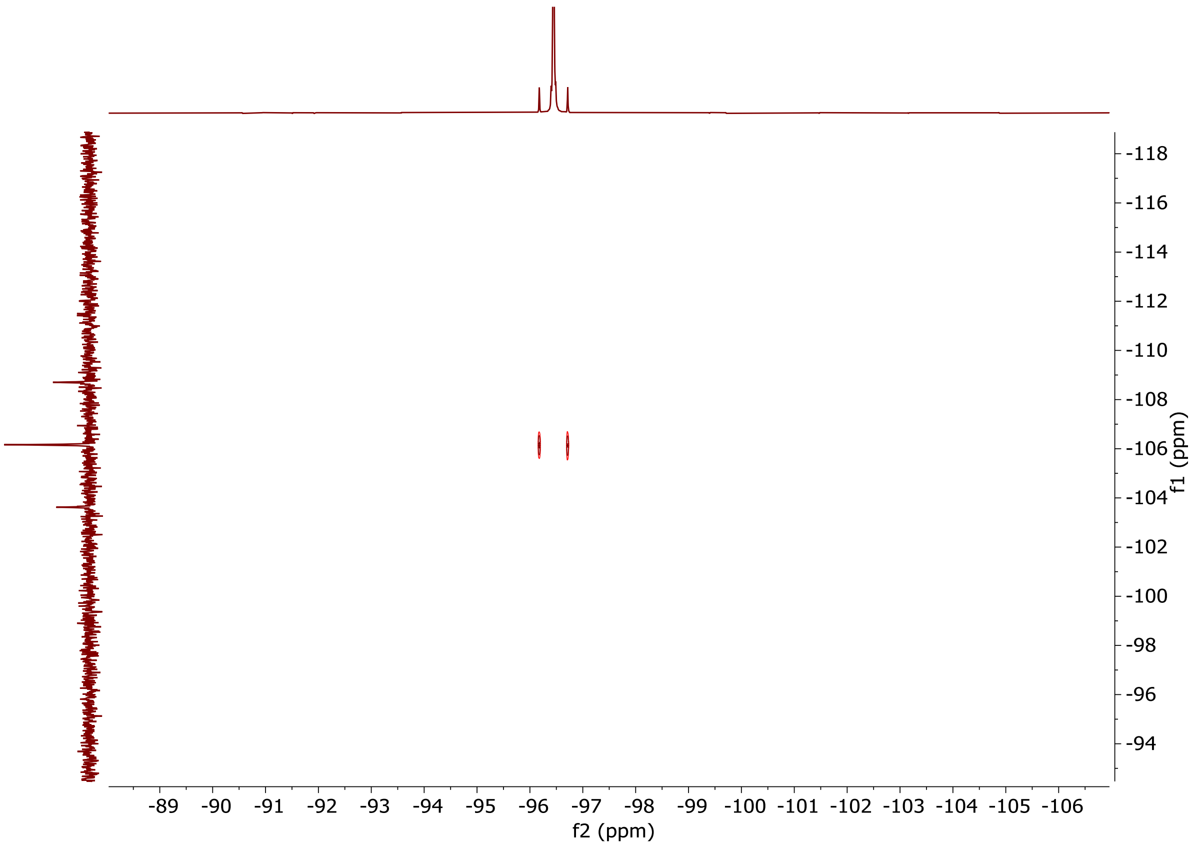

Figure S8.78. ^{19}F-^{29}Si HMBC (CD_3CN) of recrystallized TBAT. Note: the sample must be concentrated (~ 0.3 M) to obtain intense-enough signal.

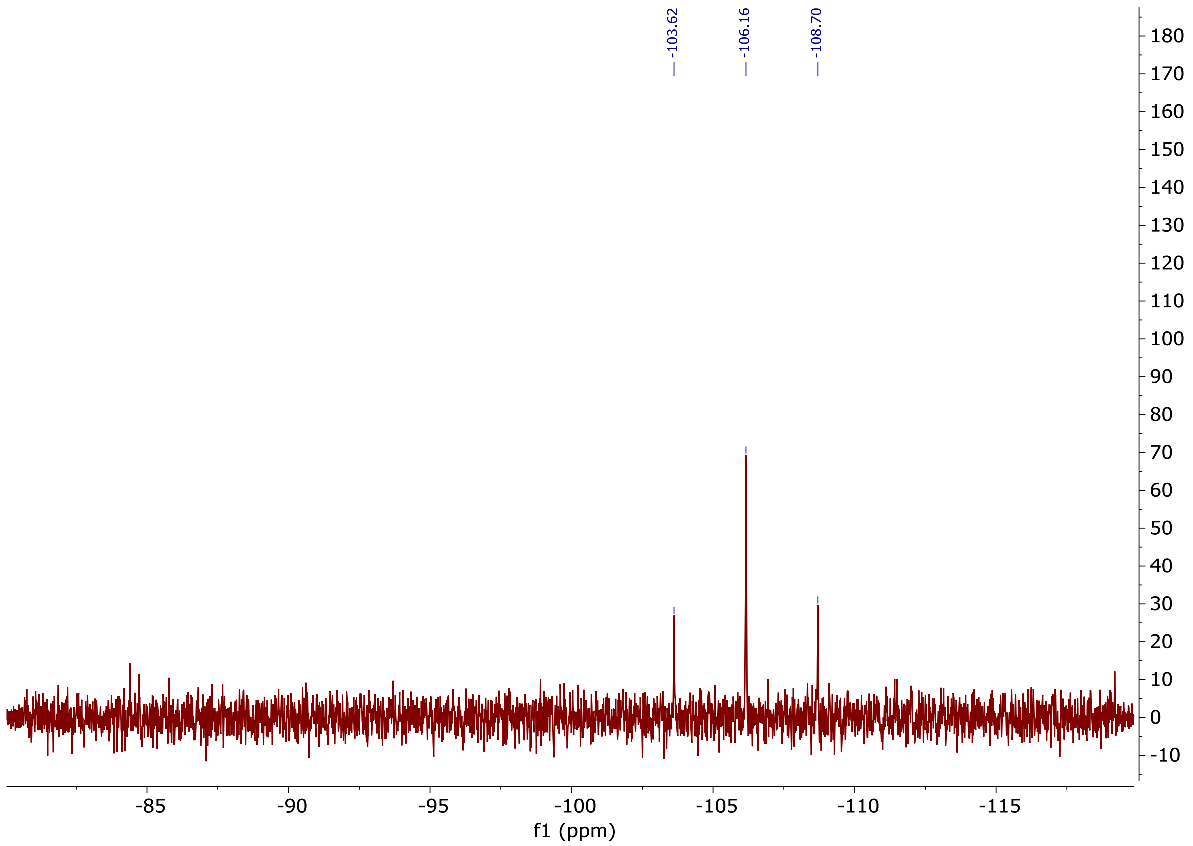

Figure S8.79. ^{29}Si (DEPT) NMR (99 MHz, CD_3CN) of recrystallized TBAT. Note: the sample must be highly concentrated (~ 0.5 M) to obtain intense-enough signal.[5] A relaxation delay of 30s and 1400 scans were used (~ 12h NMR).

9. Electrolysis setups

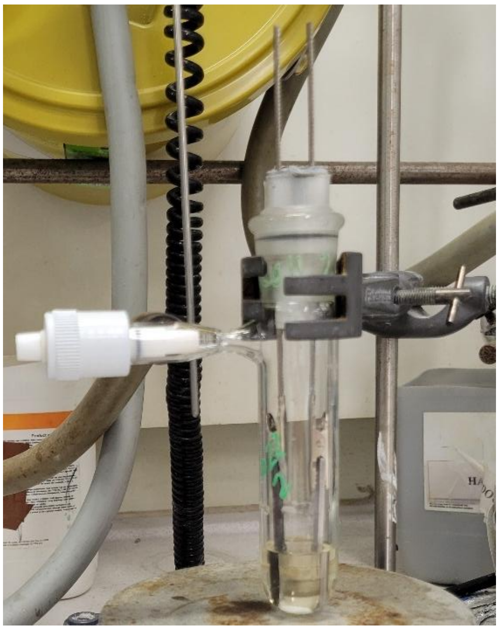

Figure S9.1. Picture of the undivided cell (before starting) used for the TESF synthesis from PFOS-TBA in dry MeCN. The graphite anode is visible on the left and the stainless steel cathode on the right.

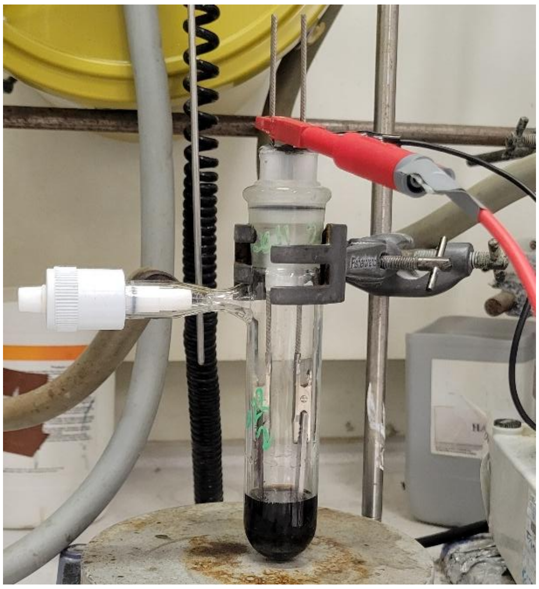

Figure S9.2. Picture of the undivided cell (during electrolysis) used for the TESF synthesis from PFOS-TBA in dry MeCN. The graphite anode is visible on the left and the stainless steel cathode on the right.

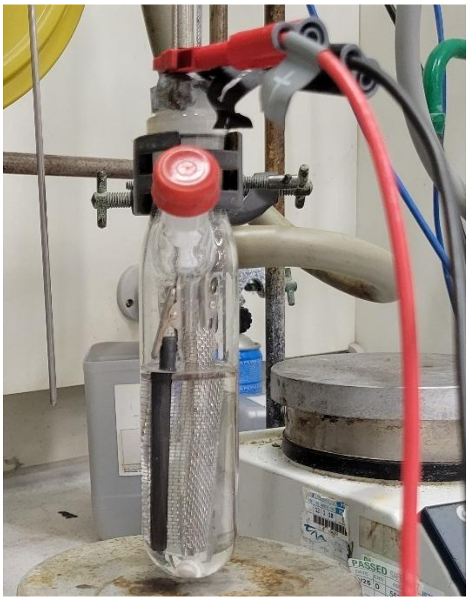

Figure S9.3. Picture of the undivided cell (before start) used for the gram-scale TESF synthesis from perfluorodecalin in dry MeCN. A graphite rod and a stainless steel grid were used as anode and cathode.

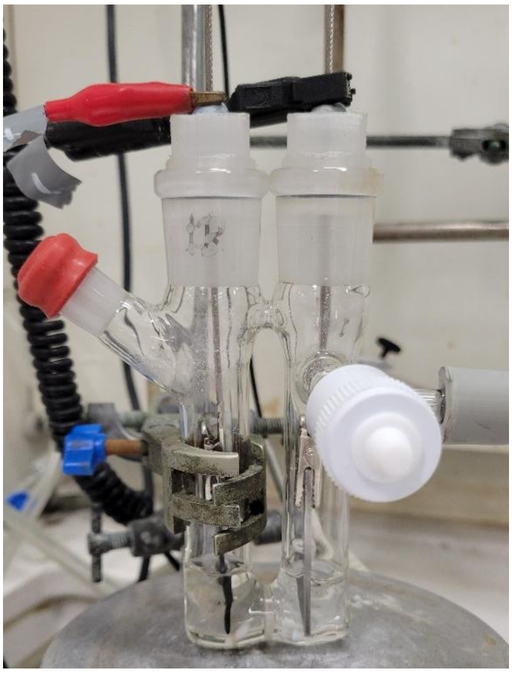

Figure S9.4. Picture of the divided cell (before start) used for the direct electrolysis of perfluorodecalin in dry acetone with TBABr as supporting salt and sacrificial reductant. The anolyte with the graphite anode is visible on the left and the catholyte with the stainless steel cathode on the right.

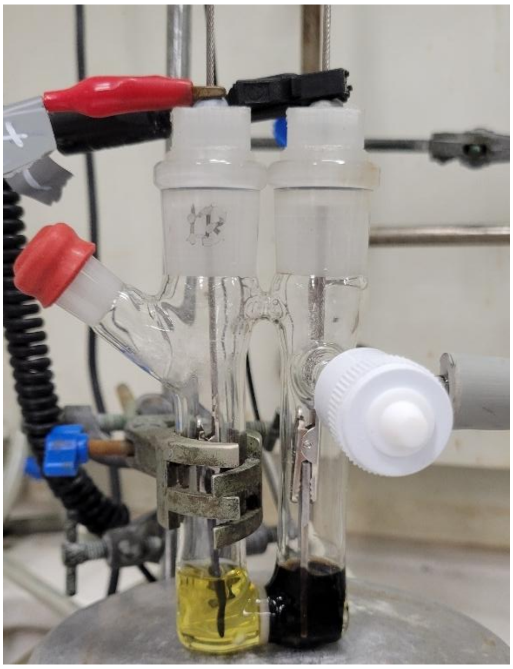

Figure S9.5. Picture of the divided cell (during electrolysis) used for the direct electrolysis of perfluorodecalin in dry acetone with TBABr as supporting salt and sacrificial reductant. The anolyte with the graphite anode is visible on the left and the catholyte with the stainless steel cathode on the right.

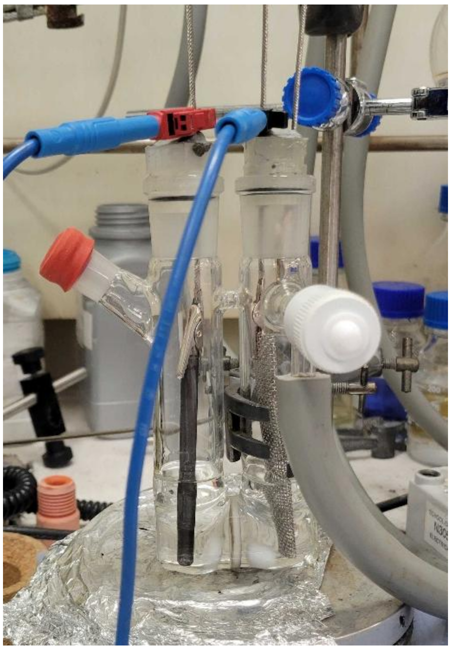

Figure S9.6. Picture of the divided cell (before start) used for the gram-scale TBAT synthesis from perfluorodecalin in dry MeCN. A graphite rod and a stainless steel grid were used as anode and cathode.

Supplementary References

(1) Fan, M.; Jin, Y.; Han, Y.; Ma, L.; Li, W.; Lu, Y.; Zhou, F.; Liu, W. The Effect of Chemical Structure on the Tribological Performance of Perfluorosulfonate ILs as Lubricants for Ti-6Al-4V Tribopairs. *J. Mol. Liq.* **2021**, *321*, 114286. https://doi.org/10.1016/j.molliq.2020.114286.

(2) Eaborn, C. 583. Organosilicon Compounds. Part I. The Formation of Alkyliodosilanes. *J. Chem. Soc.* **1949**, 2755. https://doi.org/10.1039/jr9490002755.

(3) Szmant, H. H.; Miller, G. W.; Makhlouf, J.; Schreiber, K. C. Preparation and Properties of Trialkylfluorosilanes. *J. Org. Chem.* **1962**, *27* (1), 261–265. https://doi.org/10.1021/jo01048a063.

(4) Simpkins, N. S.; Nytko, F. E.; DeShong, P.; Vayer, M.; Maulide, N. Tetrabutylammonium Difluorotriphenylsilicate (TBAT). In *Encyclopedia of Reagents for Organic Synthesis*; Wiley, 2020; pp 1–14. https://doi.org/10.1002/047084289X.rn00469.pub3.

(5) Handy, C. J.; Lam, Y.; DeShong, P. On the Synthesis and NMR Analysis of Tetrabutylammonium Triphenyldifluorosilicate. *J. Org. Chem.* **2000**, *65* (11), 3542–3543. https://doi.org/10.1021/jo991771s.

(6) Wu, N.; Li, C.; Dong, G.; Jiang, M.; Xu, Z. Silver(I)-Catalyzed Oxidative Coupling of Hydrosilanes with DMF to Symmetrical and Unsymmetrical Disiloxanes. *New J. Chem.* **2022**, *46* (10), 4814–4818. https://doi.org/10.1039/d1nj05617d.
